# Benchmark Study of the Electronic States of the LiRb Molecule: Ab Initio Calculations with the Fock Space Coupled Cluster Approach

**DOI:** 10.3390/molecules28227645

**Published:** 2023-11-17

**Authors:** Grzegorz Skrzyński, Monika Musial

**Affiliations:** Institute of Chemistry, University of Silesia in Katowice, Szkolna 9, 40-006 Katowice, Poland

**Keywords:** Fock space multireference coupled cluster method, intermediate Hamiltonian, potential energy curves, spectroscopic constants, LiRb molecule

## Abstract

Accurate potential energy curves (PECs) are determined for the twenty-two electronic states of LiRb. In contrast to previous studies, the applied approach relies on the first principle calculations involving correlation among all electrons. The current methodology is founded on the multireference coupled cluster (CC) scheme constructed within the Fock space (FS) formalism, specifically for the (2,0) sector. The FS methodology is established within the framework of the intermediate Hamiltonian formalism and offers an intruder-free, efficient computational scheme. This method has a distinctive feature that, when applied to the doubly ionized system, provides the characteristics of the neutral case. This proves especially beneficial when investigating PECs in situations where a closed-shell molecule dissociates into open-shell fragments, yet its double positive ion forms closed-shell species. In every instance, we successfully computed continuous PECs spanning the entire range of interatomic distances, from the equilibrium to the dissociation limit. Moreover, the spectroscopic characteristic of various electronic states is presented, including relativistic effects. Relativistic corrections included at the third-order Douglas-Kroll level have a non-negligible effect on the accuracy of the determined spectroscopic constants.

## 1. Introduction

The chemistry and the physics of polar ultracold molecules have been extensively studied in the last decade [1,2,3,4,5]. These types of molecules attract scientists mostly due to their high dipole moment, which induces such phenomena as long-range and anisotropic interactions [6]. An example of a diatomic polar molecule is LiRb, the object of this study. The research finds that the LiRb molecule possesses a dipole moment of around ∼4.2 debye [7] and it has vast potential applications. In 2010, Herrera et al. proposed that the mixture of cold LiRb and LiCs molecules trapped on an optical lattice and controlled using an electric field produces a perfect system for potential applications in quantum simulation, quantum computation, studies of negative refraction of microwave fields, or studies of the formation of wave-vector space crystals of excitons [8]. In 2011, Kuznetsova and her coworkers presented the application of the LiRb molecule as an atom–molecule platform for quantum computing [9]. They discovered that using LiRb, it is possible to encode a qubit in atomic states that allowed an easy initialization, readout, one-qubit operations, and mapping of the qubit state onto a photon for quantum communication. Besides that, LiRb, being a polar molecule composed of alkali metals, can possibly be applied in many fields, e.g., few-body [10] and many-body physics [11], studies of chemical reactions [12], or to expand our knowledge of quantum mechanics itself [13]. These studies have driven us to explore the properties of this molecule more accurately than ever before using the state-of-the-art method [14] described in the Section 3.

The most important experimental works for our study are those concerning potential energy curves (PECs) and/or spectroscopic constants of the LiRb molecule. This consists of the following: two papers by Ivanova et al. in which they used Fourier-transform spectroscopy to evaluate PECs and spectroscopic constants of various states of LiRb [15,16]; the study of Dutta et al. in which they used similar technique [17]; and the studies which were conducted using resonantly enhanced multiphoton ionization spectroscopy [18,19,20,21]. Values obtained in these papers were cited in the Section 2 to evaluate the accuracy of our theoretically obtained data.

In terms of theoretical research, there is a notable number of papers concerning LiRb. These studies may be classified as follows: studies of electric properties [7,22,23,24,25], computations of dispersion coefficients [1,26,27,28,29], calculations of PECs and spectroscopic constants [30,31,32,33,34,35,36,37,38,39,40,41,42,43], and others [44,45,46,47,48,49,50,51]. Among those, the studies of PECs and spectroscopic constants are most relevant in terms of our research.

The current work is focused on the quantum chemical description of the bond-breaking process for LiRb. We have in mind an approach that would be able to provide correct energy values at the dissociation limit as well as an accurate description of the potential energy curves (PECs).

Fundamental information about LiRb can be obtained out of PECs, which allows the evaluation of selected spectroscopic constants of the molecule, e.g., equilibrium distance $R_{e}$, adiabatic excitation energy $T_{e}$, well depth $D_{e}$, etc. The detailed knowledge of PECs in the ground and excited states is crucial in the study of ultracold molecules [52].

A theoretical description is not trivial for alkali metals diatomics using most of the well-established methods. The difficulty comes from the fact that the molecule with a closed-shell character at the equilibrium distance alters into an open-shell system at the dissociation limit. Therefore, the restricted Hartree–Fock scheme (RHF) should not be used for long-range distances in such cases. Usually, the unrestricted Hartree–Fock (UHF) or the restricted open-shell Hartree–Fock (ROHF) methods are used; however, such calculations are often faced with convergence problems of the HF and post-HF solutions. Theoretical chemistry came up with several solutions to this problem, but none of them is ideal. For instance, researchers often use the multireference configuration interaction (MRCI) method, which, in its approximate variants, is not size-extensive. Therefore, they decide to carry out the calculations for valence electrons only, freezing the inner-shells’ electrons, which also affects the accuracy of the results. To overcome this drawback, these electrons are replaced in some studies using the ECP (Effective Core Potential) method, which makes it possible to consider the valence electrons as moving in the potential of atomic cores [36,38,53]. This approximation neglects the subtle effects connected with the correlation of all electrons and, on the other hand, introduces new parameters specific to the considered system to mimic the potential of electrons originating from the inner shells. When discussing interatomic potentials, we should also mention the methods based on the algebraic approaches using the well-known models for interatomic potentials like Morse, Lennard-Jones, or Pöschl–Teller and their usefulness of the characteristic of vibrational spectra [54,55,56,57]. On the other hand, the “method of choice”, frequently applied in the studies of excited states, i.e., EE-EOM-CC (Excitation Energy Equation-of-Motion Coupled Cluster) [58,59,60,61,62], is not size-extensive. This is the major reason that, in the studies of the potential energy curves where the size extensivity plays a prominent role, the latter method is less frequently applied in the calculations of alkali metal dimers [63,64,65].

In our study, we use the Fock space multireference coupled cluster (FS-MRCC) method, which is—on the one hand—size-extensive (an important feature in the PECs calculations) and —on the other—has a built-in capability to provide—by selecting a proper FS sector—correlation corrections for systems with an altered (with respect to the Hartree–Fock (HF) reference) number of electrons. For example, the FS(m,0) sector produces results pertaining to the system with *m* electrons added to the HF function. Assuming a neutral HF reference, these results would correspond to the *m*-tuply negative anion. Smooth and well-behaved PECs are obtained, e.g., in the case of the van der Waals molecules, when the closed shell structure dissociates into the closed shell fragments. This example has been a sort of inspiration when dealing with the problem of the dissociation of alkali metal diatomics. Instead of dissociating the dimer Me${}_{2}$ (Me${}_{2}\to$Me${}^{\cdot }$ + Me${}^{\cdot }$), we can dissociate the doubly ionized molecule Me${}_{2}^{2+}$ according to the following equation:

(1) $$Me_{2}^{2+}\to Me^{+}+Me^{+}$$

and we see here that the closed shell structure Me${}_{2}^{2+}$ dissociates into closed shell fragments, isoelectronic with noble gas atoms, specifically discussing an alkali metal dimer. So, this scheme allows us to obtain continuous and smooth curves for the reference system. In the next step, the FS-MRCC scheme is employed to solve the FS equations for the valence (2,0) sector, recovering the original neutral structure. Consequently, at the correlated level, we obtain results for the Me${}_{2}$ molecule.

The state-of-the-art research is documented with many results obtained for the two-valence sector of the Fock space [14,66,67,68]. Such a strategy, applied to the study of the PECs of a large number of electronic states for a series of alkali metal diatomics [66,67,68], provided the results of incomparable accuracy. The results obtained for the Rb${}_{2}$ dimer (on the basis of the (2,0) sector of Fock space) have already been utilized in the experimental works carried out in ultra-low temperatures [69].

In this study, we present accurately calculated potential energy curves and spectroscopic constants for the 22 lowest-lying electronic states of the LiRb molecule using the FS-CCSD (2,0) (S-Singles, D-Doubles) method in the Intermediate Hamiltonian (IH) formulation [14] to eliminate so-called intruder-state problems [70,71]. Although the structure of the LiRb molecule is similar to that of NaLi, studied in [68], the presence of the heavy Rb atom requires special attention in the choice of the model space and the calculation of the relativistic corrections. The latter effects were included by combining the DK3 (third order Douglas–Kroll) [72] formalism and the IH-FS-CCSD (2,0) scheme [14]. The local version of the IH-FS-CCSD (2,0) program was connected to the GAMESS (General Atomic and Molecular Electronic Structure System) package. This required the development of the necessary software [68].

Obtained results are presented in the Section 2, followed by the more detailed method description in the Section 3, and the study is summarized in the Section 4.

## 2. Results and Discussion

All calculations were performed using ACES II [73] ver. 2.7.0 and GAMESS [74] ver. 2021 R2 Patch 1 packages, both supplemented with our own modules in the local version for IH-FS-CCSD(2,0) calculations [14,68]. The spectroscopic constants were computed using 8.0 version of Robert J. LeRoy’s LEVEL program [75]. For the main calculations, we used the uncontracted ANO-RCC [76] basis set with additional diffuse functions, which we called unANO-RCC+. The exponents for the six additional diffuse functions for the lithium are as follows: 0.0027497, 0.0009619 for the s shell; 0.0017173, 0.0006010 for the p shell; and 0.0067528, 0.0023635 for the d shell. Analogously, for the rubidium atom, the exponents for the new diffuse functions are as follows: 0.0012643, 0.0005057, 0.0002023, 0.0000809 for the s shell; 0.0034222, 0.0013689, 0.0005476, 0.0002190 for the p shell; and 0.0727457, 0.0327356, 0.0147310, 0.0066289 for the d shell. We have obtained the exponents using the even-tempered scheme to provide the correct ordering of atomic states. The final size of unANO-RCC+ basis set was 326 basis functions. The spherical harmonic polarization functions were used in all of our calculations and all electrons were correlated.

To evaluate the contribution of relativistic effects in spectroscopic constants of the LiRb molecule, we used the following scheme: we computed potential energy curves and spectroscopic constants of LiRb using Sapporo-QZP-2012-diffuse basis set [77] (167 basis functions), and, in the next step, our calculations were again completed using the scalar relativistic corrections of Douglass–Kroll third order (DK3) [72] and applying the Sapporo-DKH3-QZP-2012-diffuse basis set (also the same number of basis functions), which is the relativistic equivalent of the former basis set. Finally, we calculated the differences between the relativistic and non-relativistic results and these values were added to the constants obtained using the unANO-RCC+ basis set since our previous works show that spectroscopic constants derived from PECs calculated with the family of Sapporo basis sets are not as accurate as those derived from PECs obtained using augmented ANO-RCC basis sets [68,78].

As a reference system for all of the double electron attachment calculations, we used the LiRb${}^{2+}$ cation. The reference function was always RHF. The size of the active space was set to 91 (i.e., 91 lowest virtual orbitals have been selected as active) for IH-FS-CCSD(2,0)/unANO-RCC+ calculations, which resulted in the model space size equal to 8281. For IH-FS-CCSD(2,0)/Sapporo-QZP-2012-diffuse, as well as for IH-FS-CCSD(2,0) DK3/Sapporo-DKH3-QZP-2012-diffuse calculations, the active space size was set to 50 (model space size = 2500).

The dipole moment μ of the ground state of LiRb was calculated using the numerical Finite Field approach [79]. We used the following equation:

(2) $$\mu =-\frac{E(F)-E(-F)}{2F},$$

where E(F) is the energy of the system in the weak uniform external electric field *F*.

The value of *F* was equal to 0.001 a.u. and its vector was aligned with the $C_{\infty }$ axis of the molecule. The external electric field perturbation in IH-FS-CCSD(2,0) computations was introduced using ACES II. In order to estimate the contribution of relativistic effects for the dipole moment, we used a similar procedure as in the case of spectroscopic constants, this time using the difference between CCSD/Sapporo-QZP-2012-diffuse and CCSD/Sapporo-DKH3-QZP-2012-diffuse + DK3 results. We used OpenMolcas quantum chemistry software package [80], which allowed us to combine both an external electric field and DK3 scalar relativistic correction.

### 2.1. Atomic Energies at the Dissociation Limit

The important feature of the IH-FS-CCSD(2,0) method is its size-extensivity. This property enforces that the energies of electronic states of the LiRb molecule should converge at an infinite distance to the sum of atomic values. Table 1 presents computed values in the unANO-RCC+ basis set. The first and the second columns present values of energies of Li and Rb—these were calculated using EA-EOM-CCSD—which is equivalent to the IH-FS-CCSD(1,0) method. The third column presents the sum of those energies, and the fourth column shows the energy in the respective dissociation limit obtained using the IH-FS-CCSD(2,0) method. We can clearly see that the results are equal and our method is strictly size-extensive. Thus, the FS-CC method shows correct separability. This feature is particularly useful in the studies of the dissociation process.

### 2.2. Dipole Moment

The calculated dipole moment of the ground state of the LiRb molecule is presented in Table 2 alongside experimental and theoretical reference values. The LiRb molecule has one of the largest dipole moments among heteronuclear alkali metal dimers, being second only to the species containing cesium [7], and our results comply with this fact. Our value computed using IH-FS-CCSD(2,0) DK3/unANO-RCC+ is very close to the experimental values of Tarnovsky et al. [81] and Stevenson et al. [21]. We can clearly see that the methods based on pseudopotentials usually overestimate the values of the dipole moment of LiRb, and our results are close to the quality of CCSDT [27,40] with significantly smaller computational effort needed to solve the equations.

### 2.3. Potential Energy Curves

Potential energy curves of LiRb were calculated using the IH-FS-CCSD(2,0)/unANO-RCC+ method for the 22 lowest-lying electronic states. These are the states correlating to six dissociation limits: Li(2s) + Rb(5s) – two states ($1^{1}\Sigma^{+}$, $1^{3}\Sigma^{+}$), Li(2s) + Rb(5p) – four states ($2^{1}\Sigma^{+}$, $2^{3}\Sigma^{+}$, $1^{1}\Pi$, $1^{3}\Pi$), Li(2p) + Rb(5s) – four states ($3^{1}\Sigma^{+}$, $3^{3}\Sigma^{+}$, $2^{1}\Pi$, $2^{3}\Pi$), Li(2s) + Rb(4d) – 6 states ($4^{1}\Sigma^{+}$, $4^{3}\Sigma^{+}$, $3^{1}\Pi$, $3^{3}\Pi$, $1^{1}∆$, $1^{3}∆$), Li(2s) + Rb(6s) – two states ($5^{1}\Sigma^{+}$, $5^{3}\Sigma^{+}$), Li(2s) + Rb(6p) – four states ($6^{1}\Sigma^{+}$, $6^{3}\Sigma^{+}$, $4^{1}\Pi$, $4^{3}\Pi$). Some molecular states, engaging the *p* or *d* atomic levels, are degenerated (Π and ∆).

**Figure 1:**
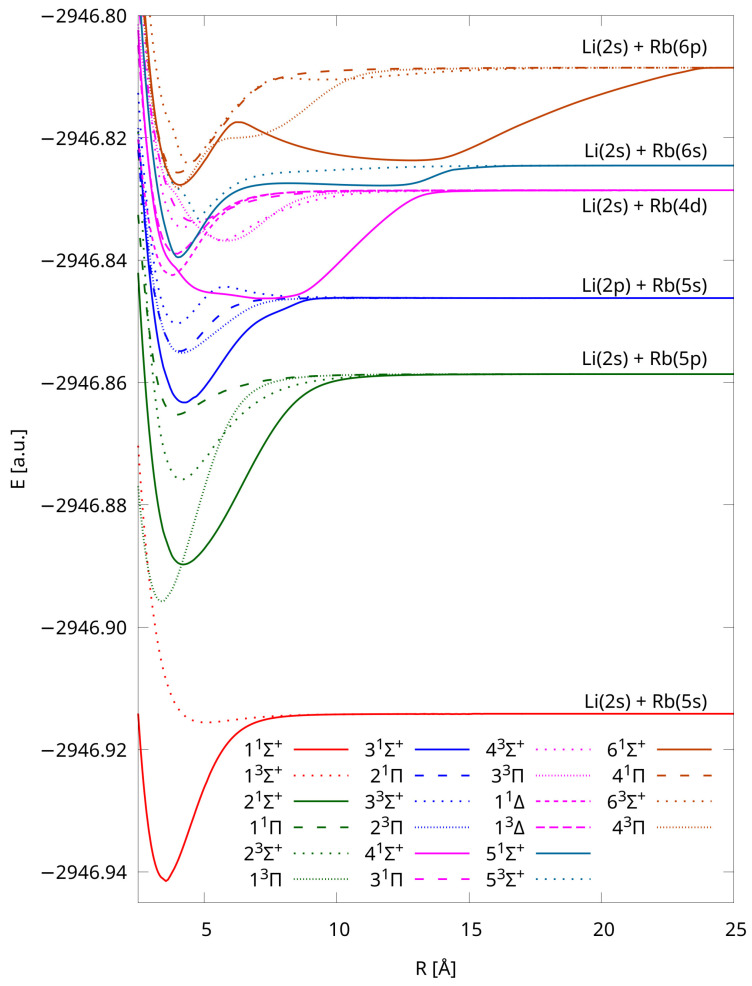
Potential energy curves of LiRb calculated using the IH-FS-CCSD(2,0)/unANO-RCC+ method for the six lowest dissociation limits.

**Figure 2:**
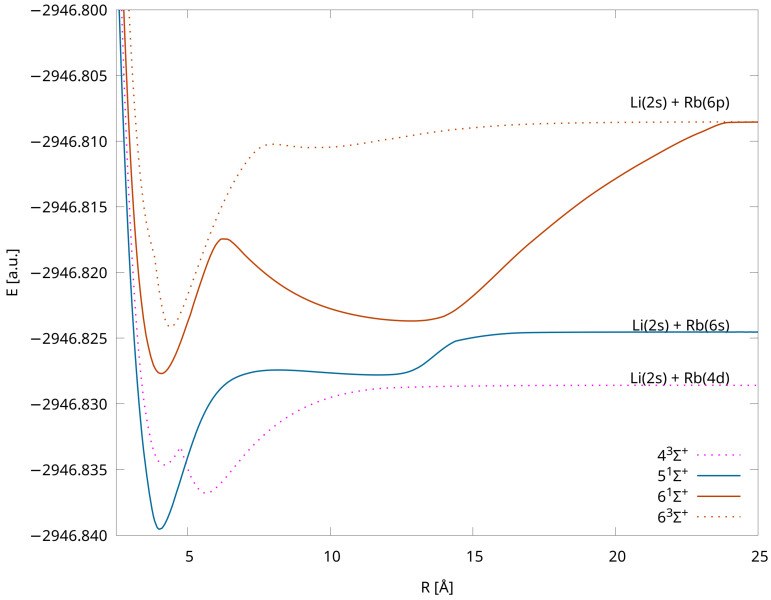
Potential energy curves for the LiRb molecule with the IH-FS-CCSD (2,0) method for states with double minima in the unANO-RCC+ basis set.

The computed PECs are shown in Figure 1—we chose separate point types for each symmetry and multiplicity, and states assigned to different dissociation limits have distinct colors. The applied computational method, which uses the closed shell configuration as the reference, has the advantage that the calculations can be completed equally easily for any internuclear distance. The total molecular energies of each state for the various interatomic distances are available in the Supplementary Materials. All of the states are bound, and four of them have double minima, i.e., $4^{3}\Sigma^{+}$, $5^{1}\Sigma^{+}$, $6^{1}\Sigma^{+}$, $6^{3}\Sigma^{+}$ (see Figure 2), which is in accordance with the results obtained in Ref. [38] via pseudopotential calculations. The energy barriers/positions are 300 cm${}^{-1}$/4.75 Å, 2314 cm${}^{-1}$/8.05 Å, 2214 cm${}^{-1}$/ 6.20 Å, and 3057 cm${}^{-1}$/8.00 Å, respectively (see Table 3).

The higher-lying states have a specific form due to avoided crossing (see Table 4 and Figure 1 and Figure 2).

**Figure 3:**
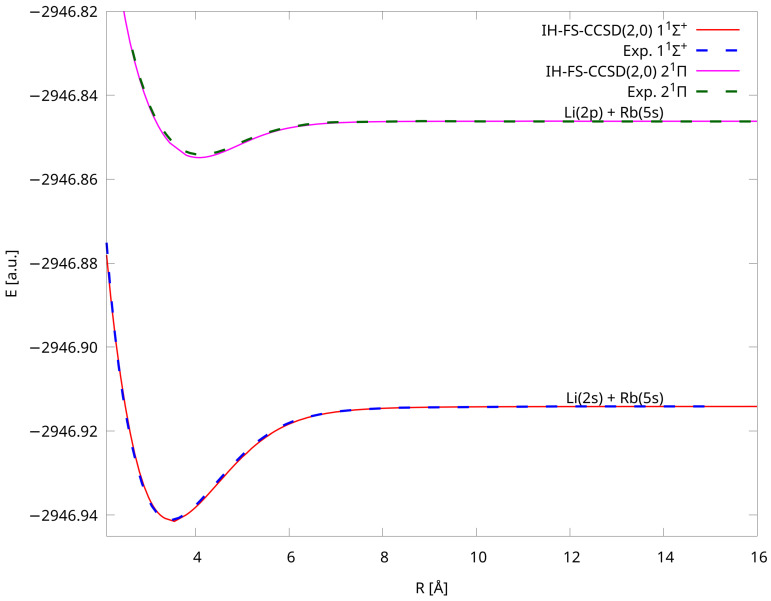
Comparison of PECs of LiRb for the $X^{1}\Sigma^{+}$ and $2^{1}\Pi$ states calculated using the IH-FS-CCSD(2,0)/unANO-RCC+ method with experiment (see [15,16] for experimental data).

We also present Figure 3 in which the comparison with experimental curves of Refs. [15,16] is shown. We were able to compare our theoretical PECs with the experimental ones in two cases: $1^{1}\Sigma^{+}$ and $2^{1}\Pi$. We can see the exceptional alignment of our IH-FS-CCSD(2,0) values with the experimental ones. Although the experimental data are accessible only in a limited range, we can see that the respective parts overlap each other very well. This accuracy is also revealed in spectroscopic constants, which are described in the next subsection. In addition, we computed the C${}_{6}$ coefficient for the ground state and obtained a value of 2457 a.u. comparing well with the experimental one equal to 2550 a.u. [82].

**Figure 4:**
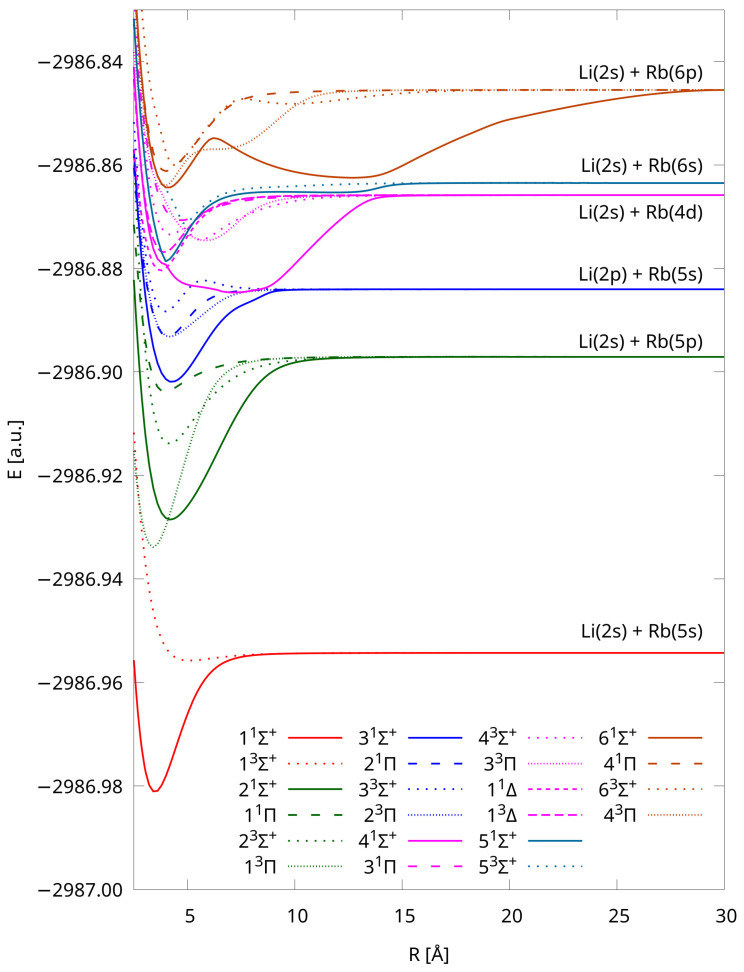
Potential energy curves of LiRb calculated using the IH-FS-CCSD(2,0) DK3/Sapporo-DKH3-QZP-2012-diffuse method for the six lowest dissociation limits.

Finally, just for comparison purposes, we also present PECs calculated using IH-FS-CCSD(2,0) DK3/Sapporo-DKH3-QZP-2012-diffuse (see Figure 4). As we can see, the shapes of the curves are similar to those obtained using a significantly larger unANO-RCC+ basis set (Figure 1).

We observe, and this is an important feature of the results, that the curves collected in Figure 1 and Figure 4 dissociate for each group into a common limit, and the shapes of our PECs are consistent with those available from the literature [38].

### 2.4. Spectroscopic Constants

The spectroscopic constants of the LiRb molecule are shown in Table 5. These include equilibrium distance $R_{e}$, well depth $D_{e}$, adiabatic excitation energy $T_{e}$, harmonic frequency $\omega_{e}$, anharmonicity constant $\omega_{e}x_{e}$, and equilibrium rotational constant $B_{e}$. The data obtained in the current work include the relativistic contributions, while the pure relativistic corrections are shown in parentheses. In addition to our IH-FS-CCSD(2,0) results, we present values obtained in previous papers for comparison purposes. In order to undertake reliable analysis of our results, we decided to limit the number of cited references to fairly recent and the most relevant theoretical works, i.e., the paper of Berriche et al. in which they use a well-established method based on pseudopotential [38], the two publications of You et al., which apply multireference configuration interaction method (MRCI) [41,42], and the recent article of Kozlov et al. in which they combine effective core potentials with the relativistic variant of FS-CCSD method [43]. Also, we present the experimental values of Refs. [15,16,18,19].

Out of the results cited in the Table 5, we were able to perform a statistically significant mean absolute error (MAE) analysis of experimental values only with the paper by Jendoubi et al. [38]. For $T_{e}$, $D_{e}$ and $\omega_{e}x_{e}$, our results agree better with experimental values, while for $R_{e}$, the results in the cited paper are better. The value of $\omega_{e}$ is similar (the difference is about 1.7%). This is the expected behavior—pseudopotential-based methods produce quite accurate results near the equilibrium distance but are not size-extensive, therefore cannot properly reproduce the PECs for the distances far from the minimum, in contrast to our IH-FS-CCSD(2,0) method, which behaves properly in the whole spectrum of interatomic distances. Also, for the case of the ground state of LiRb, our results are closer to the experimental values of [15], with the perfect alignment of the $R_{e}$ value.

For the ground state, our method provides results more consistent with the multireference configuration interaction (MRCI) method [41,42] than with the method based on pseudopotential [38]. For the other states where MRCI results are available, it is not always the case, i.e., a part of our results is closer to the pseudopotential method. We were also able to compare our results to the relativistic variant of FS-CCSD method by Kozlov et al. [43] for $2^{1}\Sigma^{+}$ and $1^{3}\Pi$ states. The alignment is exceptionally good with the difference of less than 0.2%, except of the $R_{e}$ value for the $1^{3}\Pi$ state where the difference is slightly higher but still less than 2%.

We have correctly identified $4^{3}\Sigma^{+}$, $5^{1}\Sigma^{+}$, $6^{1}\Sigma^{+}$, $6^{3}\Sigma^{+}$ states as having double minima, which we confirmed by comparison with the study of Jendoubi et al. [38]. Generally, the agreement of $R_{e}$, $D_{e}$, and $T_{e}$ values with the cited paper is good. The best agreement of $R_{e}$ can be seen for the singlet Π and ∆ states, in the case of $D_{e}$ for the $4^{1}\Pi$ state (the difference of 0.4%), and the absolute difference in $T_{e}$ for the second minimum of $5^{1}\Sigma^{+}$ state is only 10 cm${}^{-1}$. The good match in $R_{e}$ implies the same for the derived $B_{e}$ values. However, $\omega_{e}$ have some exceptions where the differences are larger than expected, i.e., $4^{1}\Sigma^{+}$, $4^{3}\Sigma^{+}$, and $4^{3}\Pi$.

Relativistic corrections given in parentheses in the Table 5 are not regular—they fluctuate between states and spectroscopic constants. In some cases, they contribute less than 1% of the value of the given spectroscopic constant (e.g., $T_{e}$ for $1^{3}\Sigma^{+}$ or $R_{e}$ for $1^{3}∆$), while in others, they contribute more than 50% of the computed value (e.g., 506 vs. 915 cm${}^{-1}$ for $D_{e}$ of $3^{3}\Sigma^{+}$ state). Rubidium is an atom where relativistic effects cannot be neglected. Thus, their contribution to the energies of the LiRb dimer should always be included.

## 3. Methods

Within the single reference (SR) coupled cluster (CC) theory, the wave function is defined via exponential Ansatz [83,84]

(3) $$|\Psi \rangle =e^{T}|\Phi_{0}\rangle$$

where *T* is a cluster operator defined at the CCSD level as follows:

(4) $$T=T_{1}+T_{2}=\sum_{a,i}t_{i}^{a}a^{†}i+\frac{1}{2}\sum_{ab,ij}t_{ij}^{ab}a^{†}b^{†}ji$$

Operators *T*${}_{1}$, *T*${}_{2}$ are responsible for single, double excitations from the reference function $|\Phi_{0}\rangle$. It should be stressed that in our calculations, the reference system is understood as a doubly ionized structure LiRb${}^{+2}$, and both RHF and CCSD solutions are referred to this reference. The a...i... are the second-quantized operators, removing an electron from the occupied level *i* and placing it on the virtual one *a*. The $t_{i\cdot }^{a\cdot }$ amplitudes are the solution of the CC equations obtained by a projection of the $\bar{H}\left(\equiv e^{-T}He^{T}\right)$ operator against excited configurations $\langle \Phi_{i...}^{a...}|$:

(5) $$\langle \Phi_{i...}^{a...}|e^{-T}He^{T}|\Phi_{0}\rangle =\langle \Phi_{i...}^{a...}|\bar{H}|\Phi_{0}\rangle =0$$

The multireference [85,86,87,88,89,90,91,92,93,94,95,96,97,98,99,100,101,102] formalism assumes that the configurational space is divided into two subspaces: the model space M with the projector operator *P* and its orthogonal complement $M_{⊥}$ defined by the projector *Q*. The advantage of the FS-MRCC approach lies in the fact that the diagonalization of the Hamiltonian *H* within full configurational space (size: millions, billions,...) is replaced by the diagonalization of the effective Hamiltonian $H_{eff}$ operator in the model space. The size of the model space is drastically smaller and the diagonalization of the $H_{eff}$ can be carried out in most cases with the standard diagonalization techniques.

The effective Hamiltonian is defined as follows:

(6) $$H_{eff}=PH\Omega P$$

where Ω is a wave operator, which, on acting on the model function $\Psi_{0}$, generates an exact wave function:

(7) $$\left|\Psi \rangle =\Omega \right|\Psi_{0}\rangle$$

In order to use particle-hole second-quantized operators, the configuration adopted as a Fermi vacuum must be selected. Then, the model determinants can be generated by the action of the particle-hole creation operators within the valence one-particle levels. The *K*-valence space is defined by indication of the $N_{v}$ valence electrons and $n_{v}$ valence unoccupied levels ($K=N_{v}+n_{v}$).

The MR-CC considered here, i.e., formulated in the Fock space (also known as a valence universal), admits the configuration with a variable number of electrons. The FS model is obtained by a distribution of 0,1,2,...,K valence electrons among K valence orbitals. It is obvious that it includes configurations containing 0 valence electrons, i.e., with $N_{v}$ electrons removed from the system, as well as K valence electrons, i.e., $n_{v}$-tuply ionized anion. The valence universality means that i) all model configurations are defined with respect to the same Fermi vacuum; ii) the wave operator Ω is defined identically for all reference determinants.

An important step in the FS approach relates to the selection of the Fermi vacuum, which—on the one hand—defines the reference system for which the one-particle states are obtained (in our approach, the Hartree–Fock solutions) and—on the other—determines the sector structure of the model space. The sector is defined by the number of valence particles and valence holes created with respect to the vacuum, e.g., (k,l) sector indicates that the reference configuration contains *k* valence particles and *l* valence holes. The (0,0) sector indicates the Fermi vacuum.

The flexibility of the FS approach—indicated already in the Section 1—can be used in all situations where we want to replace the open-shell reference (requiring UHF function) with the closed shell one, conveniently described by the RHF method.

The basic general formula for the MR approach is a Bloch equation of the form

(8) $$H\Omega P=\Omega PH\Omega P=\Omega PH_{eff}P$$

Operating from the left with the *P* operator, we obtain the expression for the $H_{eff}$ ($H_{eff}=PH\Omega P$) (the identity PΩ=P has been used) while the projection against the configurations belonging to the orthogonal subspace (represented by the *Q* operator) generates MR-CC equations. The Ω operator can be expressed as

(9) $$\begin{array}{c} \Omega =\left\{e^{\tilde{\tilde{S}}}\right\}P=\left\{e^{T+\tilde{S}}\right\}P=e^{T}\left\{e^{\tilde{S}}\right\}P \end{array}$$

and the braces {} indicate that the normal ordering is imposed on the product of $\tilde{S}$ operators.

A specificity of the Fock space method lies in the hierarchical structure of the CC solutions. So, when we want to solve the Fock space equations for the given (k,l) sector, the lower rank sectors (i,j) solutions must be known. That is why in the current work, since we use the FS-CCSD (2,0) variant, the solutions for the (0,0) and (1,0) sectors are needed. The (0,0) sector corresponds to the single reference solution for the reference system (LiRb${}^{+2}$ ion), and the (1,0) sector is responsible for the electron affinity calculations, and the (2,0) sector is used for double electron affinity ones.

The particular sectors are defined by the configurations belonging to them. So, if the model space contains configurations $\Phi^{\alpha }$ with a single electron placed on the virtual level, α, we have the (1,0) sector; for configurations $\Phi^{\alpha \beta }$ with two additional electrons placed on the virtual levels, α, β, we have the (2,0) sector, etc. Note that the convention we adopted here indicates that the indices a,b,... refer to virtual one-particle levels, and their Greek equivalence α,β,... refer to active particles, while the $\bar{a},\bar{b},...$ refer to inactive particles. Thus, the DEA (double electron attached) states refer to the (2,0) sector. Consequently, only particle active levels belong to the active space and their number determines the size of the active space.

The energy values of the DEA states are obtained by the diagonalization of the effective Hamiltonian within the $\Phi^{\alpha \beta }$ configurational space:

(10) $$H_{eff}^{\left(2,0\right)}=P^{\left(2,0\right)}He^{S^{\left(0,0\right)}+S^{\left(1,0\right)}+S^{\left(2,0\right)}}P^{\left(2,0\right)}$$

where the projection operator $P^{\left(2,0\right)}$ is defined as

(11) $$P^{\left(2,0\right)}=\sum_{\alpha ,\beta }|\Phi^{\alpha \beta }\rangle \langle \Phi^{\alpha \beta }|$$

The cluster operators $S^{\left(0,0\right)}\left(\equiv T\right)$, $S^{\left(1,0\right)}$, and $S^{\left(2,0\right)}$ correspond to the sector indicated by the superscripts. As we mentioned before, in order to construct the effective Hamiltonian for the (2,0) sector $H_{eff}^{\left(2,0\right)}$, the amplitudes from the sectors (1,0) and (0,0) are needed, and the diagonalization of the $H_{eff}^{\left(2,0\right)}$ is performed only within the (2,0) sector. The $S^{\left(1,0\right)}$ operator can be constructed by solving the FS-MRCC equations for the (1,0) sector, but the identical result can be obtained by solving the EOM-CC problem set up for the electron affinity (EA). It is known that the Fock space approach at the one-valence level, (1,0) and (0,1), is equivalent to the EOM-CC scheme applied to the EA and IP (Ionization Potentials) cases, respectively. This equivalence means that the eigenvalues in both approaches are identical while the eigenvectors can be obtained from each other by a simple transformation.

A well-known problem that complicates the MR formulations of the coupled cluster theory is the presence of intruder states. They arise when the excited determinants from the orthogonal space (usually low in energy) are close to those belonging to the model space. This may cause numerical instabilities and, consequently, may be a reason for making the MRCC equations divergent.

In order to eliminate convergence problems in the (2,0) sector due to intruder states [70,71], we applied the intermediate Hamiltonian (IH) strategy [98,99,100]. Within IH formalism, we select a part of the orthogonal space, $M_{⊥}$ as an intermediate space, $M_{I}$, connected with the $P_{I}$ (projector onto the subspace defined by the operators $S^{\left(2,0\right)}$). Thus, in this formulation, we have the following subspaces: $M^{\left(2,0\right)},M_{I}^{\left(2,0\right)},M_{I⊥}^{\left(2,0\right)}$ and projectors: $P^{\left(2,0\right)}$, $P_{I}^{\left(2,0\right)}$, $Q_{0}^{\left(2,0\right)}$ with relations:

(12) $$P_{0}^{\left(2,0\right)}=P^{\left(2,0\right)}+P_{I}^{\left(2,0\right)}$$

(13) $$Q^{\left(2,0\right)}=P_{I}^{\left(2,0\right)}+Q_{0}^{\left(2,0\right)}$$

and wave operators,

(14) $$X^{\left(2,0\right)}=\left\{e^{{\tilde{S}}^{\left(2,0\right)}}-1\right\}P^{\left(2,0\right)}$$

(15) $$Z^{\left(2,0\right)}=P_{I}^{\left(2,0\right)}X^{\left(2,0\right)}P^{\left(2,0\right)}$$

(16) $$Y^{\left(2,0\right)}=Q_{0}^{\left(2,0\right)}X^{\left(2,0\right)}P^{\left(2,0\right)}$$

with $X^{\left(2,0\right)}=Z^{\left(2,0\right)}+Y^{\left(2,0\right)}$. $Z^{\left(2,0\right)}$, operating on the model space $P^{\left(2,0\right)}$, generates determinants belonging to the $M_{I}^{\left(2,0\right)}$ space, while the $Y^{\left(2,0\right)}$ operator connects the $P^{\left(2,0\right)}$ and $M_{I⊥}^{\left(2,0\right)}$ subspaces. For the IH-FS-CCSD case, they take the following form:

(17) $$\begin{array}{ccc} Z^{\left(2,0\right)} & = & P_{I}^{\left(2,0\right)}\left\{\left(S_{1}^{\left(1,0\right)}+S_{1}^{\left(1,0\right)}S_{1}^{\left(1,0\right)}+S_{2}^{\left(2,0\right)}\right)\right\}P^{\left(2,0\right)} \end{array}$$

(18) $$\begin{array}{ccc} Y^{\left(2,0\right)} & = & Q_{0}^{\left(2,0\right)}\left\{\left(S_{2}^{\left(1,0\right)}+S_{1}^{\left(1,0\right)}S_{2}^{\left(1,0\right)}+S_{2}^{\left(1,0\right)}S_{2}^{\left(1,0\right)}\right)\right\}P^{\left(2,0\right)} \end{array}$$

The eigenvalues of the effective Hamiltonian [99], defined within the FS-CC, are the same as those of the simpler intermediate Hamiltonian operator, i.e., $H_{I}^{\left(2,0\right)}$ [14]:

(19) $$\begin{array}{ccc} H_{I}^{\left(2,0\right)} & = & P_{0}^{\left(2,0\right)}\left\{e^{-Y^{\left(2,0\right)}}\right\}\bar{H}\left\{e^{Y^{\left(2,0\right)}}\right\}P_{0}^{\left(2,0\right)}=P_{0}^{\left(2,0\right)}\left(1-Y^{\left(2,0\right)}\right)\bar{H}\left(1+Y^{\left(2,0\right)}\right)P_{0}^{\left(2,0\right)} \end{array}$$

(20) $$\begin{array}{ccc} & = & P_{0}^{\left(2,0\right)}\bar{H}\left(1+Y^{\left(2,0\right)}\right)P_{0}^{\left(2,0\right)}=P_{0}^{\left(2,0\right)}\bar{H}P_{0}^{\left(2,0\right)}+P_{0}^{\left(2,0\right)}\bar{H}Y^{\left(2,0\right)}P^{\left(2,0\right)} \end{array}$$

This is a matrix representation of $\bar{H}$ in $M_{0}^{\left(2,0\right)}$ with the $P_{0}^{\left(2,0\right)}-P^{\left(2,0\right)}$ part modified by dressing, which is constructed from $\bar{H}$ and the cluster operators *S* known from the lower sector, i.e., (1,0) (for more details see the paper devoted to the IH-FS-CCSD (2,0) method [14]). The respective matrix to be diagonalized is obtained in the electron-affinity EOM-CC [103,104,105,106] calculations in order to omit convergence problems in the FS one-valence sector. Diagonalization of the $H_{I}^{\left(2,0\right)}$ operator provides a subset of eigenvalues that are identical to those obtained by diagonalization of the $H_{eff}^{\left(2,0\right)}$ operator. It follows from the above that the diagonalization of the IH matrix can replace the iterative solution of the $H_{eff}$-based equations. The final advantage of this is the elimination of the intruder state problem from the FS approach. In addition, the (2,0) step scales as $n^{5}$ (neglecting the limited number of active orbitals). The scaling of the CCSD solution for the ground state is $n^{6}$, and the (1,0) part is $n^{5}$.

## 4. Conclusions

The studies of the potential energy curves of diatomic molecules have a well-established place in the quantum chemical literature. The problem is challenging since upon a dissociation of the closed shell structure, usually the open shell fragments are formed, which complicates the calculations at the correlated level.

In this work, we focus on the Fock space realization of the multireference coupled cluster theory, specifically applied to investigate the electronic structure of the LiRb molecule. The central aspect of our approach lies in the Intermediate Hamiltonian formulation, providing a convenient way to circumvent the iterative solution inherent in the standard Bloch equation. Instead, we took advantage of the direct diagonalization of a suitably constructed matrix (’dressed’ $\bar{H}$, i.e., IH). This methodology eliminates complications associated with intruder states, a persistent hindrance that has restrained the broader adoption of this method over the years.

Promising findings were obtained in the studies of the dissociation of a single bond for LiRb with a solution based on the (2,0) sector of FS as an indirect application of the DEA calculations. Using this scheme, excellent results were obtained in the determination of the spectroscopic constants and PECs for the twenty-two lowest electronic states. Notably, calculations were performed for the first time across the entire range of interatomic distances using the RHF reference function, correlating all 40 electrons. This approach produced smooth and accurate PECs from the equilibrium distance to infinity. The mean absolute error from experimental $D_{e}$ is equal to 39 cm${}^{-1}$, while a corresponding value for $T_{e}$ was 46 cm${}^{-1}$. Additionally, good agreement with experimental data was observed for $\omega_{e}$, with an average deviation of 13 cm${}^{-1}$. Thus, the molecular data obtained in this study are well justified and, in the absence of experimental data, may be treated as reference values. This work presents the capability of the IH-FS-CCSD (2,0) method to provide comprehensive characteristics of the investigated molecule without relying on additional approximations.

## Figures and Tables

**Table 1:** Energies of electronic states at the dissociation limit of the LiRb molecule compared to the atomic energies in the unANO-RCC+ basis set.

Dissociation Limit	Li		Rb		Li + Rb	LiRb R = *∞*
	**Config.**	**E (a.u.)**	**Config.**	**E (a.u.)**	**E (a.u.)**	**E (a.u.)**
Li(2s) + Rb(5s)	[He] 2s	−7.473553	[Kr] 5s	−2939.440615	−2946.914167	−2946.914167
Li(2s) + Rb(5p)	[He] 2s	−7.473553	[Kr] 5p	−2939.385063	−2946.858616	−2946.858616
Li(2p) + Rb(5s)	[He] 2p	−7.405597	[Kr] 5s	−2939.440615	−2946.846212	−2946.846212
Li(2s) + Rb(4d)	[He] 2s	−7.473553	[Kr] 4d	−2939.355029	−2946.828582	−2946.828582
Li(2s) + Rb(6s)	[He] 2s	−7.473553	[Kr] 6s	−2939.350976	−2946.824529	−2946.824529
Li(2s) + Rb(6p)	[He] 2s	−7.473553	[Kr] 6p	−2939.334975	−2946.808528	−2946.808528

**Table 2:** The dipole moment of the $X^{1}\Sigma^{+}$ state of the LiRb molecule.

Sym.	μ (Debye)	Method
$X^{1}\Sigma^{+}$	4.01 (−0.38)	This work ${}^{a}$
	4.05	Exp. [81]
	4.1	Exp. [21]
	4.84	CCSD [22]
	4.42	MBPT4 [22]
	4.66	CCSD(T) [22]
	4.34	CCSD(T) + rel. [22]
	4.14	CCSD(T) [28]
	3.99	CCSD(T) [1]
	4.046	CCSDT [27]
	4.06	CCSDT [40]
	4.58	MRCI [41]
	4.168	Pseudopotential/basis A [7]
	4.142	Pseudopotential/basis B [7]
	4.13	Pseudopotential [31]
	4.78	Pseudopotential [39]
	4.46	Pseudopotential [34]
	4.165	Pseudopotential [23]
	4.78	Pseudopotential [38]
	4.40	DFT/PW91 [44]

${}^{a}$ Method used in this work, which is IH-FS-CCSD(2,0) DK3/unANO-RCC+ as described in the text. The contribution of the relativistic correction is given in the parenthesis.

**Table 3:** Energy barriers for the states with double minima. IH-FS-CCSD(2,0)/unANO-RCC+.

State	Position (Å)	Energy (a.u.)	Energy (cm${}^{-1}$)
$4^{3}\Sigma^{+}$			
Min.	4.18	−2946.834640	
Max.	4.75	−2946.833272	
Diff.		0.001368	300
$5^{1}\Sigma^{+}$			
Min.	4.02	−2946.839545	
Max.	8.05	−2946.828999	
Diff.		0.010546	2314
$6^{1}\Sigma^{+}$			
Min.	4.08	−2946.827538	
Max.	6.20	−2946.817448	
Diff.		0.010090	2214
$6^{3}\Sigma^{+}$			
Min.	4.40	−2946.824168	
Max.	8.00	−2946.810238	
Diff.		0.013930	3057

**Table 4:** Avoided crossing positions obtained using IH-FS-CCSD(2,0)/un-ANORCC+ compared with Ref. [38].

State	Position in This Work (Å)	Position in [38] (Å)
$3^{1}\Sigma^{+}$/ $4^{1}\Sigma^{+}$	8.61	N/A
$4^{1}\Sigma^{+}$/ $5^{1}\Sigma^{+}$	13.21	13.12
$5^{1}\Sigma^{+}$/ $6^{1}\Sigma^{+}$	14.24	N/A
$4^{3}\Sigma^{+}$/ $5^{3}\Sigma^{+}$	4.75	4.71
$3^{1}\Pi$ / $4^{1}\Pi$	3.26	3.12
	3.13	2.94
$3^{3}\Pi$ / $4^{3}\Pi$	3.88	3.84
	7.96	8.07

**Table 5:** Spectroscopic constants of LiRb. $D_{e}$, $T_{e}$, ω**_*e*_**, ω***_e_x_e_*** and $B_{e}$ given in ${\mathrm{cm}}^{-1}$; $R_{e}$ given in Å. DK3 relativistic corrections are given in parentheses.

Sym.	*D_e_*	*T_e_*	*R_e_*	ω _*e*_	ω *_e_x_e_*	*B_e_*	Source
Li(2s) + Rb(5s)							
$X^{1}\Sigma^{+}$	5886(−43)		3.466(−0.034)	194.53(2.60)	1.21(0.16)	0.216(0.004)	This work ${}^{a}$
	5968		3.428	196.02	1.44	0.223	[38] ${}^{b}$
	5917.0		3.490	195.3	1.31	0.216	[41] ${}^{c}$
	5922.5		3.508	194.0	1.240	0.213	[42] ${}^{d}$
	5921		3.466	195.18			Exp. [15]
$1^{3}\Sigma^{+}$	290(−15)	5592(−32)	4.993(0.014)	43.11(−0.93)	1.84(0.00)	0.104(−0.001)	This work ${}^{a}$
	276	5693	5.126	40.13	17.46	0.098	[38] ${}^{b}$
	282.4		5.141	39.1	0.85	0.096	[41] ${}^{c}$
	277.2	5650.5	5.140	40.548			Exp. [15]
Li(2s) + Rb(5s)							
$2^{1}\Sigma^{+}$	7003(170)	11,594(305)	4.166(−0.034)	120.03(2.70)	0.54(0.04)	0.150(0.002)	This work ${}^{a}$
	7053	11,654	4.137	118.78	1.04	0.153	[38] ${}^{b}$
	7039.6		4.201	116.5	0.56	0.147	[41] ${}^{c}$
		11,614	4.16				[43] ^e^
				117.3	0.36		Exp. [19]
$1^{1}\Pi$	1529(70)	17,069(407)	3.905(−0.057)	117.44(4.78)	3.05(0.23)	0.171(0.005)	This work ${}^{a}$
	1461	17,245	3.873	116.12	2.92	0.175	[38] ${}^{b}$
	1415.9	17,578.4	3.969	113.8	2.750	0.1651	[42] ${}^{d}$
	1634	17,110.406	3.8751	122.2			Exp. [16]
$2^{3}\Sigma^{+}$	3940(152)	14,657(323)	4.107(−0.020)	133.64(0.83)	1.42(−0.04)	0.154(0.001)	This work ${}^{a}$
	3969	14,737	4.058	128.63	1.09	0.159	[38] ${}^{b}$
	3997.2		4.133	128.7	0.97	0.152	[41] ${}^{c}$
$1^{3}\Pi$	8359(202)	10,237(273)	3.382(−0.016)	190.19(1.87)	0.74(0.08)	0.227(0.002)	This work ${}^{a}$
	8457	10,249	3.338	192.0	${}^{a}$0.860	0.235	[38] ${}^{b}$
		10,232	3.44				[43] ^e^
Li(2s) + Rb(5s)							
$3^{1}\Sigma^{+}$	3588(−155)	17,212(111)	4.228(0.021)	115.16(−3.35)	0.66(−0.05)	0.146(−0.001)	This work ${}^{a}$
	3494	17,382	4.243	114.24	1.22	0.145	[38] ${}^{b}$
	3601	17,230.571	4.2834	113.8			Exp. [16]
				115.4	0.36		Exp. [19]
$2^{1}\Pi$	1774(−145)	19,026(101)	4.091(0.017)	122.53(−2.10)	1.26(0.02)	0.156(−0.001)	This work ${}^{a}$
	1639	19,235	4.084	122.08	1.83	0.157	[38] ${}^{b}$
	1743	19,089.88	4.115	120.5			Exp. [16]
$3^{3}\Sigma^{+}$	409(−506)	20,390(462)	3.932(−0.022)	136.51(1.87)	1.68(0.09)	0.168(0.002)	This work ${}^{a}$
	362	20,513	3.904	136.61	1.82	0.171	[38] ${}^{b}$
$2^{3}\Pi$	1583(−476)	19,217(432)	4.128(−0.016)	104.46(−0.63)	1.61(0.23)	0.152(0.001)	This work ${}^{a}$
	1411	19,484	4.100	103.3	1.82	0.156	[38] ${}^{b}$
				195.1	0.84		Exp. [19]
Li(2s) + Rb(5s)							
$4^{1}\Sigma^{+}$	4090(200)	21,255(431)	7.635(0.380)	33.92(−1.00)	1.16(0.36)	0.045(−0.004)	This work ${}^{a}$
	3998	21,326	7.671	41.27	0.10	0.044	[38] ${}^{b}$
$3^{1}\Pi$	1190(66)	24,155(566)	4.573(−0.060)	82.82(2.90)	1.28(0.05)	0.124(0.003)	This work ${}^{a}$
	989	24,335	4.570	81	1.34	0.125	[38] ${}^{b}$
$1^{1}∆$	3209(156)	22,135(475)	3.709(−0.022)	142.39(2.26)	1.08(−0.04)	0.189(0.002)	This work ${}^{a}$
	3063	22,277	3.714	143.2	1.6	0.19	[38] ${}^{b}$
$4^{3}\Sigma^{+}$	1653(324)	23,692(308)	4.165(−0.016)	134.98(7.92)	8.91(2.78)	0.150(0.001)	This work ${}^{a}$
1st min.	1527	23,797	4.137	117.85	1.74	0.153	[38] ${}^{b}$
$4^{3}\Sigma^{+}$	2130(331)	23,215(301)	5.457(−0.193)	109.29(16.09)	2.81(1.06)	0.087(0.006)	This work ${}^{a}$
2nd min.	1983	23,340	5.433	109.58	1.74	0.088	[38] ${}^{b}$
				106.3	2.4		Exp. [18]
$3^{3}\Pi$	2172(361)	23,174(272)	5.651(−0.205)	70.32(4.62)	0.09(−0.03)	0.081(0.005)	This work ${}^{a}$
	2055	23,269	5.661	74.29	1.09	0.081	[38] ${}^{b}$
$1^{3}∆$	2413(145)	22,932(487)	3.932(−0.031)	135.37(3.51)	1.41(−0.04)	0.168(0.002)	This work ${}^{a}$
	2294	23,046	3.878	133.5	1.67	0.174	[38] ${}^{b}$
Li(2s) + Rb(5s)							
$5^{1}\Sigma^{+}$	3382(86)	22,583(276)	4.006(−0.018)	201.98(14.73)	4.86(0.48)	0.162(0.001)	This work ${}^{a}$
1st min.	3360	22,702	3.962	211.81	7.07	0.167	[38] ${}^{b}$
$5^{1}\Sigma^{+}$	480(−237)	25,483(598)	11.524(0.160)	12.65(−4.37)	0.70(0.24)	0.019(−0.001)	This work ${}^{a}$
2nd min.	598	25,473	11.650	13.10	7.07	0.019	[38] ${}^{b}$
$5^{3}\Sigma^{+}$	1935(46)	24,028(315)	4.724(−0.057)	210.11(−9.16)	12.40(−1.35)	0.117(0.003)	This work ${}^{a}$
	1964	24,106	4.740	215.11	43.55	0.116	[38] ${}^{b}$
Li(2s) + Rb(5s)							
$6^{1}\Sigma^{+}$	4471(184)	25,130(302)	4.055(−0.022)	132.59(1.01)	0.79(−0.07)	0.158(0.001)	This work ${}^{a}$
1st min.	4438	25,330	4.021	132.28	0.86	0.162	[38] ${}^{b}$
$6^{1}\Sigma^{+}$	3420(92)	26,179(392)	12.847(−0.128)	21.61(2.30)	−0.04(0.05)	0.016(0.001)	This work ${}^{a}$
2nd min.	3502	26,266	12.666	22.38	0.86	0.016	[38] ${}^{b}$
$4^{1}\Pi$	3902(136)	25,697(348)	3.968(−0.030)	129.77(1.99)	1.21(−0.02)	0.165(0.002)	This work ${}^{a}$
	3918	25,850	3.936	127.0	1.52	0.169	[38] ${}^{b}$
$6^{3}\Sigma^{+}$	3514(81)	26,085(403)	4.378(−0.032)	169.63(0.59)	3.14(−0.15)	0.136(0.002)	This work ${}^{a}$
1st min.	3598	26,170	4.343	175.31	6.02	0.139	[38] ${}^{b}$
$6^{3}\Sigma^{+}$	420(−11)	29,179(496)	9.264(0.054)	22.51(−0.04)	0.028(−0.01)	0.030(−0.001)	This work ${}^{a}$
2nd min.	444	29,324	9.560	19.35	6.02	0.028	[38] ${}^{b}$
$4^{3}\Pi$	4437(129)	25,162(355)	3.953(−0.038)	182.95(−2.55)	3.75(−0.73)	0.166(0.003)	This work ${}^{a}$
	4483	25,285	3.941	198.74	14.44	0.168	[38] ${}^{b}$
MAE${}^{f}$	39	46	0.051	13.46	0.42	−	This work ${}^{a}$
	86	118	0.025	13.24	0.80	−	[38] ${}^{b}$

*^a^* Method used in this work, which is IH-FS-CCSD(2,0) DK3/unANO-RCC+ as described in the text. *^b^* Method used in [38] was based on pseudopotentials. Basis set for lithium: (9s8p4d1f)/[8s6p3d1f], for rubidium: (7s5p5d2f)/[6s4p4d1f]. *^c^* Method used in [41] was based on the multireference configuration interaction method (MRCI). Basis set for lithium: aug-cc-pwCV5Z, for rubidium: ANO-type basis set. All-electron DKH relativistic formalism was used. *^d^* Method used in [42] was similar as in [41]. *^e^* Method used in [43] was Fock-space relativistic coupled-cluster with singles and doubles. Basis set for lithium: cc-pwCVQZ. For rubidium semilocal shape-consistent effective core potentials and [7s7p5d3f2g] basis set was used. *^f^* Mean absolute error calculated for the states where experimental results are available. For the 3^1^∑^+^ state the [16] is treated as a true value while for the *ω_e_x_e_* constant the [19] is treated as a true value.

## Data Availability

The data that support the findings of this study are available within the article and the supplementary material.

## Supplementary Materials

The following supporting information can be downloaded at: https://www.mdpi.com/article/10.3390/molecules28227645/s1, Figure S1: Potential energy curves of LiRb calculated using the IH-FS-CCSD(2,0)/unANO-RCC+ method for the six lowest dissociation limits. Energy related to the dissociation limit of the ground state.; Figure S2: Potential energy curves of LiRb calculated using the IH-FS-CCSD(2,0) DK3/Sapporo-DKH3-QZP-2012-diffuse method for the six lowest dissociation limits. Energy related to the dissociation limit of the ground state.; Table S1: Total IH-FS-CCSD (2,0)/unANO-RCC+ energy values (a.u.) for ${}^{1}\Sigma^{+}$ states of LiRb.; Table S2: Total IH-FS-CCSD (2,0)/unANO-RCC+ energy values (a.u.) for ${}^{3}\Sigma^{+}$ states of LiRb.; Table S3: Total IH-FS-CCSD (2,0)/unANO-RCC+ energy values (a.u.) for ${}^{1}\Pi$ and ${}^{1}∆$ states of LiRb.; Table S4: Total IH-FS-CCSD (2,0)/unANO-RCC+ energy values (a.u.) for ${}^{3}\Pi$ and ${}^{3}∆$ states of LiRb.; Table S5: Total IH-FS-CCSD (2,0) DK3/Sapporo-DKH3-QZP-2012-diffuse energy values (a.u.) for ${}^{1}\Sigma^{+}$ states of LiRb.; Table S6: Total IH-FS-CCSD (2,0) DK3/Sapporo-DKH3-QZP-2012-diffuse energy values (a.u.) for ${}^{3}\Sigma^{+}$ states of LiRb.; Table S7: Total IH-FS-CCSD (2,0) DK3/Sapporo-DKH3-QZP-2012-diffuse energy values (a.u.) for ${}^{1}\Pi$ and ${}^{1}∆$ states of LiRb.; Table S8: Total IH-FS-CCSD (2,0) DK3/Sapporo-DKH3-QZP-2012-diffuse energy values (a.u.) for ${}^{3}\Pi$ and ${}^{3}∆$ states of LiRb.; Table S9: Total IH-FS-CCSD (2,0)/unANO-RCC+ energy values (a.u.) (E + 2946.914167 a.u.) for ${}^{1}\Sigma^{+}$ states of LiRb.; Table S10: Total IH-FS-CCSD (2,0)/unANO-RCC+ energy values (a.u.) (E + 2946.914167 a.u.) for ${}^{3}\Sigma^{+}$ states of LiRb.; Table S11: Total IH-FS-CCSD (2,0)/unANO-RCC+ energy values (a.u.) (E + 2946.914167 a.u.) for ${}^{1}\Pi$ and ${}^{1}∆$ states of LiRb.; Table S12: Total IH-FS-CCSD (2,0)/unANO-RCC+ energy values (a.u.) (E + 2946.914167 a.u.) for ${}^{3}\Pi$ and ${}^{3}∆$ states of LiRb.; Table S13: Total IH-FS-CCSD (2,0) DK3/Sapporo-DKH3-QZP-2012-diffuse energy values (a.u.) (E + 2986.954313 a.u.) for ${}^{1}\Sigma^{+}$ states of LiRb.; Table S14: Total IH-FS-CCSD (2,0) DK3/Sapporo-DKH3-QZP-2012-diffuse energy values (a.u.) (E + 2986.954313 a.u.) for ${}^{3}\Sigma^{+}$ states of LiRb.; Table S15: Total IH-FS-CCSD (2,0) DK3/Sapporo-DKH3-QZP-2012-diffuse energy values (a.u.) (E + 2986.954313 a.u.) for ${}^{1}\Pi$ and ${}^{1}∆$ states of LiRb.; Table S16: Total IH-FS-CCSD (2,0) DK3/Sapporo-DKH3-QZP-2012-diffuse energy values (a.u.) (E + 2986.954313 a.u.) for ${}^{3}\Pi$ and ${}^{3}∆$ states of LiRb.
