# Supplementary material for: Benchmark Study of the Electronic States of the LiRb Molecule: Ab Initio Calculations with the Fock Space Coupled Cluster Approach
Source: Molecules. 2023 Nov 17;28(22):7645. doi: 10.3390/molecules28227645 (PMC10675596; doi:10.3390/molecules28227645)
Supplement: Supplementary file 1 [file molecules-28-07645-s001.zip › lirb_sapporo_pi_delta_singlet.pdf]

#R[A]	1^1 pi	R[A]	2^1 pi	R[A]	3^1 pi	R[A]	4^1 pi	R[A]	1^1 delta
1.4	-2986.552593	1.4	-2986.535773	1.4	-2986.504117	1.4	-2986.484872	1.4	-2986.531807
1.6	-2986.712371	1.6	-2986.695023	1.6	-2986.671452	1.6	-2986.646337	1.6	-2986.695971
1.8	-2986.789462	1.8	-2986.772278	1.8	-2986.751469	1.8	-2986.725918	1.8	-2986.775500
2.0	-2986.828856	2.0	-2986.813540	2.0	-2986.791705	2.0	-2986.769229	2.0	-2986.816129
2.2	-2986.850990	2.2	-2986.838150	2.2	-2986.813663	2.2	-2986.796245	2.2	-2986.838438
2.4	-2986.865536	2.4	-2986.854346	2.4	-2986.827324	2.4	-2986.815340	2.4	-2986.851956
2.6	-2986.876901	2.6	-2986.865736	2.6	-2986.837439	2.6	-2986.829650	2.6	-2986.861263
2.8	-2986.886079	2.8	-2986.874135	2.8	-2986.845689	2.8	-2986.840300	2.8	-2986.868163
3.0	-2986.893058	3.0	-2986.880557	3.0	-2986.852497	3.0	-2986.848103	3.0	-2986.873292
3.2	-2986.897958	3.2	-2986.885398	3.2	-2986.857888	3.2	-2986.853689	3.2	-2986.876877
3.4	-2986.901108	3.4	-2986.888861	3.4	-2986.862005	3.4	-2986.857461	3.4	-2986.879101
3.6	-2986.902891	3.6	-2986.891170	3.6	-2986.865081	3.6	-2986.859742	3.6	-2986.880193
3.8	-2986.903685	3.8	-2986.892519	3.8	-2986.867306	3.8	-2986.860874	3.8	-2986.880381
4.0	-2986.903826	4.0	-2986.893099	4.0	-2986.868849	4.0	-2986.861187	4.0	-2986.879912
4.2	-2986.903570	4.2	-2986.893073	4.2	-2986.869847	4.2	-2986.860945	4.2	-2986.878992
4.4	-2986.903110	4.4	-2986.892592	4.4	-2986.870417	4.4	-2986.860348	4.4	-2986.877792
4.6	-2986.902573	4.6	-2986.891800	4.6	-2986.870660	4.6	-2986.859544	4.6	-2986.876452
4.8	-2986.902031	4.8	-2986.890832	4.8	-2986.870663	4.8	-2986.858631	4.8	-2986.875081
5.0	-2986.901517	5.0	-2986.889806	5.0	-2986.870496	5.0	-2986.857664	5.0	-2986.873757
5.2	-2986.901043	5.2	-2986.888809	5.2	-2986.870216	5.2	-2986.856667	5.2	-2986.872533
5.4	-2986.900613	5.4	-2986.887900	5.4	-2986.869868	5.4	-2986.855645	5.4	-2986.871441
5.6	-2986.900224	5.6	-2986.887111	5.6	-2986.869491	5.6	-2986.854603	5.6	-2986.870492
5.8	-2986.899876	5.8	-2986.886453	5.8	-2986.869109	5.8	-2986.853556	5.8	-2986.869686
6.0	-2986.899561	6.0	-2986.885919	6.0	-2986.868745	6.0	-2986.852524	6.0	-2986.869012
6.2	-2986.899278	6.2	-2986.885495	6.2	-2986.868409	6.2	-2986.851535	6.2	-2986.868456
6.4	-2986.899024	6.4	-2986.885165	6.4	-2986.868106	6.4	-2986.850620	6.4	-2986.867999
6.6	-2986.898798	6.6	-2986.884915	6.6	-2986.867838	6.6	-2986.849802	6.6	-2986.867631
6.8	-2986.898595	6.8	-2986.884720	6.8	-2986.867603	6.8	-2986.849094	6.8	-2986.867323
7.0	-2986.898414	7.0	-2986.884571	7.0	-2986.867398	7.0	-2986.848498	7.0	-2986.867075
7.2	-2986.898252	7.2	-2986.884460	7.2	-2986.867219	7.2	-2986.848005	7.2	-2986.866873
7.4	-2986.898110	7.4	-2986.884374	7.4	-2986.867063	7.4	-2986.847604	7.4	-2986.866707
7.6	-2986.897985	7.6	-2986.884307	7.6	-2986.866928	7.6	-2986.847280	7.6	-2986.866571
7.8	-2986.897875	7.8	-2986.884256	7.8	-2986.866809	7.8	-2986.847018	7.8	-2986.866459
8.0	-2986.897779	8.0	-2986.884216	8.0	-2986.866705	8.0	-2986.846806	8.0	-2986.866367
8.2	-2986.897694	8.2	-2986.884185	8.2	-2986.866613	8.2	-2986.846634	8.2	-2986.866290
8.4	-2986.897621	8.4	-2986.884161	8.4	-2986.866532	8.4	-2986.846493	8.4	-2986.866221
8.6	-2986.897557	8.6	-2986.884142	8.6	-2986.866460	8.6	-2986.846377	8.6	-2986.866167
8.8	-2986.897501	8.8	-2986.884127	8.8	-2986.866395	8.8	-2986.846279	8.8	-2986.866123
9.0	-2986.897451	9.0	-2986.884114	9.0	-2986.866339	9.0	-2986.846197	9.0	-2986.866085
9.2	-2986.897409	9.2	-2986.884104	9.2	-2986.866288	9.2	-2986.846127	9.2	-2986.866053
9.4	-2986.897372	9.4	-2986.884096	9.4	-2986.866243	9.4	-2986.846067	9.4	-2986.866025
9.6	-2986.897339	9.6	-2986.884089	9.6	-2986.866202	9.6	-2986.846014	9.6	-2986.866002
9.8	-2986.897311	9.8	-2986.884083	9.8	-2986.866165	9.8	-2986.845968	9.8	-2986.865981
10.0	-2986.897286	10.0	-2986.884078	10.0	-2986.866132	10.0	-2986.845927	10.0	-2986.865964
10.2	-2986.897264	10.2	-2986.884073	10.2	-2986.866103	10.2	-2986.845891	10.2	-2986.865948
10.4	-2986.897244	10.4	-2986.884069	10.4	-2986.866076	10.4	-2986.845858	10.4	-2986.865935
10.6	-2986.897228	10.6	-2986.884066	10.6	-2986.866051	10.6	-2986.845828	10.6	-2986.865923
10.8	-2986.897212	10.8	-2986.884063	10.8	-2986.866029	10.8	-2986.845801	10.8	-2986.865913
11.0	-2986.897198	11.0	-2986.884060	11.0	-2986.865998	11.0	-2986.845776	11.0	-2986.865904
11.2	-2986.897186	11.2	-2986.884058	11.2	-2986.865982	11.2	-2986.845753	11.2	-2986.865896
11.4	-2986.897175	11.4	-2986.884056	11.4	-2986.865968	11.4	-2986.845732	11.4	-2986.865888
11.6	-2986.897165	11.6	-2986.884054	11.6	-2986.865955	11.6	-2986.845712	11.6	-2986.865881
11.8	-2986.897156	11.8	-2986.884052	11.8	-2986.865943	11.8	-2986.845695	11.8	-2986.865875
12.0	-2986.897149	12.0	-2986.884050	12.0	-2986.865933	12.0	-2986.845678	12.0	-2986.865870
12.2	-2986.897141	12.2	-2986.884049	12.2	-2986.865923	12.2	-2986.845663	12.2	-2986.865865
12.4	-2986.897135	12.4	-2986.884047	12.4	-2986.865915	12.4	-2986.845649	12.4	-2986.865860
12.6	-2986.897129	12.6	-2986.884046	12.6	-2986.865907	12.6	-2986.845636	12.6	-2986.865857
12.8	-2986.897123	12.8	-2986.884045	12.8	-2986.865901	12.8	-2986.845624	12.8	-2986.865853
13.0	-2986.897119	13.0	-2986.884044	13.0	-2986.865894	13.0	-2986.845613	13.0	-2986.865850
13.2	-2986.897115	13.2	-2986.884043	13.2	-2986.865888	13.2	-2986.845604	13.2	-2986.865847
13.4	-2986.897111	13.4	-2986.884042	13.4	-2986.865883	13.4	-2986.845595	13.4	-2986.865844
13.6	-2986.897107	13.6	-2986.884042	13.6	-2986.865878	13.6	-2986.845586	13.6	-2986.865842
13.8	-2986.897104	13.8	-2986.884041	13.8	-2986.865873	13.8	-2986.845579	13.8	-2986.865840
14.0	-2986.897101	14.0	-2986.884041	14.0	-2986.865869	14.0	-2986.845572	14.0	-2986.865838
14.2	-2986.897099	14.2	-2986.884040	14.2	-2986.865865	14.2	-2986.845566	14.2	-2986.865836
14.4	-2986.897097	14.4	-2986.884040	14.4	-2986.865860	14.4	-2986.845560	14.4	-2986.865835
14.6	-2986.897094	14.6	-2986.884039	14.6	-2986.865857	14.6	-2986.845555	14.6	-2986.865833
14.8	-2986.897092	14.8	-2986.884039	14.8	-2986.865854	14.8	-2986.845550	14.8	-2986.865832
15.0	-2986.897091	15.0	-2986.884039	15.0	-2986.865852	15.0	-2986.845546	15.0	-2986.865831
16.0	-2986.897084	16.0	-2986.884038	16.0	-2986.865841	16.0	-2986.845530	16.0	-2986.865827
18.0	-2986.897077	18.0	-2986.884037	18.0	-2986.865831	18.0	-2986.845514	18.0	-2986.865823
20.0	-2986.897074	20.0	-2986.884037	20.0	-2986.865827	20.0	-2986.845508	20.0	-2986.865822
30.0	-2986.897071	30.0	-2986.884037	30.0	-2986.865823	30.0	-2986.845504	30.0	-2986.865821
100.0	-2986.897071	100.0	-2986.884037	100.0	-2986.865822	100.0	-2986.845503	100.0	-2986.865822
200.0	-2986.897071	200.0	-2986.884037	200.0	-2986.865822	200.0	-2986.845503	200.0	-2986.865822
