# Supplementary material for: Benchmark Study of the Electronic States of the LiRb Molecule: Ab Initio Calculations with the Fock Space Coupled Cluster Approach
Source: Molecules. 2023 Nov 17;28(22):7645. doi: 10.3390/molecules28227645 (PMC10675596; doi:10.3390/molecules28227645)
Supplement: Supplementary file 1 [file molecules-28-07645-s001.zip › lirb_sapporo_pi_delta_singlet_asymptotic.pdf]

#R[A]	1^1 pi	R[A]	2^1 pi	R[A]	3^1 pi	R[A]	4^1 pi	R[A]	1^1 delta
1.4	0.401720	1.4	0.418540	1.4	0.450196	1.4	0.469441	1.4	0.422506
1.6	0.241942	1.6	0.259290	1.6	0.282861	1.6	0.307976	1.6	0.258342
1.8	0.164851	1.8	0.182035	1.8	0.202844	1.8	0.228395	1.8	0.178813
2.0	0.125457	2.0	0.140773	2.0	0.162608	2.0	0.185084	2.0	0.138184
2.2	0.103323	2.2	0.116163	2.2	0.140650	2.2	0.158068	2.2	0.115875
2.4	0.088777	2.4	0.099967	2.4	0.126989	2.4	0.138973	2.4	0.102357
2.6	0.077412	2.6	0.088577	2.6	0.116874	2.6	0.124663	2.6	0.093050
2.8	0.068234	2.8	0.080178	2.8	0.108624	2.8	0.114013	2.8	0.086150
3.0	0.061255	3.0	0.073756	3.0	0.101816	3.0	0.106210	3.0	0.081021
3.2	0.056355	3.2	0.068915	3.2	0.096425	3.2	0.100624	3.2	0.077436
3.4	0.053205	3.4	0.065452	3.4	0.092308	3.4	0.096852	3.4	0.075212
3.6	0.051422	3.6	0.063143	3.6	0.089232	3.6	0.094571	3.6	0.074120
3.8	0.050628	3.8	0.061794	3.8	0.087007	3.8	0.093439	3.8	0.073932
4.0	0.050487	4.0	0.061214	4.0	0.085464	4.0	0.093126	4.0	0.074401
4.2	0.050743	4.2	0.061240	4.2	0.084466	4.2	0.093368	4.2	0.075321
4.4	0.051203	4.4	0.061721	4.4	0.083896	4.4	0.093965	4.4	0.076521
4.6	0.051740	4.6	0.062513	4.6	0.083653	4.6	0.094769	4.6	0.077861
4.8	0.052282	4.8	0.063481	4.8	0.083650	4.8	0.095682	4.8	0.079232
5.0	0.052796	5.0	0.064507	5.0	0.083817	5.0	0.096649	5.0	0.080556
5.2	0.053270	5.2	0.065504	5.2	0.084097	5.2	0.097646	5.2	0.081780
5.4	0.053700	5.4	0.066413	5.4	0.084445	5.4	0.098668	5.4	0.082872
5.6	0.054089	5.6	0.067202	5.6	0.084822	5.6	0.099710	5.6	0.083821
5.8	0.054437	5.8	0.067860	5.8	0.085204	5.8	0.100757	5.8	0.084627
6.0	0.054752	6.0	0.068394	6.0	0.085568	6.0	0.101789	6.0	0.085301
6.2	0.055035	6.2	0.068818	6.2	0.085904	6.2	0.102778	6.2	0.085857
6.4	0.055289	6.4	0.069148	6.4	0.086207	6.4	0.103693	6.4	0.086314
6.6	0.055515	6.6	0.069398	6.6	0.086475	6.6	0.104511	6.6	0.086682
6.8	0.055718	6.8	0.069593	6.8	0.086710	6.8	0.105219	6.8	0.086990
7.0	0.055899	7.0	0.069742	7.0	0.086915	7.0	0.105815	7.0	0.087238
7.2	0.056061	7.2	0.069853	7.2	0.087094	7.2	0.106308	7.2	0.087440
7.4	0.056203	7.4	0.069939	7.4	0.087250	7.4	0.106709	7.4	0.087606
7.6	0.056328	7.6	0.070006	7.6	0.087385	7.6	0.107033	7.6	0.087742
7.8	0.056438	7.8	0.070057	7.8	0.087504	7.8	0.107295	7.8	0.087854
8.0	0.056534	8.0	0.070097	8.0	0.087608	8.0	0.107507	8.0	0.087946
8.2	0.056619	8.2	0.070128	8.2	0.087700	8.2	0.107679	8.2	0.088023
8.4	0.056692	8.4	0.070152	8.4	0.087781	8.4	0.107820	8.4	0.088092
8.6	0.056756	8.6	0.070171	8.6	0.087853	8.6	0.107936	8.6	0.088146
8.8	0.056812	8.8	0.070186	8.8	0.087918	8.8	0.108034	8.8	0.088190
9.0	0.056862	9.0	0.070199	9.0	0.087974	9.0	0.108116	9.0	0.088228
9.2	0.056904	9.2	0.070209	9.2	0.088025	9.2	0.108186	9.2	0.088260
9.4	0.056941	9.4	0.070217	9.4	0.088070	9.4	0.108246	9.4	0.088288
9.6	0.056974	9.6	0.070224	9.6	0.088111	9.6	0.108299	9.6	0.088311
9.8	0.057002	9.8	0.070230	9.8	0.088148	9.8	0.108345	9.8	0.088332
10.0	0.057027	10.0	0.070235	10.0	0.088181	10.0	0.108386	10.0	0.088349
10.2	0.057049	10.2	0.070240	10.2	0.088210	10.2	0.108422	10.2	0.088365
10.4	0.057069	10.4	0.070244	10.4	0.088237	10.4	0.108455	10.4	0.088378
10.6	0.057085	10.6	0.070247	10.6	0.088262	10.6	0.108485	10.6	0.088390
10.8	0.057101	10.8	0.070250	10.8	0.088284	10.8	0.108512	10.8	0.088400
11.0	0.057115	11.0	0.070253	11.0	0.088315	11.0	0.108537	11.0	0.088409
11.2	0.057127	11.2	0.070255	11.2	0.088331	11.2	0.108560	11.2	0.088417
11.4	0.057138	11.4	0.070257	11.4	0.088345	11.4	0.108581	11.4	0.088425
11.6	0.057148	11.6	0.070259	11.6	0.088358	11.6	0.108601	11.6	0.088432
11.8	0.057157	11.8	0.070261	11.8	0.088370	11.8	0.108618	11.8	0.088438
12.0	0.057164	12.0	0.070263	12.0	0.088380	12.0	0.108635	12.0	0.088443
12.2	0.057172	12.2	0.070264	12.2	0.088390	12.2	0.108650	12.2	0.088448
12.4	0.057178	12.4	0.070266	12.4	0.088398	12.4	0.108664	12.4	0.088453
12.6	0.057184	12.6	0.070267	12.6	0.088406	12.6	0.108677	12.6	0.088456
12.8	0.057190	12.8	0.070268	12.8	0.088412	12.8	0.108689	12.8	0.088460
13.0	0.057194	13.0	0.070269	13.0	0.088419	13.0	0.108700	13.0	0.088463
13.2	0.057198	13.2	0.070270	13.2	0.088425	13.2	0.108709	13.2	0.088466
13.4	0.057202	13.4	0.070271	13.4	0.088430	13.4	0.108718	13.4	0.088469
13.6	0.057206	13.6	0.070271	13.6	0.088435	13.6	0.108727	13.6	0.088471
13.8	0.057209	13.8	0.070272	13.8	0.088440	13.8	0.108734	13.8	0.088473
14.0	0.057212	14.0	0.070272	14.0	0.088444	14.0	0.108741	14.0	0.088475
14.2	0.057214	14.2	0.070273	14.2	0.088448	14.2	0.108747	14.2	0.088477
14.4	0.057216	14.4	0.070273	14.4	0.088453	14.4	0.108753	14.4	0.088478
14.6	0.057219	14.6	0.070274	14.6	0.088456	14.6	0.108758	14.6	0.088480
14.8	0.057221	14.8	0.070274	14.8	0.088459	14.8	0.108763	14.8	0.088481
15.0	0.057222	15.0	0.070274	15.0	0.088461	15.0	0.108767	15.0	0.088482
16.0	0.057229	16.0	0.070275	16.0	0.088472	16.0	0.108783	16.0	0.088486
18.0	0.057236	18.0	0.070276	18.0	0.088482	18.0	0.108799	18.0	0.088490
20.0	0.057239	20.0	0.070276	20.0	0.088486	20.0	0.108805	20.0	0.088491
30.0	0.057242	30.0	0.070276	30.0	0.088490	30.0	0.108809	30.0	0.088492
100.0	0.057242	100.0	0.070276	100.0	0.088491	100.0	0.108810	100.0	0.088491
200.0	0.057242	200.0	0.070276	200.0	0.088491	200.0	0.108810	200.0	0.088491
