# Supplementary material for: Benchmark Study of the Electronic States of the LiRb Molecule: Ab Initio Calculations with the Fock Space Coupled Cluster Approach
Source: Molecules. 2023 Nov 17;28(22):7645. doi: 10.3390/molecules28227645 (PMC10675596; doi:10.3390/molecules28227645)
Supplement: Supplementary file 1 [file molecules-28-07645-s001.zip › lirb_sapporo_pi_delta_triplet.pdf]

#R[A]	1^3 pi	R[A]	2^3 pi	R[A]	3^3 pi	R[A]	4^3 pi	R[A]	1^3 delta
1.4	-2986.591107	1.4	-2986.544444	1.4	-2986.506933	1.4	-2986.483744	1.4	-2986.523682
1.6	-2986.752436	1.6	-2986.700160	1.6	-2986.675211	1.6	-2986.644303	1.6	-2986.684267
1.8	-2986.830660	1.8	-2986.774486	1.8	-2986.756383	1.8	-2986.723721	1.8	-2986.764551
2.0	-2986.871535	2.0	-2986.812910	2.0	-2986.797720	2.0	-2986.767445	2.0	-2986.804285
2.2	-2986.895007	2.2	-2986.835313	2.2	-2986.820207	2.2	-2986.795163	2.2	-2986.824647
2.4	-2986.909780	2.4	-2986.850633	2.4	-2986.833418	2.4	-2986.815182	2.4	-2986.836991
2.6	-2986.919845	2.6	-2986.862576	2.6	-2986.842090	2.6	-2986.830577	2.6	-2986.849320
2.8	-2986.926631	2.8	-2986.872081	2.8	-2986.848420	2.8	-2986.842205	2.8	-2986.858884
3.0	-2986.930903	3.0	-2986.879414	3.0	-2986.853653	3.0	-2986.850401	3.0	-2986.866027
3.2	-2986.933166	3.2	-2986.884794	3.2	-2986.858275	3.2	-2986.855674	3.2	-2986.871037
3.4	-2986.933846	3.4	-2986.888525	3.4	-2986.861724	3.4	-2986.859345	3.4	-2986.874267
3.6	-2986.933304	3.6	-2986.890936	3.6	-2986.863879	3.6	-2986.862155	3.6	-2986.876050
3.8	-2986.931840	3.8	-2986.892349	3.8	-2986.865506	3.8	-2986.863813	3.8	-2986.876814
4.0	-2986.929720	4.0	-2986.893025	4.0	-2986.867324	4.0	-2986.863997	4.0	-2986.876821
4.2	-2986.927166	4.2	-2986.893170	4.2	-2986.868983	4.2	-2986.863382	4.2	-2986.876315
4.4	-2986.924354	4.4	-2986.892957	4.4	-2986.870385	4.4	-2986.862345	4.4	-2986.875488
4.6	-2986.921423	4.6	-2986.892518	4.6	-2986.871544	4.6	-2986.861114	4.6	-2986.874492
4.8	-2986.918483	4.8	-2986.891949	4.8	-2986.872485	4.8	-2986.859880	4.8	-2986.873437
5.0	-2986.915620	5.0	-2986.891313	5.0	-2986.873233	5.0	-2986.858818	5.0	-2986.872403
5.2	-2986.912904	5.2	-2986.890647	5.2	-2986.873808	5.2	-2986.858035	5.2	-2986.871438
5.4	-2986.910391	5.4	-2986.889971	5.4	-2986.874221	5.4	-2986.857523	5.4	-2986.870571
5.6	-2986.908122	5.6	-2986.889291	5.6	-2986.874481	5.6	-2986.857216	5.6	-2986.869812
5.8	-2986.906126	5.8	-2986.888612	5.8	-2986.874592	5.8	-2986.857049	5.8	-2986.869162
6.0	-2986.904416	6.0	-2986.887941	6.0	-2986.874553	6.0	-2986.856976	6.0	-2986.868613
6.2	-2986.902986	6.2	-2986.887293	6.2	-2986.874363	6.2	-2986.856953	6.2	-2986.868155
6.4	-2986.901818	6.4	-2986.886689	6.4	-2986.874023	6.4	-2986.856945	6.4	-2986.867774
6.6	-2986.900880	6.6	-2986.886151	6.6	-2986.873550	6.6	-2986.856927	6.6	-2986.867463
6.8	-2986.900135	6.8	-2986.885693	6.8	-2986.872958	6.8	-2986.856873	6.8	-2986.867198
7.0	-2986.899547	7.0	-2986.885317	7.0	-2986.872285	7.0	-2986.856770	7.0	-2986.866983
7.2	-2986.899084	7.2	-2986.885021	7.2	-2986.871566	7.2	-2986.856604	7.2	-2986.866804
7.4	-2986.898719	7.4	-2986.884790	7.4	-2986.870843	7.4	-2986.856366	7.4	-2986.866657
7.6	-2986.898430	7.6	-2986.884614	7.6	-2986.870146	7.6	-2986.856051	7.6	-2986.866534
7.8	-2986.898200	7.8	-2986.884481	7.8	-2986.869499	7.8	-2986.855655	7.8	-2986.866432
8.0	-2986.898016	8.0	-2986.884380	8.0	-2986.868918	8.0	-2986.855181	8.0	-2986.866347
8.2	-2986.897868	8.2	-2986.884305	8.2	-2986.868409	8.2	-2986.854636	8.2	-2986.866276
8.4	-2986.897747	8.4	-2986.884248	8.4	-2986.867975	8.4	-2986.854029	8.4	-2986.866210
8.6	-2986.897649	8.6	-2986.884205	8.6	-2986.867609	8.6	-2986.853373	8.6	-2986.866160
8.8	-2986.897569	8.8	-2986.884173	8.8	-2986.867305	8.8	-2986.852682	8.8	-2986.866117
9.0	-2986.897501	9.0	-2986.884148	9.0	-2986.867056	9.0	-2986.851971	9.0	-2986.866080
9.2	-2986.897445	9.2	-2986.884128	9.2	-2986.866851	9.2	-2986.851252	9.2	-2986.866050
9.4	-2986.897398	9.4	-2986.884113	9.4	-2986.866684	9.4	-2986.850541	9.4	-2986.866023
9.6	-2986.897358	9.6	-2986.884102	9.6	-2986.866547	9.6	-2986.849852	9.6	-2986.866000
9.8	-2986.897325	9.8	-2986.884092	9.8	-2986.866435	9.8	-2986.849199	9.8	-2986.865980
10.0	-2986.897296	10.0	-2986.884084	10.0	-2986.866343	10.0	-2986.848597	10.0	-2986.865963
10.2	-2986.897271	10.2	-2986.884078	10.2	-2986.866267	10.2	-2986.848058	10.2	-2986.865948
10.4	-2986.897250	10.4	-2986.884073	10.4	-2986.866204	10.4	-2986.847590	10.4	-2986.865934
10.6	-2986.897232	10.6	-2986.884069	10.6	-2986.866151	10.6	-2986.847198	10.6	-2986.865923
10.8	-2986.897215	10.8	-2986.884065	10.8	-2986.866107	10.8	-2986.846877	10.8	-2986.865912
11.0	-2986.897200	11.0	-2986.884062	11.0	-2986.866059	11.0	-2986.846621	11.0	-2986.865904
11.2	-2986.897188	11.2	-2986.884059	11.2	-2986.866029	11.2	-2986.846415	11.2	-2986.865896
11.4	-2986.897176	11.4	-2986.884056	11.4	-2986.866004	11.4	-2986.846252	11.4	-2986.865888
11.6	-2986.897166	11.6	-2986.884054	11.6	-2986.865983	11.6	-2986.846122	11.6	-2986.865881
11.8	-2986.897157	11.8	-2986.884052	11.8	-2986.865965	11.8	-2986.846018	11.8	-2986.865875
12.0	-2986.897149	12.0	-2986.884050	12.0	-2986.865950	12.0	-2986.845935	12.0	-2986.865870
12.2	-2986.897142	12.2	-2986.884049	12.2	-2986.865936	12.2	-2986.845867	12.2	-2986.865865
12.4	-2986.897135	12.4	-2986.884047	12.4	-2986.865925	12.4	-2986.845812	12.4	-2986.865860
12.6	-2986.897129	12.6	-2986.884046	12.6	-2986.865915	12.6	-2986.845766	12.6	-2986.865856
12.8	-2986.897124	12.8	-2986.884045	12.8	-2986.865907	12.8	-2986.845728	12.8	-2986.865853
13.0	-2986.897119	13.0	-2986.884044	13.0	-2986.865899	13.0	-2986.845696	13.0	-2986.865850
13.2	-2986.897115	13.2	-2986.884043	13.2	-2986.865892	13.2	-2986.845670	13.2	-2986.865847
13.4	-2986.897111	13.4	-2986.884042	13.4	-2986.865885	13.4	-2986.845648	13.4	-2986.865844
13.6	-2986.897107	13.6	-2986.884042	13.6	-2986.865880	13.6	-2986.845629	13.6	-2986.865842
13.8	-2986.897104	13.8	-2986.884041	13.8	-2986.865875	13.8	-2986.845613	13.8	-2986.865840
14.0	-2986.897101	14.0	-2986.884041	14.0	-2986.865870	14.0	-2986.845599	14.0	-2986.865838
14.2	-2986.897099	14.2	-2986.884040	14.2	-2986.865866	14.2	-2986.845587	14.2	-2986.865836
14.4	-2986.897097	14.4	-2986.884040	14.4	-2986.865861	14.4	-2986.845577	14.4	-2986.865835
14.6	-2986.897094	14.6	-2986.884039	14.6	-2986.865858	14.6	-2986.845569	14.6	-2986.865833
14.8	-2986.897092	14.8	-2986.884039	14.8	-2986.865855	14.8	-2986.845561	14.8	-2986.865832
15.0	-2986.897091	15.0	-2986.884039	15.0	-2986.865852	15.0	-2986.845555	15.0	-2986.865831
16.0	-2986.897084	16.0	-2986.884038	16.0	-2986.865842	16.0	-2986.845532	16.0	-2986.865827
18.0	-2986.897077	18.0	-2986.884037	18.0	-2986.865831	18.0	-2986.845514	18.0	-2986.865823
20.0	-2986.897074	20.0	-2986.884037	20.0	-2986.865827	20.0	-2986.845508	20.0	-2986.865822
30.0	-2986.897071	30.0	-2986.884037	30.0	-2986.865823	30.0	-2986.845504	30.0	-2986.865821
100.0	-2986.897071	100.0	-2986.884037	100.0	-2986.865822	100.0	-2986.845503	100.0	-2986.865822
200.0	-2986.897071	200.0	-2986.884037	200.0	-2986.865822	200.0	-2986.845503	200.0	-2986.865822
