# Supplementary material for: Benchmark Study of the Electronic States of the LiRb Molecule: Ab Initio Calculations with the Fock Space Coupled Cluster Approach
Source: Molecules. 2023 Nov 17;28(22):7645. doi: 10.3390/molecules28227645 (PMC10675596; doi:10.3390/molecules28227645)
Supplement: Supplementary file 1 [file molecules-28-07645-s001.zip › lirb_sapporo_pi_delta_triplet_asymptotic.pdf]

#R[A]	1^3 pi	R[A]	2^3 pi	R[A]	3^3 pi	R[A]	4^3 pi	R[A]	1^3 delta
1.4	0.363206	1.4	0.409869	1.4	0.447380	1.4	0.470569	1.4	0.430631
1.6	0.201877	1.6	0.254153	1.6	0.279102	1.6	0.310010	1.6	0.270046
1.8	0.123653	1.8	0.179827	1.8	0.197930	1.8	0.230592	1.8	0.189762
2.0	0.082778	2.0	0.141403	2.0	0.156593	2.0	0.186868	2.0	0.150028
2.2	0.059306	2.2	0.119000	2.2	0.134106	2.2	0.159150	2.2	0.129666
2.4	0.044533	2.4	0.103680	2.4	0.120895	2.4	0.139131	2.4	0.117322
2.6	0.034468	2.6	0.091737	2.6	0.112223	2.6	0.123736	2.6	0.104993
2.8	0.027682	2.8	0.082232	2.8	0.105893	2.8	0.112108	2.8	0.095429
3.0	0.023410	3.0	0.074899	3.0	0.100660	3.0	0.103912	3.0	0.088286
3.2	0.021147	3.2	0.069519	3.2	0.096038	3.2	0.098639	3.2	0.083276
3.4	0.020467	3.4	0.065788	3.4	0.092589	3.4	0.094968	3.4	0.080046
3.6	0.021009	3.6	0.063377	3.6	0.090434	3.6	0.092158	3.6	0.078263
3.8	0.022473	3.8	0.061964	3.8	0.088807	3.8	0.090500	3.8	0.077499
4.0	0.024593	4.0	0.061288	4.0	0.086989	4.0	0.090316	4.0	0.077492
4.2	0.027147	4.2	0.061143	4.2	0.085330	4.2	0.090931	4.2	0.077998
4.4	0.029959	4.4	0.061356	4.4	0.083928	4.4	0.091968	4.4	0.078825
4.6	0.032890	4.6	0.061795	4.6	0.082769	4.6	0.093199	4.6	0.079821
4.8	0.035830	4.8	0.062364	4.8	0.081828	4.8	0.094433	4.8	0.080876
5.0	0.038693	5.0	0.063000	5.0	0.081080	5.0	0.095495	5.0	0.081910
5.2	0.041409	5.2	0.063666	5.2	0.080505	5.2	0.096278	5.2	0.082875
5.4	0.043922	5.4	0.064342	5.4	0.080092	5.4	0.096790	5.4	0.083742
5.6	0.046191	5.6	0.065022	5.6	0.079832	5.6	0.097097	5.6	0.084501
5.8	0.048187	5.8	0.065701	5.8	0.079721	5.8	0.097264	5.8	0.085151
6.0	0.049897	6.0	0.066372	6.0	0.079760	6.0	0.097337	6.0	0.085700
6.2	0.051327	6.2	0.067020	6.2	0.079950	6.2	0.097360	6.2	0.086158
6.4	0.052495	6.4	0.067624	6.4	0.080290	6.4	0.097368	6.4	0.086539
6.6	0.053433	6.6	0.068162	6.6	0.080763	6.6	0.097386	6.6	0.086850
6.8	0.054178	6.8	0.068620	6.8	0.081355	6.8	0.097440	6.8	0.087115
7.0	0.054766	7.0	0.068996	7.0	0.082028	7.0	0.097543	7.0	0.087330
7.2	0.055229	7.2	0.069292	7.2	0.082747	7.2	0.097709	7.2	0.087509
7.4	0.055594	7.4	0.069523	7.4	0.083470	7.4	0.097947	7.4	0.087656
7.6	0.055883	7.6	0.069699	7.6	0.084167	7.6	0.098262	7.6	0.087779
7.8	0.056113	7.8	0.069832	7.8	0.084814	7.8	0.098658	7.8	0.087881
8.0	0.056297	8.0	0.069933	8.0	0.085395	8.0	0.099132	8.0	0.087966
8.2	0.056445	8.2	0.070008	8.2	0.085904	8.2	0.099677	8.2	0.088037
8.4	0.056566	8.4	0.070065	8.4	0.086338	8.4	0.100284	8.4	0.088103
8.6	0.056664	8.6	0.070108	8.6	0.086704	8.6	0.100940	8.6	0.088153
8.8	0.056744	8.8	0.070140	8.8	0.087008	8.8	0.101631	8.8	0.088196
9.0	0.056812	9.0	0.070165	9.0	0.087257	9.0	0.102342	9.0	0.088233
9.2	0.056868	9.2	0.070185	9.2	0.087462	9.2	0.103061	9.2	0.088263
9.4	0.056915	9.4	0.070200	9.4	0.087629	9.4	0.103772	9.4	0.088290
9.6	0.056955	9.6	0.070211	9.6	0.087766	9.6	0.104461	9.6	0.088313
9.8	0.056988	9.8	0.070221	9.8	0.087878	9.8	0.105114	9.8	0.088333
10.0	0.057017	10.0	0.070229	10.0	0.087970	10.0	0.105716	10.0	0.088350
10.2	0.057042	10.2	0.070235	10.2	0.088046	10.2	0.106255	10.2	0.088365
10.4	0.057063	10.4	0.070240	10.4	0.088109	10.4	0.106723	10.4	0.088379
10.6	0.057081	10.6	0.070244	10.6	0.088162	10.6	0.107115	10.6	0.088390
10.8	0.057098	10.8	0.070248	10.8	0.088206	10.8	0.107436	10.8	0.088401
11.0	0.057113	11.0	0.070251	11.0	0.088254	11.0	0.107692	11.0	0.088409
11.2	0.057125	11.2	0.070254	11.2	0.088284	11.2	0.107898	11.2	0.088417
11.4	0.057137	11.4	0.070257	11.4	0.088309	11.4	0.108061	11.4	0.088425
11.6	0.057147	11.6	0.070259	11.6	0.088330	11.6	0.108191	11.6	0.088432
11.8	0.057156	11.8	0.070261	11.8	0.088348	11.8	0.108295	11.8	0.088438
12.0	0.057164	12.0	0.070263	12.0	0.088363	12.0	0.108378	12.0	0.088443
12.2	0.057171	12.2	0.070264	12.2	0.088377	12.2	0.108446	12.2	0.088448
12.4	0.057178	12.4	0.070266	12.4	0.088388	12.4	0.108501	12.4	0.088453
12.6	0.057184	12.6	0.070267	12.6	0.088398	12.6	0.108547	12.6	0.088457
12.8	0.057189	12.8	0.070268	12.8	0.088406	12.8	0.108585	12.8	0.088460
13.0	0.057194	13.0	0.070269	13.0	0.088414	13.0	0.108617	13.0	0.088463
13.2	0.057198	13.2	0.070270	13.2	0.088421	13.2	0.108643	13.2	0.088466
13.4	0.057202	13.4	0.070271	13.4	0.088428	13.4	0.108665	13.4	0.088469
13.6	0.057206	13.6	0.070271	13.6	0.088433	13.6	0.108684	13.6	0.088471
13.8	0.057209	13.8	0.070272	13.8	0.088438	13.8	0.108700	13.8	0.088473
14.0	0.057212	14.0	0.070272	14.0	0.088443	14.0	0.108714	14.0	0.088475
14.2	0.057214	14.2	0.070273	14.2	0.088447	14.2	0.108726	14.2	0.088477
14.4	0.057216	14.4	0.070273	14.4	0.088452	14.4	0.108736	14.4	0.088478
14.6	0.057219	14.6	0.070274	14.6	0.088455	14.6	0.108744	14.6	0.088480
14.8	0.057221	14.8	0.070274	14.8	0.088458	14.8	0.108752	14.8	0.088481
15.0	0.057222	15.0	0.070274	15.0	0.088461	15.0	0.108758	15.0	0.088482
16.0	0.057229	16.0	0.070275	16.0	0.088471	16.0	0.108781	16.0	0.088486
18.0	0.057236	18.0	0.070276	18.0	0.088482	18.0	0.108799	18.0	0.088490
20.0	0.057239	20.0	0.070276	20.0	0.088486	20.0	0.108805	20.0	0.088491
30.0	0.057242	30.0	0.070276	30.0	0.088490	30.0	0.108809	30.0	0.088492
100.0	0.057242	100.0	0.070276	100.0	0.088491	100.0	0.108810	100.0	0.088491
200.0	0.057242	200.0	0.070276	200.0	0.088491	200.0	0.108810	200.0	0.088491
