# Supplementary material for: Benchmark Study of the Electronic States of the LiRb Molecule: Ab Initio Calculations with the Fock Space Coupled Cluster Approach
Source: Molecules. 2023 Nov 17;28(22):7645. doi: 10.3390/molecules28227645 (PMC10675596; doi:10.3390/molecules28227645)
Supplement: Supplementary file 1 [file molecules-28-07645-s001.zip › lirb_sapporo_sigma_plus_singlet.pdf]

#R[A]	X^1 sigma^+	R[A]	2^1 sigma^+	R[A]	3^1 sigma^+	R[A]	4^1 sigma^+	R[A]	5^1 sigma^+	R[A]	6^1 sigma^+
1.4	-2986.633488	1.4	-2986.554942	1.4	-2986.504663	1.4	-2986.499940	1.4	-2986.493536	1.4	-2986.482006
1.6	-2986.789116	1.6	-2986.713370	1.6	-2986.671246	1.6	-2986.660197	1.6	-2986.657671	1.6	-2986.643702
1.8	-2986.865071	1.8	-2986.789818	1.8	-2986.755562	1.8	-2986.740682	1.8	-2986.737164	1.8	-2986.722799
2.0	-2986.906222	2.0	-2986.830535	2.0	-2986.801950	2.0	-2986.785211	2.0	-2986.779447	2.0	-2986.766479
2.2	-2986.931539	2.2	-2986.855748	2.2	-2986.830203	2.2	-2986.813409	2.2	-2986.805457	2.2	-2986.793912
2.4	-2986.948876	2.4	-2986.874259	2.4	-2986.849447	2.4	-2986.833173	2.4	-2986.824032	2.4	-2986.813406
2.6	-2986.961532	2.6	-2986.889367	2.6	-2986.863924	2.6	-2986.847889	2.6	-2986.838502	2.6	-2986.828399
2.8	-2986.970454	2.8	-2986.901453	2.8	-2986.875235	2.8	-2986.858887	2.8	-2986.849858	2.8	-2986.839982
3.0	-2986.976292	3.0	-2986.910744	3.0	-2986.883968	3.0	-2986.866929	3.0	-2986.858657	3.0	-2986.848681
3.2	-2986.979621	3.2	-2986.917549	3.2	-2986.890479	3.2	-2986.872516	3.2	-2986.865361	3.2	-2986.854958
3.4	-2986.981016	3.4	-2986.922310	3.4	-2986.895089	3.4	-2986.876118	3.4	-2986.870403	3.4	-2986.859274
3.6	-2986.980931	3.6	-2986.925438	3.6	-2986.898276	3.6	-2986.878132	3.6	-2986.874190	3.6	-2986.862077
3.8	-2986.979792	3.8	-2986.927317	3.8	-2986.900287	3.8	-2986.878951	3.8	-2986.876964	3.8	-2986.863664
4.0	-2986.977956	4.0	-2986.928266	4.0	-2986.901419	4.0	-2986.879296	4.0	-2986.878599	4.0	-2986.864338
4.2	-2986.975701	4.2	-2986.928534	4.2	-2986.901880	4.2	-2986.880629	4.2	-2986.878063	4.2	-2986.864327
4.4	-2986.973244	4.4	-2986.928311	4.4	-2986.901824	4.4	-2986.881668	4.4	-2986.876979	4.4	-2986.863825
4.6	-2986.970748	4.6	-2986.927740	4.6	-2986.901366	4.6	-2986.882377	4.6	-2986.875661	4.6	-2986.862990
4.8	-2986.968333	4.8	-2986.926915	4.8	-2986.900602	4.8	-2986.882839	4.8	-2986.874253	4.8	-2986.861944
5.0	-2986.966085	5.0	-2986.925896	5.0	-2986.899611	5.0	-2986.883129	5.0	-2986.872864	5.0	-2986.860777
5.2	-2986.964057	5.2	-2986.924721	5.2	-2986.898459	5.2	-2986.883311	5.2	-2986.871571	5.2	-2986.859549
5.4	-2986.962280	5.4	-2986.923409	5.4	-2986.897204	5.4	-2986.883442	5.4	-2986.870417	5.4	-2986.858309
5.6	-2986.960762	5.6	-2986.921980	5.6	-2986.895899	5.6	-2986.883559	5.6	-2986.869422	5.6	-2986.857101
5.8	-2986.959493	5.8	-2986.920455	5.8	-2986.894590	5.8	-2986.883686	5.8	-2986.868588	5.8	-2986.856000
6.0	-2986.958451	6.0	-2986.918854	6.0	-2986.893321	6.0	-2986.883832	6.0	-2986.867901	6.0	-2986.855162
6.2	-2986.957609	6.2	-2986.917203	6.2	-2986.892122	6.2	-2986.883995	6.2	-2986.867342	6.2	-2986.854803
6.4	-2986.956935	6.4	-2986.915526	6.4	-2986.891022	6.4	-2986.884164	6.4	-2986.866888	6.4	-2986.854926
6.6	-2986.956403	6.6	-2986.913845	6.6	-2986.890058	6.6	-2986.884432	6.6	-2986.866508	6.6	-2986.855259
6.8	-2986.955981	6.8	-2986.912189	6.8	-2986.889208	6.8	-2986.884553	6.8	-2986.866213	6.8	-2986.855745
7.0	-2986.955649	7.0	-2986.910576	7.0	-2986.888493	7.0	-2986.884630	7.0	-2986.865976	7.0	-2986.856256
7.2	-2986.955391	7.2	-2986.909045	7.2	-2986.887889	7.2	-2986.884631	7.2	-2986.865790	7.2	-2986.856765
7.4	-2986.955186	7.4	-2986.907577	7.4	-2986.887411	7.4	-2986.884605	7.4	-2986.865641	7.4	-2986.857256
7.6	-2986.955023	7.6	-2986.906201	7.6	-2986.887007	7.6	-2986.884529	7.6	-2986.865521	7.6	-2986.857720
7.8	-2986.954895	7.8	-2986.904932	7.8	-2986.886639	7.8	-2986.884420	7.8	-2986.865426	7.8	-2986.858157
8.0	-2986.954793	8.0	-2986.903778	8.0	-2986.886274	8.0	-2986.884293	8.0	-2986.865352	8.0	-2986.858567
8.2	-2986.954712	8.2	-2986.902745	8.2	-2986.885888	8.2	-2986.884155	8.2	-2986.865295	8.2	-2986.858950
8.4	-2986.954648	8.4	-2986.901837	8.4	-2986.885477	8.4	-2986.883996	8.4	-2986.865254	8.4	-2986.859307
8.6	-2986.954596	8.6	-2986.901050	8.6	-2986.885067	8.6	-2986.883784	8.6	-2986.865224	8.6	-2986.859640
8.8	-2986.954554	8.8	-2986.900380	8.8	-2986.884717	8.8	-2986.883457	8.8	-2986.865205	8.8	-2986.859947
9.0	-2986.954520	9.0	-2986.899816	9.0	-2986.884478	9.0	-2986.882967	9.0	-2986.865193	9.0	-2986.860230
9.2	-2986.954492	9.2	-2986.899348	9.2	-2986.884334	9.2	-2986.882335	9.2	-2986.865189	9.2	-2986.860491
9.4	-2986.954468	9.4	-2986.898962	9.4	-2986.884247	9.4	-2986.881609	9.4	-2986.865190	9.4	-2986.860730
9.6	-2986.954449	9.6	-2986.898645	9.6	-2986.884191	9.6	-2986.880824	9.6	-2986.865194	9.6	-2986.860950
9.8	-2986.954433	9.8	-2986.898386	9.8	-2986.884153	9.8	-2986.880001	9.8	-2986.865202	9.8	-2986.861150
10.0	-2986.954419	10.0	-2986.898174	10.0	-2986.884126	10.0	-2986.879156	10.0	-2986.865212	10.0	-2986.861332
10.2	-2986.954407	10.2	-2986.898002	10.2	-2986.884107	10.2	-2986.878299	10.2	-2986.865224	10.2	-2986.861497
10.4	-2986.954397	10.4	-2986.897860	10.4	-2986.884092	10.4	-2986.877439	10.4	-2986.865238	10.4	-2986.861647
10.6	-2986.954388	10.6	-2986.897744	10.6	-2986.884082	10.6	-2986.876581	10.6	-2986.865251	10.6	-2986.861781
10.8	-2986.954380	10.8	-2986.897648	10.8	-2986.884073	10.8	-2986.875733	10.8	-2986.865264	10.8	-2986.861902
11.0	-2986.954373	11.0	-2986.897568	11.0	-2986.884066	11.0	-2986.874896	11.0	-2986.865277	11.0	-2986.862010
11.2	-2986.954367	11.2	-2986.897502	11.2	-2986.884061	11.2	-2986.874075	11.2	-2986.865288	11.2	-2986.862105
11.4	-2986.954362	11.4	-2986.897447	11.4	-2986.884056	11.4	-2986.873272	11.4	-2986.865297	11.4	-2986.862188
11.6	-2986.954357	11.6	-2986.897400	11.6	-2986.884052	11.6	-2986.872489	11.6	-2986.865303	11.6	-2986.862261
11.8	-2986.954353	11.8	-2986.897361	11.8	-2986.884049	11.8	-2986.871729	11.8	-2986.865305	11.8	-2986.862322
12.0	-2986.954349	12.0	-2986.897327	12.0	-2986.884046	12.0	-2986.870994	12.0	-2986.865304	12.0	-2986.862372
12.2	-2986.954345	12.2	-2986.897298	12.2	-2986.884043	12.2	-2986.870287	12.2	-2986.865296	12.2	-2986.862411
12.4	-2986.954342	12.4	-2986.897274	12.4	-2986.884041	12.4	-2986.869611	12.4	-2986.865280	12.4	-2986.862438
12.6	-2986.954339	12.6	-2986.897252	12.6	-2986.884039	12.6	-2986.868971	12.6	-2986.865252	12.6	-2986.862452
12.8	-2986.954337	12.8	-2986.897234	12.8	-2986.884037	12.8	-2986.868372	12.8	-2986.865210	12.8	-2986.862452
13.0	-2986.954334	13.0	-2986.897218	13.0	-2986.884036	13.0	-2986.867826	13.0	-2986.865145	13.0	-2986.862435
13.2	-2986.954332	13.2	-2986.897204	13.2	-2986.884034	13.2	-2986.867345	13.2	-2986.865050	13.2	-2986.862396
13.4	-2986.954330	13.4	-2986.897191	13.4	-2986.884033	13.4	-2986.866944	13.4	-2986.864916	13.4	-2986.862329
13.6	-2986.954329	13.6	-2986.897180	13.6	-2986.884032	13.6	-2986.866632	13.6	-2986.864741	13.6	-2986.862225
13.8	-2986.954327	13.8	-2986.897170	13.8	-2986.884031	13.8	-2986.866406	13.8	-2986.864538	13.8	-2986.862077
14.0	-2986.954326	14.0	-2986.897162	14.0	-2986.884031	14.0	-2986.866251	14.0	-2986.864331	14.0	-2986.861875
14.2	-2986.954325	14.2	-2986.897154	14.2	-2986.884030	14.2	-2986.866145	14.2	-2986.864143	14.2	-2986.861616
14.4	-2986.954323	14.4	-2986.897147	14.4	-2986.884030	14.4	-2986.866072	14.4	-2986.863990	14.4	-2986.861303
14.6	-2986.954322	14.6	-2986.897140	14.6	-2986.884030	14.6	-2986.866021	14.6	-2986.863869	14.6	-2986.860946
14.8	-2986.954322	14.8	-2986.897135	14.8	-2986.884029	14.8	-2986.865984	14.8	-2986.863779	14.8	-2986.860558
15.0	-2986.954321	15.0	-2986.897129	15.0	-2986.884029	15.0	-2986.865957	15.0	-2986.863711	15.0	-2986.860149
16.0	-2986.954318	16.0	-2986.897109	16.0	-2986.884030	16.0	-2986.865887	16.0	-2986.863553	16.0	-2986.858034
18.0	-2986.954315	18.0	-2986.897088	18.0	-2986.884032	18.0	-2986.865843	18.0	-2986.863485	18.0	-2986.854240
20.0	-2986.954314	20.0	-2986.897079	20.0	-2986.884034	20.0	-2986.865832	20.0	-2986.863470	20.0	-2986.851195
30.0	-2986.954313	30.0	-2986.897072	30.0	-2986.884037	30.0	-2986.865824	30.0	-2986.863460	30.0	-2986.845505
100.0	-2986.954313	100.0	-2986.897071	100.0	-2986.884037	100.0	-2986.865822	100.0	-2986.863459	100.0	-2986.845503
200.0	-2986.954313	200.0	-2986.897071	200.0	-2986.884037	200.0	-2986.865822	200.0	-2986.863459	200.0	-2986.845503
