# Supplementary material for: Benchmark Study of the Electronic States of the LiRb Molecule: Ab Initio Calculations with the Fock Space Coupled Cluster Approach
Source: Molecules. 2023 Nov 17;28(22):7645. doi: 10.3390/molecules28227645 (PMC10675596; doi:10.3390/molecules28227645)
Supplement: Supplementary file 1 [file molecules-28-07645-s001.zip › lirb_sapporo_sigma_plus_singlet_asymptotic.pdf]

#R[A]	X^1 sigma^+	R[A]	2^1 sigma^+	R[A]	3^1 sigma^+	R[A]	4^1 sigma^+	R[A]	5^1 sigma^+	R[A]	6^1 sigma^+
1.4	0.320825	1.4	0.399371	1.4	0.449650	1.4	0.454373	1.4	0.460777	1.4	0.472307
1.6	0.165197	1.6	0.240943	1.6	0.283067	1.6	0.294116	1.6	0.296642	1.6	0.310611
1.8	0.089242	1.8	0.164495	1.8	0.198751	1.8	0.213631	1.8	0.217149	1.8	0.231514
2.0	0.048091	2.0	0.123778	2.0	0.152363	2.0	0.169102	2.0	0.174866	2.0	0.187834
2.2	0.022774	2.2	0.098565	2.2	0.124110	2.2	0.140904	2.2	0.148856	2.2	0.160401
2.4	0.005437	2.4	0.080054	2.4	0.104866	2.4	0.121140	2.4	0.130281	2.4	0.140907
2.6	-0.007219	2.6	0.064946	2.6	0.090389	2.6	0.106424	2.6	0.115811	2.6	0.125914
2.8	-0.016141	2.8	0.052860	2.8	0.079078	2.8	0.095426	2.8	0.104455	2.8	0.114331
3.0	-0.021979	3.0	0.043569	3.0	0.070345	3.0	0.087384	3.0	0.095656	3.0	0.105632
3.2	-0.025308	3.2	0.036764	3.2	0.063834	3.2	0.081797	3.2	0.088952	3.2	0.099355
3.4	-0.026703	3.4	0.032003	3.4	0.059224	3.4	0.078195	3.4	0.083910	3.4	0.095039
3.6	-0.026618	3.6	0.028875	3.6	0.056037	3.6	0.076181	3.6	0.080123	3.6	0.092236
3.8	-0.025479	3.8	0.026996	3.8	0.054026	3.8	0.075362	3.8	0.077349	3.8	0.090649
4.0	-0.023643	4.0	0.026047	4.0	0.052894	4.0	0.075017	4.0	0.075714	4.0	0.089975
4.2	-0.021388	4.2	0.025779	4.2	0.052433	4.2	0.073684	4.2	0.076250	4.2	0.089986
4.4	-0.018931	4.4	0.026002	4.4	0.052489	4.4	0.072645	4.4	0.077334	4.4	0.090488
4.6	-0.016435	4.6	0.026573	4.6	0.052947	4.6	0.071936	4.6	0.078652	4.6	0.091323
4.8	-0.014020	4.8	0.027398	4.8	0.053711	4.8	0.071474	4.8	0.080060	4.8	0.092369
5.0	-0.011772	5.0	0.028417	5.0	0.054702	5.0	0.071184	5.0	0.081449	5.0	0.093536
5.2	-0.009744	5.2	0.029592	5.2	0.055854	5.2	0.071002	5.2	0.082742	5.2	0.094764
5.4	-0.007967	5.4	0.030904	5.4	0.057109	5.4	0.070871	5.4	0.083896	5.4	0.096004
5.6	-0.006449	5.6	0.032333	5.6	0.058414	5.6	0.070754	5.6	0.084891	5.6	0.097212
5.8	-0.005180	5.8	0.033858	5.8	0.059723	5.8	0.070627	5.8	0.085725	5.8	0.098313
6.0	-0.004138	6.0	0.035459	6.0	0.060992	6.0	0.070481	6.0	0.086412	6.0	0.099151
6.2	-0.003296	6.2	0.037110	6.2	0.062191	6.2	0.070318	6.2	0.086971	6.2	0.099510
6.4	-0.002622	6.4	0.038787	6.4	0.063291	6.4	0.070149	6.4	0.087425	6.4	0.099387
6.6	-0.002090	6.6	0.040468	6.6	0.064255	6.6	0.069881	6.6	0.087805	6.6	0.099054
6.8	-0.001668	6.8	0.042124	6.8	0.065105	6.8	0.069760	6.8	0.088100	6.8	0.098568
7.0	-0.001336	7.0	0.043737	7.0	0.065820	7.0	0.069683	7.0	0.088337	7.0	0.098057
7.2	-0.001078	7.2	0.045268	7.2	0.066424	7.2	0.069682	7.2	0.088523	7.2	0.097548
7.4	-0.000873	7.4	0.046736	7.4	0.066902	7.4	0.069708	7.4	0.088672	7.4	0.097057
7.6	-0.000710	7.6	0.048112	7.6	0.067306	7.6	0.069784	7.6	0.088792	7.6	0.096593
7.8	-0.000582	7.8	0.049381	7.8	0.067674	7.8	0.069893	7.8	0.088887	7.8	0.096156
8.0	-0.000480	8.0	0.050535	8.0	0.068039	8.0	0.070020	8.0	0.088961	8.0	0.095746
8.2	-0.000399	8.2	0.051568	8.2	0.068425	8.2	0.070158	8.2	0.089018	8.2	0.095363
8.4	-0.000335	8.4	0.052476	8.4	0.068836	8.4	0.070317	8.4	0.089059	8.4	0.095006
8.6	-0.000283	8.6	0.053263	8.6	0.069246	8.6	0.070529	8.6	0.089089	8.6	0.094673
8.8	-0.000241	8.8	0.053933	8.8	0.069596	8.8	0.070856	8.8	0.089108	8.8	0.094366
9.0	-0.000207	9.0	0.054497	9.0	0.069835	9.0	0.071346	9.0	0.089120	9.0	0.094083
9.2	-0.000179	9.2	0.054965	9.2	0.069979	9.2	0.071978	9.2	0.089124	9.2	0.093822
9.4	-0.000155	9.4	0.055351	9.4	0.070066	9.4	0.072704	9.4	0.089123	9.4	0.093583
9.6	-0.000136	9.6	0.055668	9.6	0.070122	9.6	0.073489	9.6	0.089119	9.6	0.093363
9.8	-0.000120	9.8	0.055927	9.8	0.070160	9.8	0.074312	9.8	0.089111	9.8	0.093163
10.0	-0.000106	10.0	0.056139	10.0	0.070187	10.0	0.075157	10.0	0.089101	10.0	0.092981
10.2	-0.000094	10.2	0.056311	10.2	0.070206	10.2	0.076014	10.2	0.089089	10.2	0.092816
10.4	-0.000084	10.4	0.056453	10.4	0.070221	10.4	0.076874	10.4	0.089075	10.4	0.092666
10.6	-0.000075	10.6	0.056569	10.6	0.070231	10.6	0.077732	10.6	0.089062	10.6	0.092532
10.8	-0.000067	10.8	0.056665	10.8	0.070240	10.8	0.078580	10.8	0.089049	10.8	0.092411
11.0	-0.000060	11.0	0.056745	11.0	0.070247	11.0	0.079417	11.0	0.089036	11.0	0.092303
11.2	-0.000054	11.2	0.056811	11.2	0.070252	11.2	0.080238	11.2	0.089025	11.2	0.092208
11.4	-0.000049	11.4	0.056866	11.4	0.070257	11.4	0.081041	11.4	0.089016	11.4	0.092125
11.6	-0.000044	11.6	0.056913	11.6	0.070261	11.6	0.081824	11.6	0.089010	11.6	0.092052
11.8	-0.000040	11.8	0.056952	11.8	0.070264	11.8	0.082584	11.8	0.089008	11.8	0.091991
12.0	-0.000036	12.0	0.056986	12.0	0.070267	12.0	0.083319	12.0	0.089009	12.0	0.091941
12.2	-0.000032	12.2	0.057015	12.2	0.070270	12.2	0.084026	12.2	0.089017	12.2	0.091902
12.4	-0.000029	12.4	0.057039	12.4	0.070272	12.4	0.084702	12.4	0.089033	12.4	0.091875
12.6	-0.000026	12.6	0.057061	12.6	0.070274	12.6	0.085342	12.6	0.089061	12.6	0.091861
12.8	-0.000024	12.8	0.057079	12.8	0.070276	12.8	0.085941	12.8	0.089103	12.8	0.091861
13.0	-0.000021	13.0	0.057095	13.0	0.070277	13.0	0.086487	13.0	0.089168	13.0	0.091878
13.2	-0.000019	13.2	0.057109	13.2	0.070279	13.2	0.086968	13.2	0.089263	13.2	0.091917
13.4	-0.000017	13.4	0.057122	13.4	0.070280	13.4	0.087369	13.4	0.089397	13.4	0.091984
13.6	-0.000016	13.6	0.057133	13.6	0.070281	13.6	0.087681	13.6	0.089572	13.6	0.092088
13.8	-0.000014	13.8	0.057143	13.8	0.070282	13.8	0.087907	13.8	0.089775	13.8	0.092236
14.0	-0.000013	14.0	0.057151	14.0	0.070282	14.0	0.088062	14.0	0.089982	14.0	0.092438
14.2	-0.000012	14.2	0.057159	14.2	0.070283	14.2	0.088168	14.2	0.090170	14.2	0.092697
14.4	-0.000010	14.4	0.057166	14.4	0.070283	14.4	0.088241	14.4	0.090323	14.4	0.093010
14.6	-0.000009	14.6	0.057173	14.6	0.070283	14.6	0.088292	14.6	0.090444	14.6	0.093367
14.8	-0.000009	14.8	0.057178	14.8	0.070284	14.8	0.088329	14.8	0.090534	14.8	0.093755
15.0	-0.000008	15.0	0.057184	15.0	0.070284	15.0	0.088356	15.0	0.090602	15.0	0.094164
16.0	-0.000005	16.0	0.057204	16.0	0.070283	16.0	0.088426	16.0	0.090760	16.0	0.096279
18.0	-0.000002	18.0	0.057225	18.0	0.070281	18.0	0.088470	18.0	0.090828	18.0	0.100073
20.0	-0.000001	20.0	0.057234	20.0	0.070279	20.0	0.088481	20.0	0.090843	20.0	0.103118
30.0	0.000000	30.0	0.057241	30.0	0.070276	30.0	0.088489	30.0	0.090853	30.0	0.108808
100.0	0.000000	100.0	0.057242	100.0	0.070276	100.0	0.088491	100.0	0.090854	100.0	0.108810
200.0	0.000000	200.0	0.057242	200.0	0.070276	200.0	0.088491	200.0	0.090854	200.0	0.108810
