# Supplementary material for: Benchmark Study of the Electronic States of the LiRb Molecule: Ab Initio Calculations with the Fock Space Coupled Cluster Approach
Source: Molecules. 2023 Nov 17;28(22):7645. doi: 10.3390/molecules28227645 (PMC10675596; doi:10.3390/molecules28227645)
Supplement: Supplementary file 1 [file molecules-28-07645-s001.zip › lirb_sapporo_sigma_plus_triplet.pdf]

#R[A]	1^3 sigma^+	R[A]	2^3 sigma^+	R[A]	3^3 sigma^+	R[A]	4^3 sigma^+	R[A]	5^3 sigma^+	R[A]	6^3 sigma^+
1.4	-2986.579468	1.4	-2986.523682	1.4	-2986.516280	1.4	-2986.488043	1.4	-2986.485063	1.4	-2986.479378
1.6	-2986.740582	1.6	-2986.681510	1.6	-2986.679711	1.6	-2986.649502	1.6	-2986.647505	1.6	-2986.643607
1.8	-2986.819781	1.8	-2986.759635	1.8	-2986.757503	1.8	-2986.727274	1.8	-2986.725652	1.8	-2986.722363
2.0	-2986.862304	2.0	-2986.802134	2.0	-2986.797322	2.0	-2986.768528	2.0	-2986.765314	2.0	-2986.762936
2.2	-2986.887684	2.2	-2986.827567	2.2	-2986.822529	2.2	-2986.795998	2.2	-2986.790651	2.2	-2986.784763
2.4	-2986.904835	2.4	-2986.848014	2.4	-2986.844418	2.4	-2986.816613	2.4	-2986.812445	2.4	-2986.797234
2.6	-2986.917774	2.6	-2986.867918	2.6	-2986.857750	2.6	-2986.833147	2.6	-2986.828519	2.6	-2986.813579
2.8	-2986.927886	2.8	-2986.883207	2.8	-2986.867992	2.8	-2986.845980	2.8	-2986.840503	2.8	-2986.826418
3.0	-2986.935774	3.0	-2986.894377	3.0	-2986.875712	3.0	-2986.855579	3.0	-2986.849244	3.0	-2986.836073
3.2	-2986.941799	3.2	-2986.902197	3.2	-2986.881233	3.2	-2986.862489	3.2	-2986.855350	3.2	-2986.843020
3.4	-2986.946268	3.4	-2986.907436	3.4	-2986.884875	3.4	-2986.867257	3.4	-2986.859418	3.4	-2986.847800
3.6	-2986.949527	3.6	-2986.910818	3.6	-2986.887056	3.6	-2986.870374	3.6	-2986.862058	3.6	-2986.851093
3.8	-2986.951824	3.8	-2986.912755	3.8	-2986.888103	3.8	-2986.872236	3.8	-2986.863644	3.8	-2986.854282
4.0	-2986.953401	4.0	-2986.913672	4.0	-2986.888310	4.0	-2986.873179	4.0	-2986.864771	4.0	-2986.857540
4.2	-2986.954446	4.2	-2986.913854	4.2	-2986.887925	4.2	-2986.873455	4.2	-2986.866074	4.2	-2986.859491
4.4	-2986.955105	4.4	-2986.913512	4.4	-2986.887153	4.4	-2986.873259	4.4	-2986.867821	4.4	-2986.860006
4.6	-2986.955494	4.6	-2986.912808	4.6	-2986.886168	4.6	-2986.872748	4.6	-2986.869666	4.6	-2986.859622
4.8	-2986.955698	4.8	-2986.911872	4.8	-2986.885113	4.8	-2986.872109	4.8	-2986.871242	4.8	-2986.858804
5.0	-2986.955778	5.0	-2986.910804	5.0	-2986.884110	5.0	-2986.872769	5.0	-2986.871093	5.0	-2986.857785
5.2	-2986.955777	5.2	-2986.909675	5.2	-2986.883267	5.2	-2986.873726	5.2	-2986.870236	5.2	-2986.856677
5.4	-2986.955724	5.4	-2986.908537	5.4	-2986.882676	5.4	-2986.874298	5.4	-2986.869368	5.4	-2986.855544
5.6	-2986.955640	5.6	-2986.907426	5.6	-2986.882377	5.6	-2986.874471	5.6	-2986.868547	5.6	-2986.854421
5.8	-2986.955540	5.8	-2986.906366	5.8	-2986.882334	5.8	-2986.874310	5.8	-2986.867801	5.8	-2986.853331
6.0	-2986.955434	6.0	-2986.905372	6.0	-2986.882460	6.0	-2986.873928	6.0	-2986.867144	6.0	-2986.852289
6.2	-2986.955326	6.2	-2986.904454	6.2	-2986.882665	6.2	-2986.873429	6.2	-2986.866584	6.2	-2986.851306
6.4	-2986.955221	6.4	-2986.903616	6.4	-2986.882891	6.4	-2986.872885	6.4	-2986.866115	6.4	-2986.850388
6.6	-2986.955130	6.6	-2986.902882	6.6	-2986.883126	6.6	-2986.872328	6.6	-2986.865732	6.6	-2986.849520
6.8	-2986.955038	6.8	-2986.902200	6.8	-2986.883310	6.8	-2986.871796	6.8	-2986.865420	6.8	-2986.848763
7.0	-2986.954953	7.0	-2986.901592	7.0	-2986.883465	7.0	-2986.871290	7.0	-2986.865170	7.0	-2986.848108
7.2	-2986.954875	7.2	-2986.901053	7.2	-2986.883598	7.2	-2986.870816	7.2	-2986.864975	7.2	-2986.847603
7.4	-2986.954808	7.4	-2986.900580	7.4	-2986.883699	7.4	-2986.870373	7.4	-2986.864818	7.4	-2986.847308
7.6	-2986.954748	7.6	-2986.900161	7.6	-2986.883779	7.6	-2986.869963	7.6	-2986.864692	7.6	-2986.847224
7.8	-2986.954695	7.8	-2986.899791	7.8	-2986.883842	7.8	-2986.869584	7.8	-2986.864592	7.8	-2986.847277
8.0	-2986.954648	8.0	-2986.899466	8.0	-2986.883890	8.0	-2986.869234	8.0	-2986.864512	8.0	-2986.847392
8.2	-2986.954607	8.2	-2986.899181	8.2	-2986.883928	8.2	-2986.868909	8.2	-2986.864446	8.2	-2986.847529
8.4	-2986.954572	8.4	-2986.898931	8.4	-2986.883958	8.4	-2986.868610	8.4	-2986.864391	8.4	-2986.847667
8.6	-2986.954542	8.6	-2986.898711	8.6	-2986.883981	8.6	-2986.868335	8.6	-2986.864344	8.6	-2986.847795
8.8	-2986.954515	8.8	-2986.898519	8.8	-2986.883999	8.8	-2986.868081	8.8	-2986.864304	8.8	-2986.847908
9.0	-2986.954492	9.0	-2986.898350	9.0	-2986.884013	9.0	-2986.867848	9.0	-2986.864267	9.0	-2986.848001
9.2	-2986.954472	9.2	-2986.898202	9.2	-2986.884024	9.2	-2986.867634	9.2	-2986.864232	9.2	-2986.848075
9.4	-2986.954454	9.4	-2986.898072	9.4	-2986.884032	9.4	-2986.867439	9.4	-2986.864198	9.4	-2986.848128
9.6	-2986.954439	9.6	-2986.897958	9.6	-2986.884038	9.6	-2986.867261	9.6	-2986.864165	9.6	-2986.848162
9.8	-2986.954426	9.8	-2986.897857	9.8	-2986.884043	9.8	-2986.867101	9.8	-2986.864131	9.8	-2986.848176
10.0	-2986.954414	10.0	-2986.897769	10.0	-2986.884046	10.0	-2986.866956	10.0	-2986.864098	10.0	-2986.848172
10.2	-2986.954403	10.2	-2986.897691	10.2	-2986.884048	10.2	-2986.866827	10.2	-2986.864064	10.2	-2986.848151
10.4	-2986.954394	10.4	-2986.897623	10.4	-2986.884049	10.4	-2986.866711	10.4	-2986.864029	10.4	-2986.848115
10.6	-2986.954386	10.6	-2986.897563	10.6	-2986.884049	10.6	-2986.866608	10.6	-2986.863994	10.6	-2986.848066
10.8	-2986.954379	10.8	-2986.897510	10.8	-2986.884049	10.8	-2986.866518	10.8	-2986.863959	10.8	-2986.848005
11.0	-2986.954372	11.0	-2986.897464	11.0	-2986.884049	11.0	-2986.866438	11.0	-2986.863925	11.0	-2986.847934
11.2	-2986.954367	11.2	-2986.897423	11.2	-2986.884048	11.2	-2986.866367	11.2	-2986.863892	11.2	-2986.847854
11.4	-2986.954361	11.4	-2986.897386	11.4	-2986.884046	11.4	-2986.866305	11.4	-2986.863859	11.4	-2986.847767
11.6	-2986.954357	11.6	-2986.897354	11.6	-2986.884045	11.6	-2986.866250	11.6	-2986.863828	11.6	-2986.847674
11.8	-2986.954353	11.8	-2986.897326	11.8	-2986.884044	11.8	-2986.866203	11.8	-2986.863798	11.8	-2986.847576
12.0	-2986.954349	12.0	-2986.897300	12.0	-2986.884042	12.0	-2986.866161	12.0	-2986.863770	12.0	-2986.847476
12.2	-2986.954345	12.2	-2986.897278	12.2	-2986.884041	12.2	-2986.866124	12.2	-2986.863743	12.2	-2986.847373
12.4	-2986.954342	12.4	-2986.897259	12.4	-2986.884039	12.4	-2986.866092	12.4	-2986.863719	12.4	-2986.847269
12.6	-2986.954339	12.6	-2986.897241	12.6	-2986.884038	12.6	-2986.866064	12.6	-2986.863696	12.6	-2986.847166
12.8	-2986.954337	12.8	-2986.897225	12.8	-2986.884036	12.8	-2986.866040	12.8	-2986.863675	12.8	-2986.847063
13.0	-2986.954334	13.0	-2986.897211	13.0	-2986.884035	13.0	-2986.866019	13.0	-2986.863655	13.0	-2986.846963
13.2	-2986.954332	13.2	-2986.897199	13.2	-2986.884034	13.2	-2986.866000	13.2	-2986.863637	13.2	-2986.846864
13.4	-2986.954330	13.4	-2986.897187	13.4	-2986.884033	13.4	-2986.865984	13.4	-2986.863621	13.4	-2986.846769
13.6	-2986.954329	13.6	-2986.897177	13.6	-2986.884032	13.6	-2986.865970	13.6	-2986.863606	13.6	-2986.846677
13.8	-2986.954327	13.8	-2986.897168	13.8	-2986.884031	13.8	-2986.865958	13.8	-2986.863593	13.8	-2986.846589
14.0	-2986.954326	14.0	-2986.897160	14.0	-2986.884031	14.0	-2986.865946	14.0	-2986.863580	14.0	-2986.846505
14.2	-2986.954325	14.2	-2986.897152	14.2	-2986.884030	14.2	-2986.865936	14.2	-2986.863569	14.2	-2986.846425
14.4	-2986.954323	14.4	-2986.897146	14.4	-2986.884030	14.4	-2986.865926	14.4	-2986.863560	14.4	-2986.846349
14.6	-2986.954322	14.6	-2986.897140	14.6	-2986.884030	14.6	-2986.865917	14.6	-2986.863551	14.6	-2986.846277
14.8	-2986.954322	14.8	-2986.897134	14.8	-2986.884029	14.8	-2986.865910	14.8	-2986.863542	14.8	-2986.846210
15.0	-2986.954321	15.0	-2986.897129	15.0	-2986.884029	15.0	-2986.865903	15.0	-2986.863535	15.0	-2986.846147
16.0	-2986.954318	16.0	-2986.897108	16.0	-2986.884030	16.0	-2986.865874	16.0	-2986.863508	16.0	-2986.845894
18.0	-2986.954315	18.0	-2986.897088	18.0	-2986.884032	18.0	-2986.865843	18.0	-2986.863481	18.0	-2986.845627
20.0	-2986.954314	20.0	-2986.897079	20.0	-2986.884034	20.0	-2986.865832	20.0	-2986.863470	20.0	-2986.845539
30.0	-2986.954313	30.0	-2986.897072	30.0	-2986.884037	30.0	-2986.865824	30.0	-2986.863460	30.0	-2986.845505
100.0	-2986.954313	100.0	-2986.897071	100.0	-2986.884037	100.0	-2986.865822	100.0	-2986.863459	100.0	-2986.845503
200.0	-2986.954313	200.0	-2986.897071	200.0	-2986.884037	200.0	-2986.865822	200.0	-2986.863459	200.0	-2986.845503
