# Supplementary material for: Benchmark Study of the Electronic States of the LiRb Molecule: Ab Initio Calculations with the Fock Space Coupled Cluster Approach
Source: Molecules. 2023 Nov 17;28(22):7645. doi: 10.3390/molecules28227645 (PMC10675596; doi:10.3390/molecules28227645)
Supplement: Supplementary file 1 [file molecules-28-07645-s001.zip › lirb_sapporo_sigma_plus_triplet_asymptotic.pdf]

#R[A]	1^3 sigma^+	R[A]	2^3 sigma^+	R[A]	3^3 sigma^+	R[A]	4^3 sigma^+	R[A]	5^3 sigma^+	R[A]	6^3 sigma^+
1.4	0.374845	1.4	0.430631	1.4	0.438033	1.4	0.466270	1.4	0.469250	1.4	0.474935
1.6	0.213731	1.6	0.272803	1.6	0.274602	1.6	0.304811	1.6	0.306808	1.6	0.310706
1.8	0.134532	1.8	0.194678	1.8	0.196810	1.8	0.227039	1.8	0.228661	1.8	0.231950
2.0	0.092009	2.0	0.152179	2.0	0.156991	2.0	0.185785	2.0	0.188999	2.0	0.191377
2.2	0.066629	2.2	0.126746	2.2	0.131784	2.2	0.158315	2.2	0.163662	2.2	0.169550
2.4	0.049478	2.4	0.106299	2.4	0.109895	2.4	0.137700	2.4	0.141868	2.4	0.157079
2.6	0.036539	2.6	0.086395	2.6	0.096563	2.6	0.121166	2.6	0.125794	2.6	0.140734
2.8	0.026427	2.8	0.071106	2.8	0.086321	2.8	0.108333	2.8	0.113810	2.8	0.127895
3.0	0.018539	3.0	0.059936	3.0	0.078601	3.0	0.098734	3.0	0.105069	3.0	0.118240
3.2	0.012514	3.2	0.052116	3.2	0.073080	3.2	0.091824	3.2	0.098963	3.2	0.111293
3.4	0.008045	3.4	0.046877	3.4	0.069438	3.4	0.087056	3.4	0.094895	3.4	0.106513
3.6	0.004786	3.6	0.043495	3.6	0.067257	3.6	0.083939	3.6	0.092255	3.6	0.103220
3.8	0.002489	3.8	0.041558	3.8	0.066210	3.8	0.082077	3.8	0.090669	3.8	0.100031
4.0	0.000912	4.0	0.040641	4.0	0.066003	4.0	0.081134	4.0	0.089542	4.0	0.096773
4.2	-0.000133	4.2	0.040459	4.2	0.066388	4.2	0.080858	4.2	0.088239	4.2	0.094822
4.4	-0.000792	4.4	0.040801	4.4	0.067160	4.4	0.081054	4.4	0.086492	4.4	0.094307
4.6	-0.001181	4.6	0.041505	4.6	0.068145	4.6	0.081565	4.6	0.084647	4.6	0.094691
4.8	-0.001385	4.8	0.042441	4.8	0.069200	4.8	0.082204	4.8	0.083071	4.8	0.095509
5.0	-0.001465	5.0	0.043509	5.0	0.070203	5.0	0.081544	5.0	0.083220	5.0	0.096528
5.2	-0.001464	5.2	0.044638	5.2	0.071046	5.2	0.080587	5.2	0.084077	5.2	0.097636
5.4	-0.001411	5.4	0.045776	5.4	0.071637	5.4	0.080015	5.4	0.084945	5.4	0.098769
5.6	-0.001327	5.6	0.046887	5.6	0.071936	5.6	0.079842	5.6	0.085766	5.6	0.099892
5.8	-0.001227	5.8	0.047947	5.8	0.071979	5.8	0.080003	5.8	0.086512	5.8	0.100982
6.0	-0.001121	6.0	0.048941	6.0	0.071853	6.0	0.080385	6.0	0.087169	6.0	0.102024
6.2	-0.001013	6.2	0.049859	6.2	0.071648	6.2	0.080884	6.2	0.087729	6.2	0.103007
6.4	-0.000908	6.4	0.050697	6.4	0.071422	6.4	0.081428	6.4	0.088198	6.4	0.103925
6.6	-0.000817	6.6	0.051431	6.6	0.071187	6.6	0.081985	6.6	0.088581	6.6	0.104793
6.8	-0.000725	6.8	0.052113	6.8	0.071003	6.8	0.082517	6.8	0.088893	6.8	0.105550
7.0	-0.000640	7.0	0.052721	7.0	0.070848	7.0	0.083023	7.0	0.089143	7.0	0.106205
7.2	-0.000562	7.2	0.053260	7.2	0.070715	7.2	0.083497	7.2	0.089338	7.2	0.106710
7.4	-0.000495	7.4	0.053733	7.4	0.070614	7.4	0.083940	7.4	0.089495	7.4	0.107005
7.6	-0.000435	7.6	0.054152	7.6	0.070534	7.6	0.084350	7.6	0.089621	7.6	0.107089
7.8	-0.000382	7.8	0.054522	7.8	0.070471	7.8	0.084729	7.8	0.089721	7.8	0.107036
8.0	-0.000335	8.0	0.054847	8.0	0.070423	8.0	0.085079	8.0	0.089801	8.0	0.106921
8.2	-0.000294	8.2	0.055132	8.2	0.070385	8.2	0.085404	8.2	0.089867	8.2	0.106784
8.4	-0.000259	8.4	0.055382	8.4	0.070355	8.4	0.085703	8.4	0.089922	8.4	0.106646
8.6	-0.000229	8.6	0.055602	8.6	0.070332	8.6	0.085978	8.6	0.089969	8.6	0.106518
8.8	-0.000202	8.8	0.055794	8.8	0.070314	8.8	0.086232	8.8	0.090009	8.8	0.106405
9.0	-0.000179	9.0	0.055963	9.0	0.070300	9.0	0.086465	9.0	0.090046	9.0	0.106312
9.2	-0.000159	9.2	0.056111	9.2	0.070289	9.2	0.086679	9.2	0.090081	9.2	0.106238
9.4	-0.000141	9.4	0.056241	9.4	0.070281	9.4	0.086874	9.4	0.090115	9.4	0.106185
9.6	-0.000126	9.6	0.056355	9.6	0.070275	9.6	0.087052	9.6	0.090148	9.6	0.106151
9.8	-0.000113	9.8	0.056456	9.8	0.070270	9.8	0.087212	9.8	0.090182	9.8	0.106137
10.0	-0.000101	10.0	0.056544	10.0	0.070267	10.0	0.087357	10.0	0.090215	10.0	0.106141
10.2	-0.000090	10.2	0.056622	10.2	0.070265	10.2	0.087486	10.2	0.090249	10.2	0.106162
10.4	-0.000081	10.4	0.056690	10.4	0.070264	10.4	0.087602	10.4	0.090284	10.4	0.106198
10.6	-0.000073	10.6	0.056750	10.6	0.070264	10.6	0.087705	10.6	0.090319	10.6	0.106247
10.8	-0.000066	10.8	0.056803	10.8	0.070264	10.8	0.087795	10.8	0.090354	10.8	0.106308
11.0	-0.000059	11.0	0.056849	11.0	0.070264	11.0	0.087875	11.0	0.090388	11.0	0.106379
11.2	-0.000054	11.2	0.056890	11.2	0.070265	11.2	0.087946	11.2	0.090421	11.2	0.106459
11.4	-0.000048	11.4	0.056927	11.4	0.070267	11.4	0.088008	11.4	0.090454	11.4	0.106546
11.6	-0.000044	11.6	0.056959	11.6	0.070268	11.6	0.088063	11.6	0.090485	11.6	0.106639
11.8	-0.000040	11.8	0.056987	11.8	0.070269	11.8	0.088110	11.8	0.090515	11.8	0.106737
12.0	-0.000036	12.0	0.057013	12.0	0.070271	12.0	0.088152	12.0	0.090543	12.0	0.106837
12.2	-0.000032	12.2	0.057035	12.2	0.070272	12.2	0.088189	12.2	0.090570	12.2	0.106940
12.4	-0.000029	12.4	0.057054	12.4	0.070274	12.4	0.088221	12.4	0.090594	12.4	0.107044
12.6	-0.000026	12.6	0.057072	12.6	0.070275	12.6	0.088249	12.6	0.090617	12.6	0.107147
12.8	-0.000024	12.8	0.057088	12.8	0.070277	12.8	0.088273	12.8	0.090638	12.8	0.107250
13.0	-0.000021	13.0	0.057102	13.0	0.070278	13.0	0.088294	13.0	0.090658	13.0	0.107350
13.2	-0.000019	13.2	0.057114	13.2	0.070279	13.2	0.088313	13.2	0.090676	13.2	0.107449
13.4	-0.000017	13.4	0.057126	13.4	0.070280	13.4	0.088329	13.4	0.090692	13.4	0.107544
13.6	-0.000016	13.6	0.057136	13.6	0.070281	13.6	0.088343	13.6	0.090707	13.6	0.107636
13.8	-0.000014	13.8	0.057145	13.8	0.070282	13.8	0.088355	13.8	0.090720	13.8	0.107724
14.0	-0.000013	14.0	0.057153	14.0	0.070282	14.0	0.088367	14.0	0.090733	14.0	0.107808
14.2	-0.000012	14.2	0.057161	14.2	0.070283	14.2	0.088377	14.2	0.090744	14.2	0.107888
14.4	-0.000010	14.4	0.057167	14.4	0.070283	14.4	0.088387	14.4	0.090753	14.4	0.107964
14.6	-0.000009	14.6	0.057173	14.6	0.070283	14.6	0.088396	14.6	0.090762	14.6	0.108036
14.8	-0.000009	14.8	0.057179	14.8	0.070284	14.8	0.088403	14.8	0.090771	14.8	0.108103
15.0	-0.000008	15.0	0.057184	15.0	0.070284	15.0	0.088410	15.0	0.090778	15.0	0.108166
16.0	-0.000005	16.0	0.057205	16.0	0.070283	16.0	0.088439	16.0	0.090805	16.0	0.108419
18.0	-0.000002	18.0	0.057225	18.0	0.070281	18.0	0.088470	18.0	0.090832	18.0	0.108686
20.0	-0.000001	20.0	0.057234	20.0	0.070279	20.0	0.088481	20.0	0.090843	20.0	0.108774
30.0	0.000000	30.0	0.057241	30.0	0.070276	30.0	0.088489	30.0	0.090853	30.0	0.108808
100.0	0.000000	100.0	0.057242	100.0	0.070276	100.0	0.088491	100.0	0.090854	100.0	0.108810
200.0	0.000000	200.0	0.057242	200.0	0.070276	200.0	0.088491	200.0	0.090854	200.0	0.108810
