# Supplementary material for: Benchmark Study of the Electronic States of the LiRb Molecule: Ab Initio Calculations with the Fock Space Coupled Cluster Approach
Source: Molecules. 2023 Nov 17;28(22):7645. doi: 10.3390/molecules28227645 (PMC10675596; doi:10.3390/molecules28227645)
Supplement: Supplementary file 1 [file molecules-28-07645-s001.zip › lirb_unanorccplus_pi_delta_singlet.pdf]

#R[A]	1^1 pi	R[A]	2^1 pi	R[A]	3^1 pi	R[A]	4^1 pi	R[A]	1^1 delta
1.4	-2946.517079	1.4	-2946.491733	1.4	-2946.468323	1.4	-2946.442403	1.4	-2946.494179
1.5	-2946.612627	1.5	-2946.588069	1.6	-2946.638147	1.5	-2946.539556	1.5	-2946.592985
1.6	-2946.678670	1.6	-2946.654619	1.7	-2946.685969	1.6	-2946.607050	1.6	-2946.661500
1.7	-2946.723945	1.7	-2946.700714	1.8	-2946.718995	1.7	-2946.654222	1.7	-2946.708845
1.8	-2946.755491	1.8	-2946.733292	2.0	-2946.758417	1.8	-2946.687697	1.8	-2946.741877
1.9	-2946.777548	1.9	-2946.756914	2.1	-2946.770286	1.9	-2946.712081	1.9	-2946.765086
2.0	-2946.793316	2.1	-2946.787940	2.2	-2946.779266	2.0	-2946.730502	2.0	-2946.781653
2.1	-2946.804880	2.2	-2946.798863	2.3	-2946.786334	2.1	-2946.745154	2.2	-2946.802818
2.3	-2946.820871	2.3	-2946.807911	2.4	-2946.792182	2.2	-2946.757353	2.3	-2946.809865
2.4	-2946.827018	2.4	-2946.815532	2.5	-2946.797212	2.3	-2946.767861	2.4	-2946.815542
2.5	-2946.832592	2.5	-2946.821963	2.6	-2946.801671	2.4	-2946.777075	2.5	-2946.820266
2.6	-2946.837784	2.6	-2946.827380	2.7	-2946.805716	2.5	-2946.785183	2.6	-2946.824297
2.8	-2946.846962	2.7	-2946.831980	2.8	-2946.809427	2.6	-2946.792279	2.7	-2946.827791
2.9	-2946.850813	2.8	-2946.835940	2.9	-2946.812830	2.7	-2946.798433	2.8	-2946.830832
3.0	-2946.854124	2.9	-2946.839385	3.0	-2946.815924	2.8	-2946.803720	2.9	-2946.833467
3.05	-2946.855580	3.0	-2946.842388	3.05	-2946.817353	2.9	-2946.808226	3.0	-2946.835720
3.1	-2946.856906	3.05	-2946.843739	3.15	-2946.819972	3.0	-2946.812043	3.05	-2946.836708
3.15	-2946.858108	3.1	-2946.844994	3.2	-2946.821165	3.05	-2946.813719	3.1	-2946.837607
3.2	-2946.859190	3.2	-2946.847229	3.25	-2946.822283	3.1	-2946.815251	3.15	-2946.838419
3.25	-2946.860158	3.25	-2946.848216	3.3	-2946.823329	3.15	-2946.816649	3.2	-2946.839147
3.3	-2946.861019	3.3	-2946.849119	3.35	-2946.824306	3.2	-2946.817919	3.25	-2946.839793
3.35	-2946.861778	3.35	-2946.849943	3.45	-2946.826065	3.25	-2946.819067	3.3	-2946.840360
3.45	-2946.863019	3.45	-2946.851363	3.55	-2946.827554	3.3	-2946.820099	3.35	-2946.840851
3.55	-2946.863888	3.8	-2946.854292	3.8	-2946.830449	3.35	-2946.821021	3.45	-2946.841620
3.8	-2946.865106	3.85	-2946.854494	3.85	-2946.830884	3.45	-2946.822559	3.8	-2946.842454
3.85	-2946.865194	3.9	-2946.854650	3.9	-2946.831278	3.55	-2946.823715	3.85	-2946.842386
3.9	-2946.865246	4.0	-2946.854842	3.95	-2946.831634	3.8	-2946.825362	3.9	-2946.842282
3.95	-2946.865264	4.1	-2946.854877	4.0	-2946.831948	3.85	-2946.825510	3.95	-2946.842144
4.0	-2946.865253	4.2	-2946.854782	4.1	-2946.832487	3.9	-2946.825610	4.0	-2946.842047
4.1	-2946.865163	4.3	-2946.854577	4.2	-2946.832989	3.95	-2946.825666	4.1	-2946.841562
4.3	-2946.864802	4.4	-2946.854281	4.3	-2946.833294	4.0	-2946.825686	4.2	-2946.841130
4.4	-2946.864558	4.5	-2946.853913	4.4	-2946.833504	4.1	-2946.825616	4.3	-2946.840563
4.5	-2946.864291	4.6	-2946.853480	4.5	-2946.833593	4.2	-2946.825438	4.4	-2946.839947
4.6	-2946.864031	4.7	-2946.853017	4.6	-2946.833708	4.3	-2946.825157	4.5	-2946.839298
4.7	-2946.863753	4.75	-2946.852773	4.7	-2946.833696	4.4	-2946.824804	4.6	-2946.838629
4.75	-2946.863618	4.8	-2946.852525	4.75	-2946.833677	4.5	-2946.824393	4.7	-2946.837959
4.8	-2946.863485	4.9	-2946.852021	4.8	-2946.833646	4.6	-2946.823950	4.75	-2946.837624
4.85	-2946.863354	5.0	-2946.851517	4.85	-2946.833605	4.7	-2946.823462	4.8	-2946.837291
4.9	-2946.863224	5.1	-2946.851035	4.9	-2946.833554	4.75	-2946.823213	4.85	-2946.836961
5.0	-2946.862973	5.2	-2946.850548	5.0	-2946.833428	4.8	-2946.822959	4.9	-2946.836636
5.1	-2946.862724	5.3	-2946.850096	5.1	-2946.833276	4.8	-2946.822701	5.0	-2946.836001
5.3	-2946.862281	5.4	-2946.849673	5.2	-2946.833100	4.9	-2946.822440	5.1	-2946.835386
5.4	-2946.862072	5.5	-2946.849281	5.3	-2946.832911	5.0	-2946.821910	5.2	-2946.834816
5.5	-2946.861873	5.6	-2946.848918	5.4	-2946.832711	5.1	-2946.821412	5.3	-2946.834271
5.6	-2946.861718	5.7	-2946.848595	5.5	-2946.832505	5.2	-2946.820825	5.4	-2946.833763
5.7	-2946.861506	5.8	-2946.848301	5.6	-2946.832312	5.3	-2946.820272	5.5	-2946.833290
5.8	-2946.861337	5.9	-2946.848034	5.7	-2946.832091	5.4	-2946.819715	5.6	-2946.832853
5.9	-2946.861218	6.0	-2946.847804	5.8	-2946.831889	5.5	-2946.819157	5.7	-2946.832452
6.0	-2946.861026	6.2	-2946.847412	5.9	-2946.831701	5.6	-2946.818608	5.8	-2946.832085
6.2	-2946.860791	6.6	-2946.846885	6.0	-2946.831504	5.7	-2946.818026	6.0	-2946.831446
6.4	-2946.860541	6.8	-2946.846715	6.2	-2946.831157	5.8	-2946.817460	6.2	-2946.830922
6.6	-2946.860318	7.0	-2946.846584	6.4	-2946.830843	5.9	-2946.816920	6.4	-2946.830495
6.8	-2946.860076	7.4	-2946.846418	6.6	-2946.830567	6.0	-2946.816345	6.6	-2946.830148
7.0	-2946.859939	7.6	-2946.846366	6.8	-2946.830323	6.2	-2946.815297	6.8	-2946.829869
7.4	-2946.859610	7.8	-2946.846327	7.0	-2946.830115	6.4	-2946.814299	7.0	-2946.829643
7.6	-2946.859517	7.95	-2946.846305	7.4	-2946.829764	6.8	-2946.812589	7.4	-2946.829313
7.8	-2946.859386	8.0	-2946.846298	7.6	-2946.829627	7.0	-2946.811955	7.6	-2946.829193
7.95	-2946.859336	8.05	-2946.846292	7.8	-2946.829495	7.4	-2946.810932	7.8	-2946.829096
8.05	-2946.859291	8.2	-2946.846277	7.95	-2946.829416	7.6	-2946.810592	7.95	-2946.829035
8.2	-2946.859229	8.35	-2946.846263	8.0	-2946.829389	7.8	-2946.810278	8.0	-2946.829016
8.35	-2946.859159	8.4	-2946.846260	8.05	-2946.829363	8.0	-2946.810056	8.05	-2946.828999
8.4	-2946.859155	8.45	-2946.846257	8.2	-2946.829290	8.05	-2946.810003	8.2	-2946.828951
8.45	-2946.859138	8.5	-2946.846253	8.35	-2946.829221	8.2	-2946.809858	8.35	-2946.828910
8.5	-2946.859111	8.6	-2946.846248	8.4	-2946.829202	8.35	-2946.809722	8.4	-2946.828898
8.6	-2946.859091	8.8	-2946.846239	8.45	-2946.829182	8.4	-2946.809695	8.45	-2946.828886
8.8	-2946.859035	9.01	-2946.846231	8.5	-2946.829161	8.45	-2946.809659	8.5	-2946.828875
9.01	-2946.858977	9.2	-2946.846226	8.6	-2946.829125	8.5	-2946.809615	8.6	-2946.828854
9.2	-2946.858938	9.4	-2946.846221	8.8	-2946.829056	8.6	-2946.809559	8.8	-2946.828817
9.4	-2946.858900	9.6	-2946.846218	9.01	-2946.828997	8.8	-2946.809445	9.2	-2946.828761
9.6	-2946.858868	9.8	-2946.846216	9.4	-2946.828894	9.01	-2946.809339	9.6	-2946.828721
9.8	-2946.858840	10.0	-2946.846215	9.6	-2946.828856	9.2	-2946.809270	10.0	-2946.828691
10.0	-2946.858815	10.2	-2946.846213	9.8	-2946.828824	9.4	-2946.809198	10.2	-2946.828680
10.2	-2946.858794	10.4	-2946.846213	10.0	-2946.828797	9.6	-2946.809135	10.4	-2946.828670
10.4	-2946.858775	10.6	-2946.846212	10.2	-2946.828774	9.8	-2946.809080	10.6	-2946.828661
10.6	-2946.858759	10.8	-2946.846211	10.4	-2946.828755	10.0	-2946.809031	10.8	-2946.828653
10.8	-2946.858744	11.2	-2946.846211	10.6	-2946.828739	10.2	-2946.808988	11.2	-2946.828640
11.2	-2946.858721	11.4	-2946.846211	10.8	-2946.828725	10.6	-2946.808914	11.4	-2946.828634
11.4	-2946.858711	11.6	-2946.846212	11.2	-2946.828702	10.8	-2946.808882	11.6	-2946.828630
11.6	-2946.858701	11.8	-2946.846212	11.4	-2946.828694	11.2	-2946.808826	11.8	-2946.828625
11.8	-2946.858693	12.0	-2946.846212	11.6	-2946.828684	11.4	-2946.808802	12.0	-2946.828621
12.0	-2946.858686	12.2	-2946.846210	11.8	-2946.828677	11.6	-2946.808777	12.2	-2946.828618
12.2	-2946.858680	20.0	-2946.846212	12.0	-2946.828670	11.8	-2946.808757	12.4	-2946.828615
19.0	-2946.858622	21.0	-2946.846212	12.2	-2946.828666	12.0	-2946.808738	12.6	-2946.828612
20.0	-2946.858620	23.0	-2946.846212	19.0	-2946.828589	12.2	-2946.808722	12.8	-2946.828609
21.0	-2946.858619	24.0	-2946.846212	20.0	-2946.828587	19.0	-2946.808544	12.9	-2946.828608
23.0	-2946.858618	26.0	-2946.846212	21.0	-2946.828586	20.0	-2946.808540	13.0	-2946.828607
24.0	-2946.858618	27.0	-2946.846212	23.0	-2946.828584	21.0	-2946.808537	13.8	-2946.828600
26.0	-2946.858617	28.0	-2946.846212	24.0	-2946.828584	23.0	-2946.808534	13.9	-2946.828599
27.0	-2946.858617	29.0	-2946.846212	26.0	-2946.828583	24.0	-2946.808532	14.1	-2946.828598
28.0	-2946.858617	30.0	-2946.846212	27.0	-2946.828583	26.0	-2946.808531	14.2	-2946.828597
29.0	-2946.858616	32.0	-2946.846212	28.0	-2946.828583	27.0	-2946.808530	14.3	-2946.828597
30.0	-2946.858616	33.0	-2946.846212	29.0	-2946.828583	28.0	-2946.808530	14.4	-2946.828596
31.0	-2946.858616	34.0	-2946.846212	30.0	-2946.828583	29.0	-2946.808529	17.0	-2946.828588
32.0	-2946.858616	36.0	-2946.846212	31.0	-2946.828583	30.0	-2946.808529	19.0	-2946.828586
34.0	-2946.858616	37.0	-2946.846212	32.0	-2946.828582	31.0	-2946.808529	20.0	-2946.828585
36.0	-2946.858616	38.0	-2946.846212	33.0	-2946.828582	32.0	-2946.808529	21.0	-2946.828584
37.0	-2946.858616	39.0	-2946.846212	34.0	-2946.828582	33.0	-2946.808529	23.0	-2946.828584
38.0	-2946.858616	40.0	-2946.846212	36.0	-2946.828582	34.0	-2946.808528	24.0	-2946.828583
39.0	-2946.858616	42.0	-2946.846212	37.0	-2946.828582	36.0	-2946.808528	26.0	-2946.828583
40.0	-2946.858616	44.0	-2946.846212	38.0	-2946.828582	37.0	-2946.808528	27.0	-2946.828583
42.0	-2946.858616	46.0	-2946.846212	39.0	-2946.828582	38.0	-2946.808528	28.0	-2946.828583
44.0	-2946.858616	48.0	-2946.846212	40.0	-2946.828582	40.0	-2946.808528	29.0	-2946.828583
46.0	-2946.858616	52.0	-2946.846212	42.0	-2946.828582	42.0	-2946.808528	30.0	-2946.828583
48.0	-2946.858616	54.0	-2946.846212	44.0	-2946.828582	44.0	-2946.808528	31.0	-2946.828583
50.0	-2946.858616	56.0	-2946.846212	46.0	-2946.828582	46.0	-2946.808528	32.0	-2946.828583
52.0	-2946.858616	58.0	-2946.846212	48.0	-2946.828582	48.0	-2946.808528	33.0	-2946.828583
56.0	-2946.858616	60.0	-2946.846212	50.0	-2946.828582	50.0	-2946.808528	34.0	-2946.828582
58.0	-2946.858616	100.0	-2946.846212	54.0	-2946.828582	52.0	-2946.808528	36.0	-2946.828582
60.0	-2946.858616	150.0	-2946.846212	56.0	-2946.828582	54.0	-2946.808528	37.0	-2946.828582
100.0	-2946.858616	200.0	-2946.846212	58.0	-2946.828582	56.0	-2946.808528	38.0	-2946.828582
150.0	-2946.858616			60.0	-2946.828582	58.0	-2946.808528	39.0	-2946.828582
200.0	-2946.858616			100.0	-2946.828582	60.0	-2946.808528	40.0	-2946.828582
				150.0	-2946.828582	100.0	-2946.808528	42.0	-2946.828582
				200.0	-2946.828582	150.0	-2946.808528	44.0	-2946.828582
						200.0	-2946.808528	46.0	-2946.828582
								52.0	-2946.828582
								54.0	-2946.828582
								56.0	-2946.828582
								58.0	-2946.828582
								60.0	-2946.828582
								100.0	-2946.828582
								150.0	-2946.828582
								200.0	-2946.828582
