# Supplementary material for: Benchmark Study of the Electronic States of the LiRb Molecule: Ab Initio Calculations with the Fock Space Coupled Cluster Approach
Source: Molecules. 2023 Nov 17;28(22):7645. doi: 10.3390/molecules28227645 (PMC10675596; doi:10.3390/molecules28227645)
Supplement: Supplementary file 1 [file molecules-28-07645-s001.zip › lirb_unanorccplus_pi_delta_singlet_asymptotic.pdf]

#R[A]	1^1 pi	R[A]	2^1 pi	R[A]	3^1 pi	R[A]	4^1 pi	R[A]	1^1 delta
1.4	0.397088	1.4	0.422434	1.4	0.445844	1.4	0.471764	1.4	0.419988
1.5	0.301540	1.5	0.326098	1.6	0.276020	1.5	0.374611	1.5	0.321182
1.6	0.235497	1.6	0.259548	1.7	0.228198	1.6	0.307117	1.6	0.252667
1.7	0.190222	1.7	0.213453	1.8	0.195172	1.7	0.259945	1.7	0.205322
1.8	0.158676	1.8	0.180875	2.0	0.155750	1.8	0.226470	1.8	0.172290
1.9	0.136619	1.9	0.157253	2.1	0.143881	1.9	0.202086	1.9	0.149081
2.0	0.120851	2.1	0.126227	2.2	0.134901	2.0	0.183665	2.0	0.132514
2.1	0.109287	2.2	0.115304	2.3	0.127833	2.1	0.169013	2.2	0.111349
2.3	0.093296	2.3	0.106256	2.4	0.121985	2.2	0.156814	2.3	0.104302
2.4	0.087149	2.4	0.098635	2.5	0.116955	2.3	0.146306	2.4	0.098625
2.5	0.081575	2.5	0.092204	2.6	0.112496	2.4	0.137092	2.5	0.093901
2.6	0.076383	2.6	0.086787	2.7	0.108451	2.5	0.128984	2.6	0.089870
2.8	0.067205	2.7	0.082187	2.8	0.104740	2.6	0.121888	2.7	0.086376
2.9	0.063354	2.8	0.078227	2.9	0.101337	2.7	0.115734	2.8	0.083335
3.0	0.060043	2.9	0.074782	3.0	0.098243	2.8	0.110447	2.9	0.080700
3.05	0.058587	3.0	0.071779	3.05	0.096814	2.9	0.105941	3.0	0.078447
3.1	0.057261	3.05	0.070428	3.15	0.094195	3.0	0.102124	3.05	0.077459
3.15	0.056059	3.1	0.069173	3.2	0.093002	3.05	0.100448	3.1	0.076560
3.2	0.054977	3.2	0.066938	3.25	0.091884	3.1	0.098916	3.15	0.075748
3.25	0.054009	3.25	0.065951	3.3	0.090838	3.15	0.097518	3.2	0.075020
3.3	0.053148	3.3	0.065048	3.35	0.089861	3.2	0.096248	3.25	0.074374
3.35	0.052389	3.35	0.064224	3.45	0.088102	3.25	0.095100	3.3	0.073807
3.45	0.051148	3.45	0.062804	3.55	0.086613	3.3	0.094068	3.35	0.073316
3.55	0.050279	3.8	0.059875	3.8	0.083718	3.35	0.093146	3.45	0.072547
3.8	0.049061	3.85	0.059673	3.85	0.083283	3.45	0.091608	3.8	0.071713
3.85	0.048973	3.9	0.059517	3.9	0.082889	3.55	0.090452	3.85	0.071781
3.9	0.048921	4.0	0.059325	3.95	0.082533	3.8	0.088805	3.9	0.071885
3.95	0.048903	4.1	0.059290	4.0	0.082219	3.85	0.088657	3.95	0.072023
4.0	0.048914	4.2	0.059385	4.1	0.081680	3.9	0.088557	4.0	0.072120
4.1	0.049004	4.3	0.059590	4.2	0.081178	3.95	0.088501	4.1	0.072605
4.3	0.049365	4.4	0.059886	4.3	0.080873	4.0	0.088481	4.2	0.073037
4.4	0.049609	4.5	0.060254	4.4	0.080663	4.1	0.088551	4.3	0.073604
4.5	0.049876	4.6	0.060687	4.5	0.080574	4.2	0.088729	4.4	0.074220
4.6	0.050136	4.7	0.061150	4.6	0.080459	4.3	0.089010	4.5	0.074869
4.7	0.050414	4.75	0.061394	4.7	0.080471	4.4	0.089363	4.6	0.075538
4.75	0.050549	4.8	0.061642	4.75	0.080490	4.5	0.089774	4.7	0.076208
4.8	0.050682	4.9	0.062146	4.8	0.080521	4.6	0.090217	4.75	0.076543
4.85	0.050813	5.0	0.062650	4.85	0.080562	4.7	0.090705	4.8	0.076876
4.9	0.050943	5.1	0.063132	4.9	0.080613	4.75	0.090954	4.85	0.077206
5.0	0.051194	5.2	0.063619	5.0	0.080739	4.8	0.091208	4.9	0.077531
5.1	0.051443	5.3	0.064071	5.1	0.080891	4.8	0.091466	5.0	0.078166
5.3	0.051886	5.4	0.064494	5.2	0.081067	4.9	0.091727	5.1	0.078781
5.4	0.052095	5.5	0.064886	5.3	0.081256	5.0	0.092257	5.2	0.079351
5.5	0.052294	5.6	0.065249	5.4	0.081456	5.1	0.092755	5.3	0.079896
5.6	0.052449	5.7	0.065572	5.5	0.081662	5.2	0.093342	5.4	0.080404
5.7	0.052661	5.8	0.065866	5.6	0.081855	5.3	0.093895	5.5	0.080877
5.8	0.052830	5.9	0.066133	5.7	0.082076	5.4	0.094452	5.6	0.081314
5.9	0.052949	6.0	0.066363	5.8	0.082278	5.5	0.095010	5.7	0.081715
6.0	0.053141	6.2	0.066755	5.9	0.082466	5.6	0.095559	5.8	0.082082
6.2	0.053376	6.6	0.067282	6.0	0.082663	5.7	0.096141	6.0	0.082721
6.4	0.053626	6.8	0.067452	6.2	0.083010	5.8	0.096707	6.2	0.083245
6.6	0.053849	7.0	0.067583	6.4	0.083324	5.9	0.097247	6.4	0.083672
6.8	0.054091	7.4	0.067749	6.6	0.083600	6.0	0.097822	6.6	0.084019
7.0	0.054228	7.6	0.067801	6.8	0.083844	6.2	0.098870	6.8	0.084298
7.4	0.054557	7.8	0.067840	7.0	0.084052	6.4	0.099868	7.0	0.084524
7.6	0.054650	7.95	0.067862	7.4	0.084403	6.8	0.101578	7.4	0.084854
7.8	0.054781	8.0	0.067869	7.6	0.084540	7.0	0.102212	7.6	0.084974
7.95	0.054831	8.05	0.067875	7.8	0.084672	7.4	0.103235	7.8	0.085071
8.05	0.054876	8.2	0.067890	7.95	0.084751	7.6	0.103575	7.95	0.085132
8.2	0.054938	8.35	0.067904	8.0	0.084778	7.8	0.103889	8.0	0.085151
8.35	0.055008	8.4	0.067907	8.05	0.084804	8.0	0.104111	8.05	0.085168
8.4	0.055012	8.45	0.067910	8.2	0.084877	8.05	0.104164	8.2	0.085216
8.45	0.055029	8.5	0.067914	8.35	0.084946	8.2	0.104309	8.35	0.085257
8.5	0.055056	8.6	0.067919	8.4	0.084965	8.35	0.104445	8.4	0.085269
8.6	0.055076	8.8	0.067928	8.45	0.084985	8.4	0.104472	8.45	0.085281
8.8	0.055132	9.01	0.067936	8.5	0.085006	8.45	0.104508	8.5	0.085292
9.01	0.055190	9.2	0.067941	8.6	0.085042	8.5	0.104552	8.6	0.085313
9.2	0.055229	9.4	0.067946	8.8	0.085111	8.6	0.104608	8.8	0.085350
9.4	0.055267	9.6	0.067949	9.01	0.085170	8.8	0.104722	9.2	0.085406
9.6	0.055299	9.8	0.067951	9.4	0.085273	9.01	0.104828	9.6	0.085446
9.8	0.055327	10.0	0.067952	9.6	0.085311	9.2	0.104897	10.0	0.085476
10.0	0.055352	10.2	0.067954	9.8	0.085343	9.4	0.104969	10.2	0.085487
10.2	0.055373	10.4	0.067954	10.0	0.085370	9.6	0.105032	10.4	0.085497
10.4	0.055392	10.6	0.067955	10.2	0.085393	9.8	0.105087	10.6	0.085506
10.6	0.055408	10.8	0.067956	10.4	0.085412	10.0	0.105136	10.8	0.085514
10.8	0.055423	11.2	0.067956	10.6	0.085428	10.2	0.105179	11.2	0.085527
11.2	0.055446	11.4	0.067956	10.8	0.085442	10.6	0.105253	11.4	0.085533
11.4	0.055456	11.6	0.067955	11.2	0.085465	10.8	0.105285	11.6	0.085537
11.6	0.055466	11.8	0.067955	11.4	0.085473	11.2	0.105341	11.8	0.085542
11.8	0.055474	12.0	0.067955	11.6	0.085483	11.4	0.105365	12.0	0.085546
12.0	0.055481	12.2	0.067957	11.8	0.085490	11.6	0.105390	12.2	0.085549
12.2	0.055487	20.0	0.067955	12.0	0.085497	11.8	0.105410	12.4	0.085552
19.0	0.055545	21.0	0.067955	12.2	0.085501	12.0	0.105429	12.6	0.085555
20.0	0.055547	23.0	0.067955	19.0	0.085578	12.2	0.105445	12.8	0.085558
21.0	0.055548	24.0	0.067955	20.0	0.085580	19.0	0.105623	12.9	0.085559
23.0	0.055549	26.0	0.067955	21.0	0.085581	20.0	0.105627	13.0	0.085560
24.0	0.055549	27.0	0.067955	23.0	0.085583	21.0	0.105630	13.8	0.085567
26.0	0.055550	28.0	0.067955	24.0	0.085583	23.0	0.105633	13.9	0.085568
27.0	0.055550	29.0	0.067955	26.0	0.085584	24.0	0.105635	14.1	0.085569
28.0	0.055550	30.0	0.067955	27.0	0.085584	26.0	0.105636	14.2	0.085570
29.0	0.055551	32.0	0.067955	28.0	0.085584	27.0	0.105637	14.3	0.085570
30.0	0.055551	33.0	0.067955	29.0	0.085584	28.0	0.105637	14.4	0.085571
31.0	0.055551	34.0	0.067955	30.0	0.085584	29.0	0.105638	17.0	0.085579
32.0	0.055551	36.0	0.067955	31.0	0.085584	30.0	0.105638	19.0	0.085581
34.0	0.055551	37.0	0.067955	32.0	0.085585	31.0	0.105638	20.0	0.085582
36.0	0.055551	38.0	0.067955	33.0	0.085585	32.0	0.105638	21.0	0.085583
37.0	0.055551	39.0	0.067955	34.0	0.085585	33.0	0.105638	23.0	0.085583
38.0	0.055551	40.0	0.067955	36.0	0.085585	34.0	0.105639	24.0	0.085584
39.0	0.055551	42.0	0.067955	37.0	0.085585	36.0	0.105639	26.0	0.085584
40.0	0.055551	44.0	0.067955	38.0	0.085585	37.0	0.105639	27.0	0.085584
42.0	0.055551	46.0	0.067955	39.0	0.085585	38.0	0.105639	28.0	0.085584
44.0	0.055551	48.0	0.067955	40.0	0.085585	40.0	0.105639	29.0	0.085584
46.0	0.055551	52.0	0.067955	42.0	0.085585	42.0	0.105639	30.0	0.085584
48.0	0.055551	54.0	0.067955	44.0	0.085585	44.0	0.105639	31.0	0.085584
50.0	0.055551	56.0	0.067955	46.0	0.085585	46.0	0.105639	32.0	0.085584
52.0	0.055551	58.0	0.067955	48.0	0.085585	48.0	0.105639	33.0	0.085584
56.0	0.055551	60.0	0.067955	50.0	0.085585	50.0	0.105639	34.0	0.085585
58.0	0.055551	100.0	0.067955	54.0	0.085585	52.0	0.105639	36.0	0.085585
60.0	0.055551	150.0	0.067955	56.0	0.085585	54.0	0.105639	37.0	0.085585
100.0	0.055551	200.0	0.067955	58.0	0.085585	56.0	0.105639	38.0	0.085585
150.0	0.055551			60.0	0.085585	58.0	0.105639	39.0	0.085585
200.0	0.055551			100.0	0.085585	60.0	0.105639	40.0	0.085585
				150.0	0.085585	100.0	0.105639	42.0	0.085585
				200.0	0.085585	150.0	0.105639	44.0	0.085585
						200.0	0.105639	46.0	0.085585
								52.0	0.085585
								54.0	0.085585
								56.0	0.085585
								58.0	0.085585
								60.0	0.085585
								100.0	0.085585
								150.0	0.085585
								200.0	0.085585
