# Supplementary material for: Benchmark Study of the Electronic States of the LiRb Molecule: Ab Initio Calculations with the Fock Space Coupled Cluster Approach
Source: Molecules. 2023 Nov 17;28(22):7645. doi: 10.3390/molecules28227645 (PMC10675596; doi:10.3390/molecules28227645)
Supplement: Supplementary file 1 [file molecules-28-07645-s001.zip › lirb_unanorccplus_pi_delta_triplet.pdf]

#R[A]	1^3 pi	R[A]	2^3 pi	R[A]	3^3 pi	R[A]	4^3 pi	R[A]	1^3 delta
1.4	-2946.552051	1.4	-2946.502690	1.4	-2946.470601	1.4	-2946.439697	1.4	-2946.484458
1.5	-2946.648738	1.7	-2946.706737	1.5	-2946.571114	1.5	-2946.536515	1.5	-2946.584560
1.6	-2946.715783	1.8	-2946.737760	1.7	-2946.688641	1.6	-2946.603992	1.6	-2946.653649
1.7	-2946.761876	2.0	-2946.775611	1.8	-2946.722053	1.7	-2946.651645	1.7	-2946.701271
1.8	-2946.794352	2.1	-2946.787608	1.9	-2946.745603	1.8	-2946.684547	1.8	-2946.734122
1.9	-2946.817470	2.2	-2946.797132	2.0	-2946.762405	1.9	-2946.709062	1.9	-2946.756871
2.0	-2946.834355	2.3	-2946.805114	2.1	-2946.774735	2.0	-2946.727814	2.0	-2946.772713
2.1	-2946.847072	2.4	-2946.812121	2.2	-2946.784032	2.1	-2946.742820	2.1	-2946.783836
2.2	-2946.856978	2.6	-2946.824107	2.4	-2946.796868	2.2	-2946.755388	2.3	-2946.797435
2.3	-2946.864940	2.8	-2946.833797	2.5	-2946.801480	2.3	-2946.766298	2.5	-2946.804861
2.4	-2946.871500	2.9	-2946.837818	2.7	-2946.808614	2.4	-2946.775965	2.6	-2946.810641
2.5	-2946.876973	3.0	-2946.841307	2.8	-2946.811497	2.5	-2946.784579	2.7	-2946.815791
2.6	-2946.881549	3.05	-2946.842860	2.9	-2946.814103	2.6	-2946.792216	2.8	-2946.820329
2.7	-2946.885345	3.1	-2946.844290	3.0	-2946.816567	2.7	-2946.798911	2.9	-2946.824272
2.8	-2946.888445	3.15	-2946.845603	3.05	-2946.817793	2.8	-2946.804690	3.0	-2946.827640
2.9	-2946.890913	3.2	-2946.846804	3.1	-2946.819022	2.9	-2946.809570	3.0	-2946.829118
3.0	-2946.892810	3.25	-2946.847898	3.15	-2946.820237	3.0	-2946.813544	3.05	-2946.830466
3.05	-2946.893562	3.3	-2946.848890	3.2	-2946.821403	3.05	-2946.815181	3.1	-2946.831689
3.1	-2946.894192	3.35	-2946.849785	3.25	-2946.822494	3.1	-2946.816595	3.15	-2946.832792
3.15	-2946.894707	3.45	-2946.851311	3.3	-2946.823494	3.15	-2946.817818	3.2	-2946.833781
3.2	-2946.895113	3.55	-2946.852505	3.35	-2946.824394	3.2	-2946.818895	3.3	-2946.834662
3.25	-2946.895418	3.8	-2946.854403	3.45	-2946.825898	3.25	-2946.819866	3.35	-2946.835441
3.3	-2946.895627	3.85	-2946.854623	3.55	-2946.827040	3.3	-2946.820759	3.45	-2946.836718
3.35	-2946.895746	3.9	-2946.854799	3.8	-2946.828601	3.35	-2946.821591	3.85	-2946.838905
3.45	-2946.895739	3.95	-2946.854937	3.85	-2946.828810	3.45	-2946.823114	3.9	-2946.838934
3.55	-2946.895310	4.0	-2946.855042	3.9	-2946.829063	3.55	-2946.824383	3.95	-2946.838924
3.8	-2946.893691	4.1	-2946.855150	3.95	-2946.829385	3.8	-2946.827301	4.0	-2946.838946
3.85	-2946.893203	4.2	-2946.855146	4.0	-2946.829755	3.85	-2946.827700	4.1	-2946.838688
3.9	-2946.892679	4.3	-2946.855062	4.1	-2946.830534	3.9	-2946.827981	4.2	-2946.838470
3.95	-2946.892121	4.4	-2946.854908	4.2	-2946.831304	3.95	-2946.828123	4.3	-2946.838101
4.0	-2946.891547	4.5	-2946.854699	4.3	-2946.832009	4.0	-2946.828154	4.4	-2946.837673
4.1	-2946.890293	4.6	-2946.854451	4.4	-2946.832657	4.1	-2946.827993	4.5	-2946.837204
4.2	-2946.888952	4.7	-2946.854163	4.5	-2946.833244	4.2	-2946.827668	4.6	-2946.836707
4.3	-2946.887551	4.75	-2946.854013	4.6	-2946.833781	4.3	-2946.827199	4.7	-2946.836199
4.4	-2946.886105	4.8	-2946.853859	4.75	-2946.834500	4.4	-2946.826641	4.75	-2946.835942
4.5	-2946.884635	4.85	-2946.853701	4.8	-2946.834713	4.5	-2946.826011	4.8	-2946.835686
4.6	-2946.883133	4.9	-2946.853540	4.85	-2946.834915	4.6	-2946.825356	4.85	-2946.835431
4.7	-2946.881645	5.0	-2946.853212	4.9	-2946.835105	4.7	-2946.824682	4.9	-2946.835179
4.75	-2946.880903	5.2	-2946.852540	5.0	-2946.835453	4.75	-2946.824343	5.0	-2946.834684
4.8	-2946.880165	5.3	-2946.852202	5.1	-2946.835761	4.8	-2946.824007	5.1	-2946.834205
4.85	-2946.879432	5.4	-2946.851863	5.2	-2946.836025	4.85	-2946.823677	5.2	-2946.833754
4.9	-2946.878704	5.5	-2946.851525	5.3	-2946.836252	4.9	-2946.823354	5.3	-2946.833325
5.0	-2946.877273	5.6	-2946.851218	5.4	-2946.836440	5.0	-2946.822742	5.4	-2946.832922
5.1	-2946.875850	5.7	-2946.850850	5.5	-2946.836594	5.1	-2946.822180	5.5	-2946.832546
5.2	-2946.874530	5.8	-2946.850514	5.6	-2946.836707	5.2	-2946.821700	5.6	-2946.832198
5.3	-2946.873232	5.9	-2946.850216	5.7	-2946.836786	5.4	-2946.820950	5.7	-2946.831877
5.4	-2946.871991	6.0	-2946.849854	5.8	-2946.836826	5.5	-2946.820684	5.8	-2946.831582
5.5	-2946.870812	6.2	-2946.849250	5.9	-2946.836829	5.6	-2946.820536	5.9	-2946.831311
5.6	-2946.869685	6.4	-2946.848659	6.0	-2946.836795	5.7	-2946.820316	6.0	-2946.831064
5.7	-2946.868654	6.6	-2946.848134	6.2	-2946.836612	5.8	-2946.820198	6.2	-2946.830635
5.8	-2946.867681	6.8	-2946.847674	6.4	-2946.836281	6.0	-2946.820049	6.4	-2946.830280
5.9	-2946.866774	7.0	-2946.847330	6.6	-2946.835813	6.2	-2946.820020	6.6	-2946.829989
6.0	-2946.865952	7.4	-2946.846824	6.8	-2946.835231	6.4	-2946.819960	6.8	-2946.829751
6.2	-2946.864511	7.6	-2946.846669	7.0	-2946.834575	6.6	-2946.819892	7.0	-2946.829556
6.4	-2946.863338	7.95	-2946.846480	7.4	-2946.833179	7.0	-2946.819641	7.4	-2946.829266
6.6	-2946.862397	8.0	-2946.846460	7.6	-2946.832511	7.4	-2946.819107	7.6	-2946.829159
6.8	-2946.861635	8.05	-2946.846441	7.8	-2946.831896	7.8	-2946.818315	7.8	-2946.829070
7.0	-2946.861065	8.2	-2946.846394	7.95	-2946.831476	7.95	-2946.817987	7.95	-2946.829015
7.4	-2946.860224	8.35	-2946.846354	8.0	-2946.831345	8.0	-2946.817854	8.0	-2946.828998
7.6	-2946.859954	8.4	-2946.846346	8.05	-2946.831219	8.05	-2946.817718	8.05	-2946.828982
7.8	-2946.859713	8.45	-2946.846336	8.2	-2946.830867	8.2	-2946.817288	8.2	-2946.828938
7.95	-2946.859585	8.5	-2946.846324	8.35	-2946.830562	8.35	-2946.816786	8.35	-2946.828900
8.0	-2946.859543	8.6	-2946.846310	8.4	-2946.830461	8.4	-2946.816673	8.4	-2946.828888
8.2	-2946.859396	8.8	-2946.846284	8.45	-2946.830370	8.45	-2946.816512	8.45	-2946.828877
8.35	-2946.859297	9.01	-2946.846262	8.5	-2946.830291	8.5	-2946.816305	8.5	-2946.828867
8.4	-2946.859276	9.2	-2946.846248	8.6	-2946.830122	8.6	-2946.816020	8.6	-2946.828847
8.45	-2946.859250	9.4	-2946.846238	8.8	-2946.829841	8.8	-2946.815346	8.8	-2946.828812
8.5	-2946.859219	9.6	-2946.846230	9.01	-2946.829612	9.01	-2946.814582	9.2	-2946.828758
8.6	-2946.859178	9.8	-2946.846225	9.2	-2946.829433	9.4	-2946.813237	9.4	-2946.828737
8.8	-2946.859097	10.0	-2946.846221	9.4	-2946.829280	9.6	-2946.812606	9.6	-2946.828719
9.01	-2946.859026	10.2	-2946.846218	9.6	-2946.829157	9.8	-2946.812013	9.8	-2946.828704
9.2	-2946.858977	10.4	-2946.846216	9.8	-2946.829058	10.0	-2946.811469	10.0	-2946.828691
9.4	-2946.858930	10.6	-2946.846214	10.0	-2946.828979	10.2	-2946.810981	10.2	-2946.828679
9.6	-2946.858889	11.2	-2946.846212	10.2	-2946.828916	10.6	-2946.810195	10.4	-2946.828669
9.8	-2946.858856	11.4	-2946.846211	10.8	-2946.828791	10.8	-2946.809896	10.6	-2946.828660
10.2	-2946.858802	11.6	-2946.846212	11.2	-2946.828743	11.2	-2946.809458	11.2	-2946.828640
10.4	-2946.858782	11.8	-2946.846212	11.4	-2946.828725	11.4	-2946.809301	11.4	-2946.828634
10.6	-2946.858764	12.0	-2946.846212	11.6	-2946.828707	11.6	-2946.809212	11.6	-2946.828629
11.2	-2946.858723	12.2	-2946.846211	11.8	-2946.828695	11.8	-2946.809107	11.8	-2946.828625
11.4	-2946.858712	20.0	-2946.846212	12.0	-2946.828684	12.0	-2946.809022	12.2	-2946.828618
11.6	-2946.858701	21.0	-2946.846212	12.2	-2946.828678	12.2	-2946.808926	12.4	-2946.828615
11.8	-2946.858694	23.0	-2946.846212	19.0	-2946.828589	19.0	-2946.808544	12.6	-2946.828612
12.0	-2946.858687	24.0	-2946.846212	20.0	-2946.828587	20.0	-2946.808540	12.8	-2946.828609
12.2	-2946.858681	26.0	-2946.846212	21.0	-2946.828586	21.0	-2946.808537	12.9	-2946.828608
19.0	-2946.858622	27.0	-2946.846212	23.0	-2946.828584	23.0	-2946.808534	13.0	-2946.828607
20.0	-2946.858620	28.0	-2946.846212	24.0	-2946.828584	24.0	-2946.808532	13.8	-2946.828600
21.0	-2946.858619	29.0	-2946.846212	26.0	-2946.828583	26.0	-2946.808531	13.9	-2946.828599
23.0	-2946.858618	30.0	-2946.846212	27.0	-2946.828583	28.0	-2946.808530	14.1	-2946.828598
24.0	-2946.858618	32.0	-2946.846212	28.0	-2946.828583	29.0	-2946.808529	14.2	-2946.828597
26.0	-2946.858617	33.0	-2946.846212	29.0	-2946.828583	30.0	-2946.808529	14.3	-2946.828597
27.0	-2946.858617	34.0	-2946.846212	30.0	-2946.828583	31.0	-2946.808529	14.4	-2946.828596
28.0	-2946.858617	36.0	-2946.846212	31.0	-2946.828583	32.0	-2946.808529	17.0	-2946.828588
29.0	-2946.858616	37.0	-2946.846212	32.0	-2946.828582	33.0	-2946.808529	19.0	-2946.828586
30.0	-2946.858616	38.0	-2946.846212	33.0	-2946.828582	34.0	-2946.808528	20.0	-2946.828585
31.0	-2946.858616	39.0	-2946.846212	34.0	-2946.828582	36.0	-2946.808528	23.0	-2946.828584
32.0	-2946.858616	40.0	-2946.846212	36.0	-2946.828582	37.0	-2946.808528	24.0	-2946.828583
34.0	-2946.858616	42.0	-2946.846212	37.0	-2946.828582	38.0	-2946.808528	26.0	-2946.828583
36.0	-2946.858616	44.0	-2946.846212	38.0	-2946.828582	40.0	-2946.808528	27.0	-2946.828583
37.0	-2946.858616	46.0	-2946.846212	39.0	-2946.828582	42.0	-2946.808528	28.0	-2946.828583
38.0	-2946.858616	48.0	-2946.846212	40.0	-2946.828582	44.0	-2946.808528	29.0	-2946.828583
39.0	-2946.858616	52.0	-2946.846212	42.0	-2946.828582	46.0	-2946.808528	30.0	-2946.828583
40.0	-2946.858616	54.0	-2946.846212	44.0	-2946.828582	48.0	-2946.808528	31.0	-2946.828583
42.0	-2946.858616	56.0	-2946.846212	46.0	-2946.828582	50.0	-2946.808528	32.0	-2946.828583
44.0	-2946.858616	58.0	-2946.846212	48.0	-2946.828582	52.0	-2946.808528	33.0	-2946.828583
46.0	-2946.858616	60.0	-2946.846212	50.0	-2946.828582	54.0	-2946.808528	34.0	-2946.828582
48.0	-2946.858616	100.0	-2946.846212	54.0	-2946.828582	56.0	-2946.808528	37.0	-2946.828582
50.0	-2946.858616	150.0	-2946.846212	56.0	-2946.828582	58.0	-2946.808528	38.0	-2946.828582
52.0	-2946.858616	200.0	-2946.846212	58.0	-2946.828582	60.0	-2946.808528	39.0	-2946.828582
56.0	-2946.858616			60.0	-2946.828582	100.0	-2946.808528	40.0	-2946.828582
58.0	-2946.858616			100.0	-2946.828582	150.0	-2946.808528	42.0	-2946.828582
60.0	-2946.858616			150.0	-2946.828582	200.0	-2946.808528	44.0	-2946.828582
100.0	-2946.858616			200.0	-2946.828582			48.0	-2946.828582
150.0	-2946.858616							50.0	-2946.828582
200.0	-2946.858616							52.0	-2946.828582
								54.0	-2946.828582
								56.0	-2946.828582
								58.0	-2946.828582
								60.0	-2946.828582
								100.0	-2946.828582
								150.0	-2946.828582
								200.0	-2946.828582
