# Supplementary material for: Benchmark Study of the Electronic States of the LiRb Molecule: Ab Initio Calculations with the Fock Space Coupled Cluster Approach
Source: Molecules. 2023 Nov 17;28(22):7645. doi: 10.3390/molecules28227645 (PMC10675596; doi:10.3390/molecules28227645)
Supplement: Supplementary file 1 [file molecules-28-07645-s001.zip › lirb_unanorccplus_pi_delta_triplet_asymptotic.pdf]

#R[A]	1^3pi	R[A]	2^3pi	R[A]	3^3pi	R[A]	4^3pi	R[A]	1^3delta
1.4	0.362116	1.4	0.411477	1.4	0.443566	1.4	0.474470	1.4	0.429709
1.5	0.265429	1.7	0.207430	1.5	0.343053	1.5	0.377652	1.5	0.329607
1.6	0.198384	1.8	0.176407	1.7	0.225526	1.6	0.310175	1.6	0.260518
1.7	0.152291	2.0	0.138556	1.8	0.192114	1.7	0.262522	1.7	0.212896
1.8	0.119815	2.1	0.126559	1.9	0.168564	1.8	0.229620	1.8	0.180045
1.9	0.096697	2.2	0.117035	2.0	0.151762	1.9	0.205105	1.9	0.157296
2.0	0.079812	2.3	0.109053	2.1	0.139432	2.0	0.186353	2.0	0.141454
2.1	0.067095	2.4	0.102046	2.2	0.130135	2.1	0.171347	2.1	0.130331
2.2	0.057189	2.6	0.090060	2.4	0.117299	2.2	0.158779	2.3	0.116732
2.3	0.049227	2.8	0.080370	2.5	0.112687	2.3	0.147869	2.5	0.109306
2.4	0.042667	2.9	0.076349	2.7	0.105553	2.4	0.138202	2.6	0.103526
2.5	0.037194	3.0	0.072860	2.8	0.102670	2.5	0.129588	2.7	0.098376
2.6	0.032618	3.05	0.071307	2.9	0.100064	2.6	0.121951	2.8	0.093838
2.7	0.028822	3.1	0.069877	3.0	0.097600	2.7	0.115256	2.9	0.089895
2.8	0.025722	3.15	0.068564	3.05	0.096374	2.8	0.109477	3.0	0.086527
2.9	0.023254	3.2	0.067363	3.1	0.095145	2.9	0.104597	3.0	0.085049
3.0	0.021357	3.25	0.066269	3.15	0.093930	3.0	0.100623	3.05	0.083701
3.05	0.020605	3.3	0.065277	3.2	0.092764	3.05	0.098986	3.1	0.082478
3.1	0.019975	3.35	0.064382	3.25	0.091673	3.1	0.097572	3.15	0.081375
3.15	0.019460	3.45	0.062856	3.3	0.090673	3.15	0.096349	3.2	0.080386
3.2	0.019054	3.55	0.061662	3.35	0.089773	3.2	0.095272	3.3	0.079505
3.25	0.018749	3.8	0.059764	3.45	0.088269	3.25	0.094301	3.35	0.078726
3.3	0.018540	3.85	0.059544	3.55	0.087127	3.3	0.093408	3.45	0.077449
3.35	0.018421	3.9	0.059368	3.8	0.085566	3.35	0.092576	3.85	0.075262
3.45	0.018428	3.95	0.059230	3.85	0.085357	3.45	0.091053	3.9	0.075233
3.55	0.018857	4.0	0.059125	3.9	0.085104	3.55	0.089784	3.95	0.075243
3.8	0.020476	4.1	0.059017	3.95	0.084782	3.8	0.086866	4.0	0.075221
3.85	0.020964	4.2	0.059021	4.0	0.084412	3.85	0.086467	4.1	0.075479
3.9	0.021488	4.3	0.059105	4.1	0.083633	3.9	0.086186	4.2	0.075697
3.95	0.022046	4.4	0.059259	4.2	0.082863	3.95	0.086044	4.3	0.076066
4.0	0.022620	4.5	0.059468	4.3	0.082158	4.0	0.086013	4.4	0.076494
4.1	0.023874	4.6	0.059716	4.4	0.081510	4.1	0.086174	4.5	0.076963
4.2	0.025215	4.7	0.060004	4.5	0.080923	4.2	0.086499	4.6	0.077460
4.3	0.026616	4.75	0.060154	4.6	0.080386	4.3	0.086968	4.7	0.077968
4.4	0.028062	4.8	0.060308	4.75	0.079667	4.4	0.087526	4.75	0.078225
4.5	0.029532	4.85	0.060466	4.8	0.079454	4.5	0.088156	4.8	0.078481
4.6	0.031034	4.9	0.060627	4.85	0.079252	4.6	0.088811	4.85	0.078736
4.7	0.032522	5.0	0.060955	4.9	0.079062	4.7	0.089485	4.9	0.078988
4.75	0.033264	5.2	0.061627	5.0	0.078714	4.75	0.089824	5.0	0.079483
4.8	0.034002	5.3	0.061965	5.1	0.078406	4.8	0.090160	5.1	0.079962
4.85	0.034735	5.4	0.062304	5.2	0.078142	4.85	0.090490	5.2	0.080413
4.9	0.035463	5.5	0.062642	5.3	0.077915	4.9	0.090813	5.3	0.080842
5.0	0.036894	5.6	0.062949	5.4	0.077727	5.0	0.091425	5.4	0.081245
5.1	0.038317	5.7	0.063317	5.5	0.077573	5.1	0.091987	5.5	0.081621
5.2	0.039637	5.8	0.063653	5.6	0.077460	5.2	0.092467	5.6	0.081969
5.3	0.040935	5.9	0.063951	5.7	0.077381	5.4	0.093217	5.7	0.082290
5.4	0.042176	6.0	0.064313	5.8	0.077341	5.5	0.093483	5.8	0.082585
5.5	0.043355	6.2	0.064917	5.9	0.077338	5.6	0.093631	5.9	0.082856
5.6	0.044482	6.4	0.065508	6.0	0.077372	5.7	0.093851	6.0	0.083103
5.7	0.045513	6.6	0.066033	6.2	0.077555	5.8	0.093969	6.2	0.083532
5.8	0.046486	6.8	0.066493	6.4	0.077886	6.0	0.094118	6.4	0.083887
5.9	0.047393	7.0	0.066837	6.6	0.078354	6.2	0.094147	6.6	0.084178
6.0	0.048215	7.4	0.067343	6.8	0.078936	6.4	0.094207	6.8	0.084416
6.2	0.049656	7.6	0.067498	7.0	0.079592	6.6	0.094275	7.0	0.084611
6.4	0.050829	7.95	0.067687	7.4	0.080988	7.0	0.094526	7.4	0.084901
6.6	0.051770	8.0	0.067707	7.6	0.081656	7.4	0.095060	7.6	0.085008
6.8	0.052532	8.05	0.067726	7.8	0.082271	7.8	0.095852	7.8	0.085097
7.0	0.053102	8.2	0.067773	7.95	0.082691	7.95	0.096180	7.95	0.085152
7.4	0.053943	8.35	0.067813	8.0	0.082822	8.0	0.096313	8.0	0.085169
7.6	0.054213	8.4	0.067821	8.05	0.082948	8.05	0.096449	8.05	0.085185
7.8	0.054454	8.45	0.067831	8.2	0.083300	8.2	0.096879	8.2	0.085229
7.95	0.054582	8.5	0.067843	8.35	0.083605	8.35	0.097381	8.35	0.085267
8.0	0.054624	8.6	0.067857	8.4	0.083706	8.4	0.097494	8.4	0.085279
8.2	0.054771	8.8	0.067883	8.45	0.083797	8.45	0.097655	8.45	0.085290
8.35	0.054870	9.01	0.067905	8.5	0.083876	8.5	0.097862	8.5	0.085300
8.4	0.054891	9.2	0.067919	8.6	0.084045	8.6	0.098147	8.6	0.085320
8.45	0.054917	9.4	0.067929	8.8	0.084326	8.8	0.098821	8.8	0.085355
8.5	0.054948	9.6	0.067937	9.01	0.084555	9.01	0.099585	9.2	0.085409
8.6	0.054989	9.8	0.067942	9.2	0.084734	9.4	0.100930	9.4	0.085430
8.8	0.055070	10.0	0.067946	9.4	0.084887	9.6	0.101561	9.6	0.085448
9.01	0.055141	10.2	0.067949	9.6	0.085010	9.8	0.102154	9.8	0.085463
9.2	0.055190	10.4	0.067951	9.8	0.085109	10.0	0.102698	10.0	0.085476
9.4	0.055237	10.6	0.067953	10.0	0.085188	10.2	0.103186	10.2	0.085488
9.6	0.055278	11.2	0.067955	10.2	0.085251	10.6	0.103972	10.4	0.085498
9.8	0.055311	11.4	0.067956	10.8	0.085376	10.8	0.104271	10.6	0.085507
10.2	0.055365	11.6	0.067955	11.2	0.085424	11.2	0.104709	11.2	0.085527
10.4	0.055385	11.8	0.067955	11.4	0.085442	11.4	0.104866	11.4	0.085533
10.6	0.055403	12.0	0.067955	11.6	0.085460	11.6	0.104955	11.6	0.085538
11.2	0.055444	12.2	0.067956	11.8	0.085472	11.8	0.105060	11.8	0.085542
11.4	0.055455	20.0	0.067955	12.0	0.085483	12.0	0.105145	12.2	0.085549
11.6	0.055466	21.0	0.067955	12.2	0.085489	12.2	0.105241	12.4	0.085552
11.8	0.055473	23.0	0.067955	19.0	0.085578	19.0	0.105623	12.6	0.085555
12.0	0.055480	24.0	0.067955	20.0	0.085580	20.0	0.105627	12.8	0.085558
12.2	0.055486	26.0	0.067955	21.0	0.085581	21.0	0.105630	12.9	0.085559
19.0	0.055545	27.0	0.067955	23.0	0.085583	23.0	0.105633	13.0	0.085560
20.0	0.055547	28.0	0.067955	24.0	0.085583	24.0	0.105635	13.8	0.085567
21.0	0.055548	29.0	0.067955	26.0	0.085584	26.0	0.105636	13.9	0.085568
23.0	0.055549	30.0	0.067955	27.0	0.085584	28.0	0.105637	14.1	0.085569
24.0	0.055549	32.0	0.067955	28.0	0.085584	29.0	0.105638	14.2	0.085570
26.0	0.055550	33.0	0.067955	29.0	0.085584	30.0	0.105638	14.3	0.085570
27.0	0.055550	34.0	0.067955	30.0	0.085584	31.0	0.105638	14.4	0.085571
28.0	0.055550	36.0	0.067955	31.0	0.085584	32.0	0.105638	17.0	0.085579
29.0	0.055551	37.0	0.067955	32.0	0.085585	33.0	0.105638	19.0	0.085581
30.0	0.055551	38.0	0.067955	33.0	0.085585	34.0	0.105639	20.0	0.085582
31.0	0.055551	39.0	0.067955	34.0	0.085585	36.0	0.105639	23.0	0.085583
32.0	0.055551	40.0	0.067955	36.0	0.085585	37.0	0.105639	24.0	0.085584
34.0	0.055551	42.0	0.067955	37.0	0.085585	38.0	0.105639	26.0	0.085584
36.0	0.055551	44.0	0.067955	38.0	0.085585	40.0	0.105639	27.0	0.085584
37.0	0.055551	46.0	0.067955	39.0	0.085585	42.0	0.105639	28.0	0.085584
38.0	0.055551	48.0	0.067955	40.0	0.085585	44.0	0.105639	29.0	0.085584
39.0	0.055551	52.0	0.067955	42.0	0.085585	46.0	0.105639	30.0	0.085584
40.0	0.055551	54.0	0.067955	44.0	0.085585	48.0	0.105639	31.0	0.085584
42.0	0.055551	56.0	0.067955	46.0	0.085585	50.0	0.105639	32.0	0.085584
44.0	0.055551	58.0	0.067955	48.0	0.085585	52.0	0.105639	33.0	0.085584
46.0	0.055551	60.0	0.067955	50.0	0.085585	54.0	0.105639	34.0	0.085585
48.0	0.055551	100.0	0.067955	54.0	0.085585	56.0	0.105639	37.0	0.085585
50.0	0.055551	150.0	0.067955	56.0	0.085585	58.0	0.105639	38.0	0.085585
52.0	0.055551	200.0	0.067955	58.0	0.085585	60.0	0.105639	39.0	0.085585
56.0	0.055551			60.0	0.085585	100.0	0.105639	40.0	0.085585
58.0	0.055551			100.0	0.085585	150.0	0.105639	42.0	0.085585
60.0	0.055551			150.0	0.085585	200.0	0.105639	44.0	0.085585
100.0	0.055551			200.0	0.085585			48.0	0.085585
150.0	0.055551							50.0	0.085585
200.0	0.055551							52.0	0.085585
								54.0	0.085585
								56.0	0.085585
								58.0	0.085585
								60.0	0.085585
								100.0	0.085585
								150.0	0.085585
								200.0	0.085585
