# Supplementary material for: Benchmark Study of the Electronic States of the LiRb Molecule: Ab Initio Calculations with the Fock Space Coupled Cluster Approach
Source: Molecules. 2023 Nov 17;28(22):7645. doi: 10.3390/molecules28227645 (PMC10675596; doi:10.3390/molecules28227645)
Supplement: Supplementary file 1 [file molecules-28-07645-s001.zip › lirb_unanorccplus_sigma_plus_singlet.pdf]

#R[A]	X^1 sigma^+	R[A]	2^1 sigma^+	R[A]	3^1 sigma^+	R[A]	4^1 sigma^+	R[A]	5^1 sigma^+	R[A]	6^1 sigma^+
1.4	-2946.587685	1.5	-2946.607439	1.4	-2946.494179	1.4	-2946.458037	1.4	-2946.454911	1.4	-2946.454020
1.5	-2946.681700	1.6	-2946.673568	1.5	-2946.592985	1.5	-2946.555225	1.5	-2946.554614	1.5	-2946.551215
1.6	-2946.746891	1.7	-2946.719233	1.6	-2946.661500	1.6	-2946.624590	1.6	-2946.622086	1.6	-2946.618223
1.7	-2946.791723	1.8	-2946.751134	1.7	-2946.708845	1.7	-2946.672800	1.7	-2946.668036	1.7	-2946.664723
1.8	-2946.823674	1.9	-2946.774196	1.8	-2946.741877	1.8	-2946.706569	1.8	-2946.699939	1.8	-2946.697905
1.9	-2946.846875	2.0	-2946.791193	2.0	-2946.781653	1.9	-2946.730907	2.0	-2946.740149	1.9	-2946.722006
2.0	-2946.864355	2.1	-2946.804517	2.1	-2946.793736	2.0	-2946.749207	2.1	-2946.754360	2.1	-2946.750908
2.1	-2946.878105	2.2	-2946.815604	2.2	-2946.802818	2.1	-2946.763645	2.2	-2946.766017	2.2	-2946.760650
2.2	-2946.889357	2.3	-2946.825278	2.3	-2946.809865	2.2	-2946.775606	2.3	-2946.776004	2.3	-2946.768812
2.3	-2946.898864	2.4	-2946.834038	2.4	-2946.815542	2.3	-2946.785826	2.4	-2946.784819	2.4	-2946.775964
2.4	-2946.907042	2.5	-2946.842067	2.5	-2946.820266	2.4	-2946.794674	2.5	-2946.792695	2.5	-2946.782388
2.5	-2946.914109	2.6	-2946.849396	2.6	-2946.824297	2.5	-2946.802377	2.6	-2946.799726	2.6	-2946.789539
2.6	-2946.920181	2.7	-2946.856008	2.7	-2946.830490	2.6	-2946.809099	2.7	-2946.805965	2.7	-2946.796144
2.7	-2946.925329	2.8	-2946.861888	2.8	-2946.835996	2.7	-2946.814965	2.8	-2946.811461	2.8	-2946.801949
2.8	-2946.929615	2.9	-2946.867041	2.9	-2946.840826	2.8	-2946.820059	2.9	-2946.816275	3.0	-2946.811307
2.9	-2946.933100	3.0	-2946.871498	3.0	-2946.845026	2.9	-2946.824444	3.0	-2946.820471	3.05	-2946.813214
3.0	-2946.935855	3.05	-2946.873479	3.05	-2946.846905	3.0	-2946.828172	3.05	-2946.822358	3.1	-2946.814962
3.05	-2946.936980	3.1	-2946.875303	3.1	-2946.848644	3.05	-2946.829804	3.1	-2946.824114	3.15	-2946.816560
3.1	-2946.937950	3.15	-2946.876979	3.15	-2946.850250	3.1	-2946.831292	3.15	-2946.825748	3.2	-2946.818015
3.15	-2946.938774	3.2	-2946.878513	3.2	-2946.851729	3.15	-2946.832641	3.2	-2946.827266	3.25	-2946.819335
3.2	-2946.939461	3.25	-2946.879913	3.25	-2946.853087	3.2	-2946.833858	3.25	-2946.828676	3.3	-2946.820529
3.25	-2946.940020	3.3	-2946.881186	3.3	-2946.854330	3.25	-2946.834949	3.3	-2946.829984	3.35	-2946.821603
3.3	-2946.940459	3.45	-2946.884321	3.35	-2946.855464	3.3	-2946.835922	3.35	-2946.831195	3.45	-2946.823422
3.35	-2946.940787	3.55	-2946.885666	3.45	-2946.857431	3.35	-2946.836783	3.45	-2946.833353	3.55	-2946.824842
3.45	-2946.941142	3.8	-2946.888484	3.55	-2946.858807	3.45	-2946.838199	3.8	-2946.838509	3.8	-2946.827021
3.55	-2946.941496	3.85	-2946.888802	3.8	-2946.861732	3.55	-2946.839199	3.85	-2946.838930	3.85	-2946.827259
3.8	-2946.939959	3.9	-2946.889067	3.85	-2946.862083	3.8	-2946.840748	3.95	-2946.839445	3.9	-2946.827441
3.85	-2946.939564	3.95	-2946.889281	3.9	-2946.862382	3.85	-2946.840950	4.0	-2946.839535	3.95	-2946.827573
3.9	-2946.939129	4.0	-2946.889475	3.95	-2946.862630	3.9	-2946.841165	4.1	-2946.839462	4.0	-2946.827657
3.95	-2946.938658	4.1	-2946.889683	4.0	-2946.862854	3.95	-2946.841408	4.2	-2946.839158	4.1	-2946.827700
4.0	-2946.938167	4.2	-2946.889748	4.1	-2946.863133	4.0	-2946.841717	4.3	-2946.838705	4.2	-2946.827597
4.1	-2946.937082	4.3	-2946.889693	4.2	-2946.863261	4.1	-2946.842292	4.4	-2946.838153	4.3	-2946.827372
4.2	-2946.935915	4.4	-2946.889536	4.3	-2946.863261	4.2	-2946.842851	4.5	-2946.837531	4.4	-2946.827045
4.3	-2946.934696	4.5	-2946.889294	4.6	-2946.862475	4.3	-2946.843352	4.6	-2946.836830	4.5	-2946.826635
4.4	-2946.933444	4.6	-2946.888997	4.7	-2946.862264	4.4	-2946.843782	4.7	-2946.836166	4.6	-2946.826163
4.5	-2946.932180	4.7	-2946.888590	4.75	-2946.862057	4.5	-2946.844143	4.75	-2946.835815	4.7	-2946.825625
4.6	-2946.930946	4.75	-2946.888382	4.8	-2946.861836	4.6	-2946.844460	4.8	-2946.835463	4.75	-2946.825341
4.7	-2946.929676	4.8	-2946.888161	4.85	-2946.861601	4.7	-2946.844687	4.9	-2946.834763	4.8	-2946.825048
4.75	-2946.929066	4.85	-2946.887929	4.9	-2946.861354	4.75	-2946.844788	5.0	-2946.834078	4.85	-2946.824747
4.8	-2946.928465	4.9	-2946.887685	5.0	-2946.860827	4.8	-2946.844878	5.2	-2946.832789	4.9	-2946.824438
4.85	-2946.927874	5.0	-2946.887167	5.1	-2946.860247	4.85	-2946.844958	5.3	-2946.832198	5.0	-2946.823804
4.9	-2946.927295	5.1	-2946.886589	5.2	-2946.859671	4.9	-2946.845028	5.4	-2946.831648	5.1	-2946.823213
5.0	-2946.926173	5.2	-2946.886018	5.3	-2946.859056	5.0	-2946.845144	5.5	-2946.831143	5.2	-2946.822490
5.1	-2946.925181	5.3	-2946.885393	5.4	-2946.858426	5.1	-2946.845236	5.6	-2946.830681	5.3	-2946.821824
5.2	-2946.924101	5.4	-2946.884736	5.5	-2946.857791	5.2	-2946.845301	5.7	-2946.830262	5.4	-2946.821161
5.3	-2946.923158	5.5	-2946.884058	5.7	-2946.856506	5.3	-2946.845357	5.8	-2946.829887	5.5	-2946.820519
5.4	-2946.922281	5.6	-2946.883345	5.8	-2946.855873	5.5	-2946.845442	5.9	-2946.829552	5.6	-2946.819888
5.5	-2946.921468	5.7	-2946.882606	5.9	-2946.855252	5.6	-2946.845484	6.0	-2946.829255	5.7	-2946.819289
5.6	-2946.920722	5.8	-2946.881846	6.0	-2946.854648	5.7	-2946.845528	6.2	-2946.828763	5.8	-2946.818739
5.7	-2946.920039	5.9	-2946.881066	6.2	-2946.853503	5.8	-2946.845575	6.4	-2946.828387	5.9	-2946.818258
5.8	-2946.919418	6.0	-2946.880269	6.4	-2946.852463	6.0	-2946.845683	6.6	-2946.828106	6.0	-2946.817871
5.9	-2946.918856	6.2	-2946.878639	6.6	-2946.851545	6.2	-2946.845806	6.8	-2946.827902	6.2	-2946.817448
6.0	-2946.918349	6.4	-2946.876980	6.8	-2946.850757	6.4	-2946.845938	7.0	-2946.827750	6.4	-2946.817468
6.2	-2946.917484	6.6	-2946.875317	7.0	-2946.850097	6.6	-2946.846063	7.4	-2946.827544	6.6	-2946.817762
6.4	-2946.916793	6.8	-2946.873672	7.4	-2946.849111	6.8	-2946.846170	7.6	-2946.827487	6.8	-2946.818186
6.6	-2946.916245	7.0	-2946.872076	7.6	-2946.848727	7.0	-2946.846259	7.8	-2946.827451	7.0	-2946.818648
6.8	-2946.915814	7.4	-2946.869086	7.8	-2946.848371	7.6	-2946.846240	7.95	-2946.827436	7.4	-2946.819468
7.0	-2946.915473	7.6	-2946.867718	7.95	-2946.848106	7.95	-2946.846085	8.0	-2946.827433	7.8	-2946.820205
7.4	-2946.915001	7.8	-2946.866453	8.0	-2946.848015	8.0	-2946.846057	8.05	-2946.827431	7.95	-2946.820458
7.6	-2946.914839	7.95	-2946.865579	8.05	-2946.847924	8.05	-2946.846029	8.2	-2946.827428	8.0	-2946.820540
7.8	-2946.914713	8.0	-2946.865303	8.35	-2946.847355	8.2	-2946.845933	8.35	-2946.827432	8.05	-2946.820620
7.95	-2946.914636	8.05	-2946.865034	8.4	-2946.847260	8.35	-2946.845816	8.4	-2946.827435	8.2	-2946.820851
8.0	-2946.914613	8.2	-2946.864272	8.45	-2946.847165	8.4	-2946.845770	8.45	-2946.827437	8.35	-2946.821069
8.05	-2946.914591	8.35	-2946.863581	8.5	-2946.847073	8.45	-2946.845719	8.5	-2946.827441	8.4	-2946.821139
8.2	-2946.914534	8.4	-2946.863365	8.6	-2946.846899	8.5	-2946.845662	8.6	-2946.827449	8.45	-2946.821208
8.35	-2946.914485	8.45	-2946.863158	8.8	-2946.846615	8.6	-2946.845527	8.8	-2946.827470	8.5	-2946.821275
8.4	-2946.914471	8.5	-2946.862958	9.01	-2946.846424	8.8	-2946.845148	9.01	-2946.827497	8.6	-2946.821406
8.45	-2946.914457	8.6	-2946.862580	9.2	-2946.846320	9.01	-2946.844586	9.2	-2946.827524	9.01	-2946.821888
8.5	-2946.914444	8.8	-2946.861910	9.4	-2946.846251	9.2	-2946.843961	9.4	-2946.827556	9.2	-2946.822084
8.6	-2946.914421	9.01	-2946.861321	9.6	-2946.846208	9.4	-2946.843226	9.8	-2946.827619	9.4	-2946.822276
8.8	-2946.914380	9.2	-2946.860878	9.8	-2946.846181	9.6	-2946.842433	10.2	-2946.827678	9.6	-2946.822450
9.01	-2946.914346	9.4	-2946.860486	10.0	-2946.846164	10.0	-2946.840755	10.4	-2946.827705	9.8	-2946.822608
9.2	-2946.914321	9.6	-2946.860170	10.2	-2946.846154	10.2	-2946.839895	10.6	-2946.827730	10.0	-2946.822753
9.4	-2946.914298	9.8	-2946.859912	10.4	-2946.846148	10.4	-2946.839032	10.8	-2946.827753	10.2	-2946.822884
9.6	-2946.914280	10.0	-2946.859702	10.6	-2946.846145	10.6	-2946.838174	11.2	-2946.827788	10.4	-2946.823004
9.8	-2946.914265	10.2	-2946.859529	10.8	-2946.846145	10.8	-2946.837326	11.4	-2946.827799	10.6	-2946.823112
10.0	-2946.914253	10.4	-2946.859388	11.2	-2946.846148	11.2	-2946.835675	11.6	-2946.827804	10.8	-2946.823210
10.2	-2946.914242	10.6	-2946.859272	11.4	-2946.846151	11.4	-2946.834878	11.8	-2946.827803	11.2	-2946.823377
10.4	-2946.914233	10.8	-2946.859176	11.6	-2946.846155	11.8	-2946.833359	12.0	-2946.827792	11.4	-2946.823447
10.6	-2946.914226	11.2	-2946.859029	12.0	-2946.846162	12.0	-2946.832643	12.2	-2946.827772	11.6	-2946.823508
10.8	-2946.914219	11.4	-2946.858972	12.2	-2946.846165	12.2	-2946.831962	12.4	-2946.827729	11.8	-2946.823561
11.2	-2946.914209	11.6	-2946.858925	12.4	-2946.846168	12.4	-2946.831324	12.6	-2946.827652	12.0	-2946.823605
11.4	-2946.914205	11.8	-2946.858885	12.6	-2946.846170	12.6	-2946.830739	12.8	-2946.827529	12.4	-2946.823667
11.6	-2946.914201	12.0	-2946.858852	12.8	-2946.846173	12.8	-2946.830223	13.0	-2946.827342	12.6	-2946.823686
11.8	-2946.914198	12.2	-2946.858826	12.9	-2946.846174	12.9	-2946.829995	13.1	-2946.827229	12.8	-2946.823694
12.0	-2946.914195	12.4	-2946.858804	13.0	-2946.846175	13.0	-2946.829794	13.2	-2946.827095	12.9	-2946.823693
12.2	-2946.914192	12.6	-2946.858784	13.1	-2946.846177	13.1	-2946.829607	13.3	-2946.826945	13.0	-2946.823695
12.4	-2946.914189	12.8	-2946.858767	13.2	-2946.846178	13.2	-2946.829452	13.4	-2946.826781	13.1	-2946.823679
12.6	-2946.914187	12.9	-2946.858759	13.3	-2946.846179	13.3	-2946.829320	13.8	-2946.826078	13.2	-2946.823668
12.8	-2946.914185	13.0	-2946.858751	13.4	-2946.846180	13.4	-2946.829208	13.9	-2946.825907	13.3	-2946.823652
12.9	-2946.914185	13.1	-2946.858745	13.8	-2946.846185	13.8	-2946.828926	14.0	-2946.825747	13.4	-2946.823627
13.0	-2946.914184	13.2	-2946.858738	13.9	-2946.846186	13.9	-2946.828883	14.1	-2946.825595	13.8	-2946.823461
13.1	-2946.914183	13.3	-2946.858732	14.0	-2946.846186	14.0	-2946.828849	14.2	-2946.825458	14.0	-2946.823328
13.2	-2946.914182	13.4	-2946.858727	14.1	-2946.846187	14.1	-2946.828818	14.3	-2946.825335	14.1	-2946.823219
13.3	-2946.914182	13.8	-2946.858707	14.2	-2946.846188	14.2	-2946.828793	14.4	-2946.825227	14.2	-2946.823110
13.4	-2946.914181	13.9	-2946.858702	14.3	-2946.846189	14.3	-2946.828771	17.0	-2946.824581	14.3	-2946.822984
13.5	-2946.914180	14.0	-2946.858698	14.4	-2946.846190	14.4	-2946.828753	19.0	-2946.824548	14.4	-2946.822845
13.6	-2946.914180	14.1	-2946.858695	17.0	-2946.846204	17.0	-2946.828613	20.0	-2946.824542	17.0	-2946.817770
13.7	-2946.914179	14.2	-2946.858691	19.0	-2946.846208	19.0	-2946.828598	21.0	-2946.824538	19.0	-2946.814317
13.8	-2946.914179	14.3	-2946.858688	20.0	-2946.846209	20.0	-2946.828594	23.0	-2946.824534	20.0	-2946.812869
13.9	-2946.914178	14.4	-2946.858685	21.0	-2946.846210	23.0	-2946.828587	24.0	-2946.824533	21.0	-2946.811564
14.0	-2946.914178	17.0	-2946.858640	23.0	-2946.846211	24.0	-2946.828586	26.0	-2946.824531	23.0	-2946.809318
14.1	-2946.914178	19.0	-2946.858627	24.0	-2946.846211	26.0	-2946.828584	27.0	-2946.824531	24.0	-2946.808575
14.2	-2946.914177	20.0	-2946.858624	27.0	-2946.846212	27.0	-2946.828584	28.0	-2946.824530	26.0	-2946.808536
14.3	-2946.914177	21.0	-2946.858623	28.0	-2946.846212	28.0	-2946.828584	29.0	-2946.824530	27.0	-2946.808535
14.4	-2946.914177	23.0	-2946.858619	29.0	-2946.846212	29.0	-2946.828583	30.0	-2946.824530	28.0	-2946.808534
17.0	-2946.914171	24.0	-2946.858618	30.0	-2946.846212	30.0	-2946.828583	31.0	-2946.824530	29.0	-2946.808532
19.0	-2946.914170	27.0	-2946.858617	31.0	-2946.846212	31.0	-2946.828583	32.0	-2946.824529	30.0	-2946.808532
20.0	-2946.914169	28.0	-2946.858617	32.0	-2946.846212	32.0	-2946.828583	34.0	-2946.824529	31.0	-2946.808531
21.0	-2946.914169	29.0	-2946.858616	33.0	-2946.846212	33.0	-2946.828583	36.0	-2946.824529	32.0	-2946.808531
23.0	-2946.914168	30.0	-2946.858616	34.0	-2946.846212	34.0	-2946.828582	37.0	-2946.824529	33.0	-2946.808531
24.0	-2946.914168	31.0	-2946.858616	36.0	-2946.846212	37.0	-2946.828582	38.0	-2946.824529	34.0	-2946.808530
26.0	-2946.914168	32.0	-2946.858616	37.0	-2946.846212	38.0	-2946.828582	39.0	-2946.824529	36.0	-2946.808530
27.0	-2946.914168	33.0	-2946.858616	38.0	-2946.846212	39.0	-2946.828582	40.0	-2946.824529	37.0	-2946.808529
28.0	-2946.914168	34.0	-2946.858616	39.0	-2946.846212	40.0	-2946.828582	42.0	-2946.824529	38.0	-2946.808529
29.0	-2946.914168	36.0	-2946.858616	40.0	-2946.846212	42.0	-2946.828582	46.0	-2946.824529	39.0	-2946.808529
30.0	-2946.914168	37.0	-2946.858616	42.0	-2946.846212	44.0	-2946.828582	48.0	-2946.824529	40.0	-2946.808529
31.0	-2946.914168	38.0	-2946.858616	44.0	-2946.846212	46.0	-2946.828582	50.0	-2946.824529	42.0	-2946.808528
32.0	-2946.914168	39.0	-2946.858616	46.0	-2946.846212	48.0	-2946.828582	52.0	-2946.824529	44.0	-2946.808528
33.0	-2946.914168	40.0	-2946.858616	48.0	-2946.846212	50.0	-2946.828582	54.0	-2946.824529	46.0	-2946.808528
34.0	-2946.914168	42.0	-2946.858616	50.0	-2946.846212	52.0	-2946.828582	56.0	-2946.824529	48.0	-2946.808528
36.0	-2946.914168	44.0	-2946.858616	52.0	-2946.846212	54.0	-2946.828582	58.0	-2946.824529	50.0	-2946.808528
37.0	-2946.914168	46.0	-2946.858616	54.0	-2946.846212	56.0	-2946.828582	100.0	-2946.824529	52.0	-2946.808528
38.0	-2946.914168	48.0	-2946.858616	56.0	-2946.846212	58.0	-2946.828582	150.0	-2946.824529	54.0	-2946.808528
39.0	-2946.914168	50.0	-2946.858616	58.0	-2946.846212	60.0	-2946.828582	200.0	-2946.824529	56.0	-2946.808528
40.0	-2946.914167	52.0	-2946.858616	60.0	-2946.846212	100.0	-2946.828582			58.0	-2946.808528
42.0	-2946.914167	54.0	-2946.858616	100.0	-2946.846212	150.0	-2946.828582			100.0	-2946.808528
44.0	-2946.914167	56.0	-2946.858616	150.0	-2946.846212	200.0	-2946.828582			150.0	-2946.808528
46.0	-2946.914167	58.0	-2946.858616	200.0	-2946.846212					200.0	-2946.808528
48.0	-2946.914167	60.0	-2946.858616								
50.0	-2946.914167	100.0	-2946.858616								
52.0	-2946.914167	150.0	-2946.858616								
54.0	-2946.914167	200.0	-2946.858616								
56.0	-2946.914167										
58.0	-2946.914167										
60.0	-2946.914167										
100.0	-2946.914167										
150.0	-2946.914167										
200.0	-2946.914167										
