# Supplementary material for: Benchmark Study of the Electronic States of the LiRb Molecule: Ab Initio Calculations with the Fock Space Coupled Cluster Approach
Source: Molecules. 2023 Nov 17;28(22):7645. doi: 10.3390/molecules28227645 (PMC10675596; doi:10.3390/molecules28227645)
Supplement: Supplementary file 1 [file molecules-28-07645-s001.zip › lirb_unanorccplus_sigma_plus_singlet_asymptotic.pdf]

#R[A]	X^1sigma^+
1.4	0.326482
1.5	0.232467
1.6	0.167276
1.7	0.122444
1.8	0.090493
1.9	0.067292
2.0	0.049812
2.1	0.036062
2.2	0.024810
2.3	0.015303
2.4	0.007125
2.5	0.000058
2.6	-0.006014
2.7	-0.011162
2.8	-0.015448
2.9	-0.018933
3.0	-0.021688
3.05	-0.022813
3.1	-0.023783
3.15	-0.024607
3.2	-0.025294
3.25	-0.025853
3.3	-0.026292
3.35	-0.026620
3.45	-0.026975
3.55	-0.027329
3.8	-0.025792
3.85	-0.025397
3.9	-0.024962
3.95	-0.024491
4.0	-0.024000
4.1	-0.022915
4.2	-0.021748
4.3	-0.020529
4.4	-0.019277
4.5	-0.018013
4.6	-0.016779
4.7	-0.015509
4.75	-0.014899
4.8	-0.014298
4.85	-0.013707
4.9	-0.013128
5.0	-0.012006
5.1	-0.011014
5.2	-0.009934
5.3	-0.008991
5.4	-0.008114
5.5	-0.007301
5.6	-0.006555
5.7	-0.005872
5.8	-0.005251
5.9	-0.004689
6.0	-0.004182
6.2	-0.003317
6.4	-0.002626
6.6	-0.002078
6.8	-0.001647
7.0	-0.001306
7.4	-0.000834
7.6	-0.000672
7.8	-0.000546
7.95	-0.000469
8.0	-0.000446
8.05	-0.000424
8.2	-0.000367
8.35	-0.000318
8.4	-0.000304
8.45	-0.000290
8.5	-0.000277
8.6	-0.000254
8.8	-0.000213
9.01	-0.000179
9.2	-0.000154
9.4	-0.000131
9.6	-0.000113
9.8	-0.000098
10.0	-0.000086
10.2	-0.000075
10.4	-0.000066
10.6	-0.000059
10.8	-0.000052
11.2	-0.000042
11.4	-0.000038
11.6	-0.000034
11.8	-0.000031
12.0	-0.000028
12.2	-0.000025
12.4	-0.000022
12.6	-0.000020
12.8	-0.000018
12.9	-0.000018
13.0	-0.000017
13.1	-0.000016
13.2	-0.000015
13.3	-0.000015
13.4	-0.000014
13.5	-0.000013
13.6	-0.000013
13.7	-0.000012
13.8	-0.000012
13.9	-0.000011
14.0	-0.000011
14.1	-0.000011
14.2	-0.000010
14.3	-0.000010
14.4	-0.000010
17.0	-0.000004
19.0	-0.000003
20.0	-0.000002
21.0	-0.000002
23.0	-0.000001
24.0	-0.000001
26.0	-0.000001
27.0	-0.000001
28.0	-0.000001
29.0	-0.000001
30.0	-0.000001
31.0	-0.000001
32.0	-0.000001
33.0	-0.000001
34.0	-0.000001
36.0	-0.000001
37.0	-0.000001
38.0	-0.000001
39.0	-0.000001
40.0	0.000000
42.0	0.000000
44.0	0.000000
46.0	0.000000
48.0	0.000000
50.0	0.000000
52.0	0.000000
54.0	0.000000
56.0	0.000000
58.0	0.000000
60.0	0.000000
100.0	0.000000
150.0	0.000000
200.0	0.000000

R[A]	2^1sigma^+
1.5	0.306728
1.6	0.240599
1.7	0.194934
1.8	0.163033
1.9	0.139971
2.0	0.122974
2.1	0.109650
2.2	0.098563
2.3	0.088889
2.4	0.080129
2.5	0.072100
2.6	0.064771
2.7	0.058159
2.8	0.052279
2.9	0.047126
3.0	0.042669
3.05	0.040688
3.1	0.038864
3.15	0.037188
3.2	0.035654
3.25	0.034254
3.3	0.032981
3.45	0.029846
3.55	0.028501
3.8	0.025683
3.85	0.025365
3.9	0.025100
3.95	0.024886
4.0	0.024692
4.1	0.024484
4.2	0.024419
4.3	0.024474
4.4	0.024631
4.5	0.024873
4.6	0.025170
4.7	0.025577
4.75	0.025785
4.8	0.026006
4.85	0.026238
4.9	0.026482
5.0	0.027000
5.1	0.027578
5.2	0.028149
5.3	0.028774
5.4	0.029431
5.5	0.030109
5.6	0.030822
5.7	0.031561
5.8	0.032321
5.9	0.033101
6.0	0.033898
6.2	0.035528
6.4	0.037187
6.6	0.038850
6.8	0.040495
7.0	0.042091
7.4	0.045081
7.6	0.046449
7.8	0.047714
7.95	0.048588
8.0	0.048864
8.05	0.049133
8.2	0.049895
8.35	0.050586
8.4	0.050802
8.45	0.051009
8.5	0.051209
8.6	0.051587
8.8	0.052257
9.01	0.052846
9.2	0.053289
9.4	0.053681
9.6	0.053997
9.8	0.054255
10.0	0.054465
10.2	0.054638
10.4	0.054779
10.6	0.054895
10.8	0.054991
11.2	0.055138
11.4	0.055195
11.6	0.055242
11.8	0.055282
12.0	0.055315
12.2	0.055341
12.4	0.055363
12.6	0.055383
12.8	0.055400
12.9	0.055408
13.0	0.055416
13.1	0.055422
13.2	0.055429
13.3	0.055435
13.4	0.055440
13.8	0.055460
13.9	0.055465
14.0	0.055469
14.1	0.055472
14.2	0.055476
14.3	0.055479
14.4	0.055482
17.0	0.055527
19.0	0.055540
20.0	0.055543
21.0	0.055544
23.0	0.055548
24.0	0.055549
27.0	0.055550
28.0	0.055550
29.0	0.055551
30.0	0.055551
31.0	0.055551
32.0	0.055551
33.0	0.055551
34.0	0.055551
36.0	0.055551
37.0	0.055551
38.0	0.055551
39.0	0.055551
40.0	0.055551
42.0	0.055551
44.0	0.055551
46.0	0.055551
48.0	0.055551
50.0	0.055551
52.0	0.055551
54.0	0.055551
56.0	0.055551
58.0	0.055551
60.0	0.055551
100.0	0.055551
150.0	0.055551
200.0	0.055551

R[A]	3^1sigma^+
1.4	0.419988
1.5	0.321182
1.6	0.252667
1.7	0.205322
1.8	0.172290
2.0	0.132514
2.1	0.120431
2.2	0.111349
2.3	0.104302
2.4	0.098625
2.5	0.093901
2.6	0.089870
2.7	0.083677
2.8	0.078171
2.9	0.073341
3.0	0.069141
3.05	0.067262
3.1	0.065523
3.15	0.063917
3.2	0.062438
3.25	0.061080
3.3	0.059837
3.35	0.058703
3.45	0.056736
3.55	0.055360
3.8	0.052435
3.85	0.052084
3.9	0.051785
3.95	0.051537
4.0	0.051313
4.1	0.051034
4.2	0.050906
4.3	0.050906
4.6	0.051692
4.7	0.051903
4.75	0.052110
4.8	0.052331
4.85	0.052566
4.9	0.052813
5.0	0.053340
5.1	0.053920
5.2	0.054496
5.3	0.055111
5.4	0.055741
5.5	0.056376
5.7	0.057661
5.8	0.058294
5.9	0.058915
6.0	0.059519
6.2	0.060664
6.4	0.061704
6.6	0.062622
6.8	0.063410
7.0	0.064070
7.4	0.065056
7.6	0.065440
7.8	0.065796
7.95	0.066061
8.0	0.066152
8.05	0.066243
8.35	0.066812
8.4	0.066907
8.45	0.067002
8.5	0.067094
8.6	0.067268
8.8	0.067552
9.01	0.067743
9.2	0.067847
9.4	0.067916
9.6	0.067959
9.8	0.067986
10.0	0.068003
10.2	0.068013
10.4	0.068019
10.6	0.068022
10.8	0.068022
11.2	0.068019
11.4	0.068016
11.6	0.068012
12.0	0.068005
12.2	0.068002
12.4	0.067999
12.6	0.067997
12.8	0.067994
12.9	0.067993
13.0	0.067992
13.1	0.067990
13.2	0.067989
13.3	0.067988
13.4	0.067987
13.8	0.067982
13.9	0.067981
14.0	0.067981
14.1	0.067980
14.2	0.067979
14.3	0.067978
14.4	0.067977
17.0	0.067963
19.0	0.067959
20.0	0.067958
21.0	0.067957
23.0	0.067956
24.0	0.067956
27.0	0.067955
28.0	0.067955
29.0	0.067955
30.0	0.067955
31.0	0.067955
32.0	0.067955
33.0	0.067955
34.0	0.067955
36.0	0.067955
37.0	0.067955
38.0	0.067955
39.0	0.067955
40.0	0.067955
42.0	0.067955
44.0	0.067955
46.0	0.067955
48.0	0.067955
50.0	0.067955
52.0	0.067955
54.0	0.067955
56.0	0.067955
58.0	0.067955
60.0	0.067955
100.0	0.067955
150.0	0.067955
200.0	0.067955

R[A]	4^1sigma^+
1.4	0.456130
1.5	0.358942
1.6	0.289577
1.7	0.241367
1.8	0.207598
1.9	0.183260
2.0	0.164960
2.1	0.150522
2.2	0.138561
2.3	0.128341
2.4	0.119493
2.5	0.111790
2.6	0.105068
2.7	0.099202
2.8	0.094108
2.9	0.089723
3.0	0.085995
3.05	0.084363
3.1	0.082875
3.15	0.081526
3.2	0.080309
3.25	0.079218
3.3	0.078245
3.35	0.077384
3.45	0.075968
3.55	0.074968
3.8	0.073419
3.85	0.073217
3.9	0.073002
3.95	0.072759
4.0	0.072450
4.1	0.071875
4.2	0.071316
4.3	0.070815
4.4	0.070385
4.5	0.070024
4.6	0.069707
4.7	0.069480
4.75	0.069379
4.8	0.069289
4.85	0.069209
4.9	0.069139
5.0	0.069023
5.1	0.068931
5.2	0.068866
5.3	0.068810
5.5	0.068725
5.6	0.068683
5.7	0.068639
5.8	0.068592
6.0	0.068484
6.2	0.068361
6.4	0.068229
6.6	0.068104
6.8	0.067997
7.0	0.067908
7.6	0.067927
7.95	0.068082
8.0	0.068110
8.05	0.068138
8.2	0.068234
8.35	0.068351
8.4	0.068397
8.45	0.068448
8.5	0.068505
8.6	0.068640
8.8	0.069019
9.01	0.069581
9.2	0.070206
9.4	0.070941
9.6	0.071734
10.0	0.073412
10.2	0.074272
10.4	0.075135
10.6	0.075993
10.8	0.076841
11.2	0.078492
11.4	0.079289
11.8	0.080808
12.0	0.081524
12.2	0.082205
12.4	0.082843
12.6	0.083428
12.8	0.083944
12.9	0.084172
13.0	0.084373
13.1	0.084560
13.2	0.084715
13.3	0.084847
13.4	0.084959
13.8	0.085241
13.9	0.085284
14.0	0.085318
14.1	0.085349
14.2	0.085374
14.3	0.085396
14.4	0.085414
17.0	0.085554
19.0	0.085569
20.0	0.085573
23.0	0.085580
24.0	0.085581
26.0	0.085583
27.0	0.085583
28.0	0.085583
29.0	0.085584
30.0	0.085584
31.0	0.085584
32.0	0.085584
33.0	0.085584
34.0	0.085585
37.0	0.085585
38.0	0.085585
39.0	0.085585
40.0	0.085585
42.0	0.085585
44.0	0.085585
46.0	0.085585
48.0	0.085585
50.0	0.085585
52.0	0.085585
54.0	0.085585
56.0	0.085585
58.0	0.085585
60.0	0.085585
100.0	0.085585
150.0	0.085585
200.0	0.085585

R[A]	5^1sigma^+
1.4	0.459256
1.5	0.359553
1.6	0.292081
1.7	0.246131
1.8	0.214228
2.0	0.174018
2.1	0.159807
2.2	0.148150
2.3	0.138163
2.4	0.129348
2.5	0.121472
2.6	0.114441
2.7	0.108202
2.8	0.102706
2.9	0.097892
3.0	0.093696
3.05	0.091809
3.1	0.090053
3.15	0.088419
3.2	0.086901
3.25	0.085491
3.3	0.084183
3.35	0.082972
3.45	0.080814
3.8	0.075658
3.85	0.075237
3.95	0.074722
4.0	0.074632
4.1	0.074705
4.2	0.075009
4.3	0.075462
4.4	0.076014
4.5	0.076636
4.6	0.077337
4.7	0.078001
4.75	0.078352
4.8	0.078704
4.9	0.079404
5.0	0.080089
5.2	0.081378
5.3	0.081969
5.4	0.082519
5.5	0.083024
5.6	0.083486
5.7	0.083905
5.8	0.084280
5.9	0.084615
6.0	0.084912
6.2	0.085404
6.4	0.085780
6.6	0.086061
6.8	0.086265
7.0	0.086417
7.4	0.086623
7.6	0.086680
7.8	0.086716
7.95	0.086731
8.0	0.086734
8.05	0.086736
8.2	0.086739
8.35	0.086735
8.4	0.086732
8.45	0.086730
8.5	0.086726
8.6	0.086718
8.8	0.086697
9.01	0.086670
9.2	0.086643
9.4	0.086611
9.8	0.086548
10.2	0.086489
10.4	0.086462
10.6	0.086437
10.8	0.086414
11.2	0.086379
11.4	0.086368
11.6	0.086363
11.8	0.086364
12.0	0.086375
12.2	0.086395
12.4	0.086438
12.6	0.086515
12.8	0.086638
13.0	0.086825
13.1	0.086938
13.2	0.087072
13.3	0.087222
13.4	0.087386
13.8	0.088089
13.9	0.088260
14.0	0.088420
14.1	0.088572
14.2	0.088709
14.3	0.088832
14.4	0.088940
17.0	0.089586
19.0	0.089619
20.0	0.089625
21.0	0.089629
23.0	0.089633
24.0	0.089634
26.0	0.089636
27.0	0.089636
28.0	0.089637
29.0	0.089637
30.0	0.089637
31.0	0.089637
32.0	0.089638
34.0	0.089638
36.0	0.089638
37.0	0.089638
38.0	0.089638
39.0	0.089638
40.0	0.089638
42.0	0.089638
46.0	0.089638
48.0	0.089638
50.0	0.089638
52.0	0.089638
54.0	0.089638
56.0	0.089638
58.0	0.089638
100.0	0.089638
150.0	0.089638
200.0	0.089638

R[A]	6^1sigma^+
1.4	0.460147
1.5	0.362952
1.6	0.295944
1.7	0.249444
1.8	0.216262
1.9	0.192161
2.1	0.163259
2.2	0.153517
2.3	0.145355
2.4	0.138203
2.5	0.131779
2.6	0.124628
2.7	0.118023
2.8	0.112218
3.0	0.102860
3.05	0.100953
3.1	0.099205
3.15	0.097607
3.2	0.096152
3.25	0.094832
3.3	0.093638
3.35	0.092564
3.45	0.090745
3.55	0.089325
3.8	0.087146
3.85	0.086908
3.9	0.086726
3.95	0.086594
4.0	0.086510
4.1	0.086467
4.2	0.086570
4.3	0.086795
4.4	0.087122
4.5	0.087532
4.6	0.088004
4.7	0.088542
4.75	0.088826
4.8	0.089119
4.85	0.089420
4.9	0.089729
5.0	0.090363
5.1	0.090954
5.2	0.091677
5.3	0.092343
5.4	0.093006
5.5	0.093648
5.6	0.094279
5.7	0.094878
5.8	0.095428
5.9	0.095909
6.0	0.096296
6.2	0.096719
6.4	0.096699
6.6	0.096405
6.8	0.095981
7.0	0.095519
7.4	0.094699
7.8	0.093962
7.95	0.093709
8.0	0.093627
8.05	0.093547
8.2	0.093316
8.35	0.093098
8.4	0.093028
8.45	0.092959
8.5	0.092892
8.6	0.092761
9.01	0.092279
9.2	0.092083
9.4	0.091891
9.6	0.091717
9.8	0.091559
10.0	0.091414
10.2	0.091283
10.4	0.091163
10.6	0.091055
10.8	0.090957
11.2	0.090790
11.4	0.090720
11.6	0.090659
11.8	0.090606
12.0	0.090562
12.4	0.090500
12.6	0.090481
12.8	0.090473
12.9	0.090474
13.0	0.090472
13.1	0.090488
13.2	0.090499
13.3	0.090515
13.4	0.090540
13.8	0.090706
14.0	0.090839
14.1	0.090948
14.2	0.091057
14.3	0.091183
14.4	0.091322
17.0	0.096397
19.0	0.099850
20.0	0.101298
21.0	0.102603
23.0	0.104849
24.0	0.105592
26.0	0.105631
27.0	0.105632
28.0	0.105633
29.0	0.105635
30.0	0.105635
31.0	0.105636
32.0	0.105636
33.0	0.105636
34.0	0.105637
36.0	0.105637
37.0	0.105638
38.0	0.105638
39.0	0.105638
40.0	0.105638
42.0	0.105639
44.0	0.105639
46.0	0.105639
48.0	0.105639
50.0	0.105639
52.0	0.105639
54.0	0.105639
56.0	0.105639
58.0	0.105639
100.0	0.105639
150.0	0.105639
200.0	0.105639
