# Supplementary material for: Benchmark Study of the Electronic States of the LiRb Molecule: Ab Initio Calculations with the Fock Space Coupled Cluster Approach
Source: Molecules. 2023 Nov 17;28(22):7645. doi: 10.3390/molecules28227645 (PMC10675596; doi:10.3390/molecules28227645)
Supplement: Supplementary file 1 [file molecules-28-07645-s001.zip › lirb_unanorccplus_sigma_plus_triplet.pdf]

#R[A]	1^3 sigma^+	R[A]	2^3 sigma^+	R[A]	3^3 sigma^+	R[A]	4^3 sigma^+	R[A]	5^3 sigma^+	R[A]	6^3 sigma^+
1.4	-2946.534107	1.4	-2946.483739	1.4	-2946.472361	1.4	-2946.451251	1.4	-2946.445112	1.4	-2946.442365
1.5	-2946.631158	1.5	-2946.579017	1.5	-2946.571166	1.5	-2946.549890	1.6	-2946.610263	1.5	-2946.539084
1.6	-2946.698680	1.6	-2946.644703	1.7	-2946.687582	1.6	-2946.617669	1.7	-2946.656884	1.6	-2946.605850
1.7	-2946.745579	1.8	-2946.721376	1.8	-2946.721255	1.7	-2946.664122	1.8	-2946.689247	1.7	-2946.652370
1.8	-2946.778916	1.9	-2946.745577	1.9	-2946.743804	1.8	-2946.695960	1.9	-2946.711991	1.8	-2946.685384
1.9	-2946.803009	2.0	-2946.763517	2.0	-2946.760224	2.0	-2946.733009	2.0	-2946.728396	1.9	-2946.709489
2.0	-2946.820962	2.1	-2946.777300	2.1	-2946.772741	2.1	-2946.743617	2.1	-2946.742680	2.0	-2946.727677
2.1	-2946.834827	2.2	-2946.788354	2.2	-2946.782744	2.3	-2946.766336	2.2	-2946.752316	2.1	-2946.740546
2.2	-2946.845990	2.3	-2946.797610	2.3	-2946.795581	2.4	-2946.776444	2.3	-2946.763569	2.2	-2946.751123
2.3	-2946.855294	2.4	-2946.807954	2.4	-2946.805587	2.5	-2946.785506	2.5	-2946.782184	2.3	-2946.756701
2.4	-2946.863301	2.5	-2946.819046	2.5	-2946.812661	2.6	-2946.793542	2.6	-2946.789903	2.4	-2946.763909
2.5	-2946.870335	2.6	-2946.828853	2.6	-2946.818954	2.7	-2946.800608	2.7	-2946.796700	2.5	-2946.772390
2.6	-2946.876578	2.7	-2946.837392	2.7	-2946.824544	2.8	-2946.806764	2.8	-2946.802621	2.6	-2946.780066
2.7	-2946.882135	2.8	-2946.844731	2.8	-2946.829466	2.9	-2946.812078	2.9	-2946.807718	2.7	-2946.786842
2.8	-2946.887067	2.9	-2946.850970	2.9	-2946.833750	3.0	-2946.816620	3.0	-2946.812051	2.8	-2946.792763
2.9	-2946.891423	3.0	-2946.856223	3.0	-2946.837425	3.05	-2946.818625	3.05	-2946.813951	2.9	-2946.797883
3.0	-2946.895244	3.05	-2946.858515	3.05	-2946.839046	3.1	-2946.820464	3.1	-2946.815686	3.0	-2946.802267
3.05	-2946.896968	3.1	-2946.860603	3.1	-2946.840530	3.15	-2946.822148	3.15	-2946.817264	3.05	-2946.804204
3.1	-2946.898573	3.15	-2946.862501	3.15	-2946.841882	3.2	-2946.823683	3.2	-2946.818695	3.1	-2946.805981
3.15	-2946.900067	3.2	-2946.864221	3.2	-2946.843108	3.25	-2946.825081	3.25	-2946.819987	3.15	-2946.807608
3.2	-2946.901453	3.25	-2946.865775	3.25	-2946.844213	3.3	-2946.826348	3.3	-2946.821148	3.2	-2946.809092
3.25	-2946.902738	3.3	-2946.867176	3.3	-2946.845204	3.35	-2946.827493	3.35	-2946.822189	3.3	-2946.811664
3.3	-2946.903927	3.35	-2946.868434	3.35	-2946.846087	3.45	-2946.829448	3.45	-2946.823939	3.35	-2946.812767
3.35	-2946.905026	3.45	-2946.870561	3.45	-2946.847553	3.55	-2946.830943	3.55	-2946.825288	3.45	-2946.814646
3.45	-2946.906972	3.55	-2946.872224	3.55	-2946.848623	3.8	-2946.833498	3.8	-2946.827405	3.55	-2946.816132
3.55	-2946.908569	3.85	-2946.875123	3.8	-2946.850156	3.85	-2946.833801	3.85	-2946.827662	3.8	-2946.818457
3.8	-2946.911651	3.9	-2946.875362	3.85	-2946.850278	3.9	-2946.834050	3.9	-2946.827884	3.85	-2946.818878
3.85	-2946.912106	3.95	-2946.875545	3.9	-2946.850352	3.95	-2946.834249	3.95	-2946.828079	3.9	-2946.819710
3.9	-2946.912517	4.0	-2946.875743	3.95	-2946.850382	4.0	-2946.834412	4.1	-2946.828618	3.95	-2946.820511
3.95	-2946.912890	4.1	-2946.875869	4.0	-2946.850365	4.1	-2946.834595	4.2	-2946.829049	4.0	-2946.821324
4.0	-2946.913224	4.2	-2946.875840	4.1	-2946.850236	4.2	-2946.834638	4.3	-2946.829631	4.1	-2946.822581
4.1	-2946.913798	4.3	-2946.875684	4.2	-2946.849989	4.3	-2946.834564	4.4	-2946.830360	4.2	-2946.823487
4.2	-2946.914259	4.4	-2946.875423	4.3	-2946.849648	4.4	-2946.834392	4.5	-2946.831174	4.3	-2946.824005
4.3	-2946.914624	4.5	-2946.875077	4.4	-2946.849235	4.5	-2946.834142	4.6	-2946.831944	4.4	-2946.824168
4.4	-2946.914909	4.6	-2946.874608	4.5	-2946.848771	4.6	-2946.833848	4.75	-2946.832800	4.6	-2946.823805
4.5	-2946.915128	4.7	-2946.874155	4.6	-2946.848295	4.7	-2946.833463	4.8	-2946.833182	4.7	-2946.823349
4.6	-2946.915281	4.75	-2946.873905	4.7	-2946.847772	4.75	-2946.833272	4.85	-2946.833060	4.75	-2946.823115
4.7	-2946.915410	4.85	-2946.873380	4.75	-2946.847513	4.8	-2946.833563	4.8	-2946.832850	4.85	-2946.822601
4.75	-2946.915453	4.9	-2946.873107	4.8	-2946.847254	4.85	-2946.833919	4.9	-2946.832633	4.9	-2946.822327
4.8	-2946.915489	5.0	-2946.872546	4.85	-2946.846998	4.9	-2946.834258	5.0	-2946.832188	5.0	-2946.821756
4.85	-2946.915516	5.1	-2946.872256	4.9	-2946.846747	5.0	-2946.834880	5.1	-2946.831830	5.1	-2946.821147
4.9	-2946.915537	5.2	-2946.871389	5.0	-2946.846263	5.1	-2946.835448	5.2	-2946.831277	5.2	-2946.820562
5.0	-2946.915561	5.3	-2946.870805	5.1	-2946.845873	5.2	-2946.835873	5.4	-2946.830384	5.3	-2946.819957
5.1	-2946.915516	5.4	-2946.870226	5.3	-2946.845070	5.3	-2946.836235	5.6	-2946.829548	5.4	-2946.819353
5.2	-2946.915554	5.5	-2946.869687	5.4	-2946.844791	5.4	-2946.836502	5.7	-2946.829160	5.5	-2946.818759
5.3	-2946.915531	5.6	-2946.869125	5.5	-2946.844577	5.5	-2946.836689	5.9	-2946.828456	5.6	-2946.818172
5.4	-2946.915498	5.7	-2946.868577	5.6	-2946.844439	5.6	-2946.836770	6.0	-2946.828141	5.7	-2946.817596
5.5	-2946.915455	5.8	-2946.868045	5.7	-2946.844365	5.8	-2946.836697	6.2	-2946.827588	5.8	-2946.817035
5.6	-2946.915410	5.9	-2946.867531	5.8	-2946.844348	5.9	-2946.836567	6.4	-2946.827133	5.9	-2946.816491
5.7	-2946.915361	6.0	-2946.867036	5.9	-2946.844375	6.2	-2946.835951	6.6	-2946.826765	6.0	-2946.815964
5.8	-2946.915311	6.2	-2946.866103	6.0	-2946.844436	6.4	-2946.835447	6.8	-2946.826473	6.2	-2946.814965
5.9	-2946.915258	6.4	-2946.865252	6.2	-2946.844619	6.6	-2946.834924	7.0	-2946.826242	6.4	-2946.814044
6.0	-2946.915206	6.6	-2946.864482	6.4	-2946.844833	6.8	-2946.834398	7.4	-2946.825927	6.8	-2946.812451
6.2	-2946.915101	6.8	-2946.863796	6.6	-2946.845043	7.0	-2946.833870	7.6	-2946.825821	7.0	-2946.811784
6.4	-2946.915000	7.0	-2946.863188	6.8	-2946.845228	7.4	-2946.832940	7.8	-2946.825737	7.4	-2946.810737
6.6	-2946.914905	7.4	-2946.862152	7.0	-2946.845384	7.6	-2946.832527	7.95	-2946.825686	7.6	-2946.810425
6.8	-2946.914818	7.6	-2946.861723	7.4	-2946.845635	7.95	-2946.831875	8.0	-2946.825670	7.8	-2946.810274
7.0	-2946.914739	7.8	-2946.861347	7.6	-2946.845727	8.0	-2946.831788	8.05	-2946.825656	7.95	-2946.810239
7.4	-2946.914606	7.95	-2946.861095	7.8	-2946.845801	8.2	-2946.831460	8.2	-2946.825615	8.0	-2946.810238
7.6	-2946.914551	8.0	-2946.861016	7.95	-2946.845847	8.35	-2946.831231	8.35	-2946.825579	8.05	-2946.810240
7.8	-2946.914503	8.05	-2946.860940	8.0	-2946.845861	8.4	-2946.831158	8.4	-2946.825568	8.2	-2946.810263
7.95	-2946.914471	8.35	-2946.860533	8.05	-2946.845874	8.45	-2946.831086	8.45	-2946.825557	8.35	-2946.810300
8.0	-2946.914461	8.4	-2946.860473	8.2	-2946.845909	8.5	-2946.831016	8.5	-2946.825546	8.4	-2946.810313
8.05	-2946.914451	8.45	-2946.860415	8.35	-2946.845938	8.6	-2946.830881	8.6	-2946.825525	8.45	-2946.810327
8.2	-2946.914424	8.5	-2946.860359	8.4	-2946.845947	8.8	-2946.830627	8.8	-2946.825486	8.5	-2946.810342
8.35	-2946.914399	8.6	-2946.860251	8.45	-2946.845956	9.01	-2946.830385	9.01	-2946.825445	8.8	-2946.810421
8.4	-2946.914392	8.8	-2946.860057	8.5	-2946.845964	9.2	-2946.830185	9.2	-2946.825409	9.01	-2946.810464
8.45	-2946.914384	9.01	-2946.859879	8.6	-2946.845979	9.4	-2946.829996	9.4	-2946.825370	9.2	-2946.810491
8.5	-2946.914377	9.2	-2946.859738	8.8	-2946.846005	9.6	-2946.829824	9.6	-2946.825331	9.4	-2946.810479
8.6	-2946.914364	9.4	-2946.859612	9.01	-2946.846028	9.8	-2946.829669	9.8	-2946.825290	9.6	-2946.810484
8.8	-2946.914339	9.6	-2946.859497	9.2	-2946.846045	10.0	-2946.829530	10.0	-2946.825249	9.8	-2946.810477
9.01	-2946.914317	9.8	-2946.859396	9.4	-2946.846059	10.2	-2946.829406	10.2	-2946.825208	10.0	-2946.810458
9.2	-2946.914300	10.0	-2946.859307	9.6	-2946.846071	10.4	-2946.829296	10.4	-2946.825166	10.2	-2946.810429
9.4	-2946.914283	10.2	-2946.859228	9.8	-2946.846082	10.6	-2946.829198	10.6	-2946.825125	10.4	-2946.810389
9.6	-2946.914269	10.4	-2946.859159	10.0	-2946.846092	10.8	-2946.829112	11.2	-2946.825008	10.6	-2946.810340
9.8	-2946.914257	10.8	-2946.859044	10.2	-2946.846101	11.2	-2946.828972	11.4	-2946.824971	10.8	-2946.810284
10.0	-2946.914247	11.2	-2946.858954	10.6	-2946.846117	11.4	-2946.828915	11.6	-2946.824937	11.4	-2946.810081
10.2	-2946.914238	11.4	-2946.858917	10.8	-2946.846124	11.8	-2946.828827	11.8	-2946.824904	11.6	-2946.810005
10.4	-2946.914230	11.6	-2946.858884	11.2	-2946.846137	12.0	-2946.828797	12.0	-2946.824873	11.8	-2946.809930
10.6	-2946.914223	11.8	-2946.858854	11.6	-2946.846149	12.2	-2946.828779	12.2	-2946.824843	12.0	-2946.809862
10.8	-2946.914218	12.0	-2946.858828	11.8	-2946.846154	12.4	-2946.828764	12.4	-2946.824815	12.2	-2946.809808
11.2	-2946.914208	12.2	-2946.858807	12.0	-2946.846159	12.6	-2946.828750	12.6	-2946.824790	12.4	-2946.809740
11.4	-2946.914204	12.4	-2946.858788	12.2	-2946.846162	12.8	-2946.828736	12.8	-2946.824767	12.6	-2946.809666
11.6	-2946.914200	12.6	-2946.858772	12.4	-2946.846166	12.9	-2946.828730	12.9	-2946.824757	12.8	-2946.809593
11.8	-2946.914197	12.8	-2946.858757	12.6	-2946.846169	13.0	-2946.828725	13.1	-2946.824736	12.9	-2946.809557
12.0	-2946.914194	12.9	-2946.858751	12.8	-2946.846172	13.1	-2946.828718	13.2	-2946.824727	13.0	-2946.809523
12.2	-2946.914192	13.0	-2946.858744	12.9	-2946.846174	13.2	-2946.828713	13.3	-2946.824718	13.1	-2946.809487
12.4	-2946.914189	13.2	-2946.858733	13.0	-2946.846175	13.3	-2946.828708	13.4	-2946.824709	13.2	-2946.809453
12.6	-2946.914187	13.3	-2946.858727	13.1	-2946.846176	13.4	-2946.828703	13.8	-2946.824679	13.3	-2946.809420
12.8	-2946.914185	13.4	-2946.858723	13.2	-2946.846178	13.8	-2946.828686	13.9	-2946.824672	13.4	-2946.809389
12.9	-2946.914185	13.8	-2946.858704	13.3	-2946.846179	13.9	-2946.828682	14.0	-2946.824666	13.8	-2946.809265
13.0	-2946.914184	13.9	-2946.858700	13.4	-2946.846180	14.0	-2946.828678	14.1	-2946.824659	13.9	-2946.809236
13.1	-2946.914183	14.0	-2946.858696	13.8	-2946.846184	14.1	-2946.828675	14.2	-2946.824653	14.3	-2946.809129
13.2	-2946.914182	14.1	-2946.858693	13.9	-2946.846185	14.2	-2946.828672	14.3	-2946.824648	14.4	-2946.809104
13.3	-2946.914182	14.2	-2946.858690	14.0	-2946.846186	14.3	-2946.828668	14.4	-2946.824642	17.0	-2946.808702
13.4	-2946.914181	14.3	-2946.858686	14.1	-2946.846187	14.4	-2946.828665	17.0	-2946.824567	19.0	-2946.808601
13.5	-2946.914180	14.4	-2946.858683	14.2	-2946.846188	17.0	-2946.828611	19.0	-2946.824547	20.0	-2946.808576
13.6	-2946.914180	19.0	-2946.858627	14.3	-2946.846189	19.0	-2946.828598	20.0	-2946.824542	21.0	-2946.808559
13.7	-2946.914179	21.0	-2946.858623	14.4	-2946.846190	20.0	-2946.828594	21.0	-2946.824538	23.0	-2946.808543
13.8	-2946.914179	23.0	-2946.858619	17.0	-2946.846204	21.0	-2946.828591	23.0	-2946.824534	24.0	-2946.808539
13.9	-2946.914178	24.0	-2946.858618	19.0	-2946.846208	23.0	-2946.828587	26.0	-2946.824531	26.0	-2946.808535
14.0	-2946.914178	27.0	-2946.858617	20.0	-2946.846209	24.0	-2946.828586	27.0	-2946.824531	27.0	-2946.808535
14.1	-2946.914178	28.0	-2946.858617	21.0	-2946.846210	26.0	-2946.828584	28.0	-2946.824530	28.0	-2946.808533
14.2	-2946.914177	29.0	-2946.858616	23.0	-2946.846211	27.0	-2946.828584	29.0	-2946.824530	29.0	-2946.808532
14.3	-2946.914177	31.0	-2946.858616	24.0	-2946.846211	28.0	-2946.828584	30.0	-2946.824530	30.0	-2946.808532
14.4	-2946.914177	32.0	-2946.858616	27.0	-2946.846212	29.0	-2946.828583	31.0	-2946.824530	31.0	-2946.808531
17.0	-2946.914171	33.0	-2946.858616	28.0	-2946.846212	30.0	-2946.828583	32.0	-2946.824529	32.0	-2946.808531
19.0	-2946.914170	34.0	-2946.858616	29.0	-2946.846212	31.0	-2946.828583	33.0	-2946.824529	33.0	-2946.808531
20.0	-2946.914169	36.0	-2946.858616	30.0	-2946.846212	32.0	-2946.828583	34.0	-2946.824529	34.0	-2946.808530
21.0	-2946.914169	37.0	-2946.858616	31.0	-2946.846212	33.0	-2946.828582	36.0	-2946.824529	36.0	-2946.808530
23.0	-2946.914168	38.0	-2946.858616	32.0	-2946.846212	34.0	-2946.828582	37.0	-2946.824529	37.0	-2946.808529
24.0	-2946.914168	39.0	-2946.858616	33.0	-2946.846212	37.0	-2946.828582	38.0	-2946.824529	38.0	-2946.808529
26.0	-2946.914168	40.0	-2946.858616	34.0	-2946.846212	38.0	-2946.828582	39.0	-2946.824529	39.0	-2946.808529
27.0	-2946.914168	42.0	-2946.858616	36.0	-2946.846212	39.0	-2946.828582	40.0	-2946.824529	40.0	-2946.808529
28.0	-2946.914168	44.0	-2946.858616	37.0	-2946.846212	40.0	-2946.828582	42.0	-2946.824529	42.0	-2946.808528
29.0	-2946.914168	46.0	-2946.858616	38.0	-2946.846212	42.0	-2946.828582	44.0	-2946.824529	44.0	-2946.808528
30.0	-2946.914168	48.0	-2946.858616	39.0	-2946.846212	44.0	-2946.828582	46.0	-2946.824529	46.0	-2946.808528
31.0	-2946.914168	50.0	-2946.858616	40.0	-2946.846212	46.0	-2946.828582	48.0	-2946.824529	48.0	-2946.808528
32.0	-2946.914168	52.0	-2946.858616	42.0	-2946.846212	48.0	-2946.828582	50.0	-2946.824529	50.0	-2946.808528
33.0	-2946.914168	54.0	-2946.858616	44.0	-2946.846212	50.0	-2946.828582	52.0	-2946.824529	52.0	-2946.808528
34.0	-2946.914168	56.0	-2946.858616	46.0	-2946.846212	52.0	-2946.828582	54.0	-2946.824529	54.0	-2946.808528
36.0	-2946.914168	58.0	-2946.858616	48.0	-2946.846212	54.0	-2946.828582	56.0	-2946.824529	56.0	-2946.808528
37.0	-2946.914168	60.0	-2946.858616	50.0	-2946.846212	56.0	-2946.828582	58.0	-2946.824529	58.0	-2946.808528
38.0	-2946.914168	100.0	-2946.858616	52.0	-2946.846212	58.0	-2946.828582	100.0	-2946.824529	100.0	-2946.808528
39.0	-2946.914168	150.0	-2946.858616	54.0	-2946.846212	60.0	-2946.828582	150.0	-2946.824529	150.0	-2946.808528
40.0	-2946.914167	200.0	-2946.858616	56.0	-2946.846212	100.0	-2946.828582	200.0	-2946.824529	200.0	-2946.808528
42.0	-2946.914167			58.0	-2946.846212	150.0	-2946.828582				
44.0	-2946.914167			60.0	-2946.846212	200.0	-2946.828582				
46.0	-2946.914167			100.0	-2946.846212						
48.0	-2946.914167			150.0	-2946.846212						
50.0	-2946.914167			200.0	-2946.846212						
52.0	-2946.914167										
54.0	-2946.914167										
56.0	-2946.914167										
58.0	-2946.914167										
60.0	-2946.914167										
100.0	-2946.914167										
150.0	-2946.914167										
200.0	-2946.914167										
