# Supplementary material for: Benchmark Study of the Electronic States of the LiRb Molecule: Ab Initio Calculations with the Fock Space Coupled Cluster Approach
Source: Molecules. 2023 Nov 17;28(22):7645. doi: 10.3390/molecules28227645 (PMC10675596; doi:10.3390/molecules28227645)
Supplement: Supplementary file 1 [file molecules-28-07645-s001.zip › lirb_unanorccplus_sigma_plus_triplet_asymptotic.pdf]

#R[A]	1^3sigma^+	R[A]	2^3sigma^+	R[A]	3^3sigma^+	R[A]	4^3sigma^+	R[A]	5^3sigma^+	R[A]	6^3sigma^+
1.4	0.380060	1.4	0.430428	1.4	0.441806	1.4	0.462916	1.4	0.469055	1.4	0.471802
1.5	0.283009	1.5	0.335150	1.5	0.343001	1.5	0.364277	1.6	0.303904	1.5	0.375083
1.6	0.215487	1.6	0.269464	1.7	0.226585	1.6	0.296498	1.7	0.257283	1.6	0.308317
1.7	0.168588	1.8	0.192791	1.8	0.192912	1.7	0.250045	1.8	0.224920	1.7	0.261797
1.8	0.135251	1.9	0.168590	1.9	0.170363	1.8	0.218207	1.9	0.202176	1.8	0.228783
1.9	0.111158	2.0	0.150650	2.0	0.153943	2.0	0.181158	2.0	0.185771	1.9	0.204678
2.0	0.093205	2.1	0.136867	2.1	0.141426	2.1	0.170550	2.1	0.171487	2.0	0.186490
2.1	0.079340	2.2	0.125813	2.2	0.131423	2.3	0.147831	2.2	0.161851	2.1	0.173621
2.2	0.068177	2.3	0.116557	2.3	0.118586	2.4	0.137723	2.3	0.150598	2.2	0.163044
2.3	0.058873	2.4	0.106213	2.4	0.108580	2.5	0.128661	2.5	0.131983	2.3	0.157466
2.4	0.050866	2.5	0.095121	2.5	0.101506	2.6	0.120625	2.6	0.124264	2.4	0.150258
2.5	0.043832	2.6	0.085314	2.6	0.095213	2.7	0.113559	2.7	0.117467	2.5	0.141777
2.6	0.037589	2.7	0.076775	2.7	0.089623	2.8	0.107403	2.8	0.111546	2.6	0.134101
2.7	0.032032	2.8	0.069436	2.8	0.084701	2.9	0.102089	2.9	0.106449	2.7	0.127325
2.8	0.027100	2.9	0.063197	2.9	0.080417	3.0	0.097547	3.0	0.102116	2.8	0.121404
2.9	0.022744	3.0	0.057944	3.0	0.076742	3.05	0.095542	3.05	0.100216	2.9	0.116284
3.0	0.018923	3.05	0.055652	3.05	0.075121	3.1	0.093703	3.1	0.098481	3.0	0.111900
3.05	0.017199	3.1	0.053564	3.1	0.073637	3.15	0.092019	3.15	0.096903	3.05	0.109963
3.1	0.015594	3.15	0.051666	3.15	0.072285	3.2	0.090484	3.2	0.095472	3.1	0.108186
3.15	0.014100	3.2	0.049946	3.2	0.071059	3.25	0.089086	3.25	0.094180	3.15	0.106559
3.2	0.012714	3.25	0.048392	3.25	0.069954	3.3	0.087819	3.3	0.093019	3.2	0.105075
3.25	0.011429	3.3	0.046991	3.3	0.068963	3.35	0.086674	3.35	0.091978	3.3	0.102503
3.3	0.010240	3.35	0.045733	3.35	0.068080	3.45	0.084719	3.45	0.090228	3.35	0.101400
3.35	0.009141	3.45	0.043606	3.45	0.066614	3.55	0.083224	3.55	0.088879	3.45	0.099521
3.45	0.007195	3.55	0.041943	3.55	0.065544	3.8	0.080669	3.8	0.086762	3.55	0.098035
3.55	0.005598	3.85	0.039044	3.8	0.064011	3.85	0.080366	3.85	0.086505	3.8	0.095710
3.8	0.002516	3.9	0.038805	3.85	0.063889	3.9	0.080117	3.9	0.086283	3.85	0.095289
3.85	0.002061	3.95	0.038622	3.9	0.063815	3.95	0.079918	3.95	0.086088	3.9	0.094457
3.9	0.001650	4.0	0.038424	3.95	0.063785	4.0	0.079755	4.1	0.085549	3.95	0.093656
3.95	0.001277	4.1	0.038298	4.0	0.063802	4.1	0.079572	4.2	0.085118	4.0	0.092843
4.0	0.000943	4.2	0.038327	4.1	0.063931	4.2	0.079529	4.3	0.084536	4.1	0.091586
4.1	0.000369	4.3	0.038483	4.2	0.064178	4.3	0.079603	4.4	0.083807	4.2	0.090680
4.2	-0.000092	4.4	0.038744	4.3	0.064519	4.4	0.079775	4.5	0.082993	4.3	0.090162
4.3	-0.000457	4.5	0.039090	4.4	0.064932	4.5	0.080025	4.6	0.082223	4.4	0.089999
4.4	-0.000742	4.6	0.039559	4.5	0.065396	4.6	0.080319	4.75	0.081367	4.6	0.090362
4.5	-0.000961	4.7	0.040012	4.6	0.065872	4.7	0.080704	4.8	0.080985	4.7	0.090818
4.6	-0.001114	4.75	0.040262	4.7	0.066395	4.75	0.080895	4.85	0.081107	4.75	0.091052
4.7	-0.001243	4.85	0.040787	4.75	0.066654	4.8	0.080604	4.8	0.081317	4.85	0.091566
4.75	-0.001286	4.9	0.041060	4.8	0.066913	4.85	0.080248	4.9	0.081534	4.9	0.091840
4.8	-0.001322	5.0	0.041621	4.85	0.067169	4.9	0.079909	5.0	0.081979	5.0	0.092411
4.85	-0.001349	5.1	0.041911	4.9	0.067420	5.0	0.079287	5.1	0.082337	5.1	0.093020
4.9	-0.001370	5.2	0.042778	5.0	0.067904	5.1	0.078719	5.2	0.082890	5.2	0.093605
5.0	-0.001394	5.3	0.043362	5.1	0.068294	5.2	0.078294	5.4	0.083783	5.3	0.094210
5.1	-0.001349	5.4	0.043941	5.3	0.069097	5.3	0.077932	5.6	0.084619	5.4	0.094814
5.2	-0.001387	5.5	0.044480	5.4	0.069376	5.4	0.077665	5.7	0.085007	5.5	0.095408
5.3	-0.001364	5.6	0.045042	5.5	0.069590	5.5	0.077478	5.9	0.085711	5.6	0.095995
5.4	-0.001331	5.7	0.045590	5.6	0.069728	5.6	0.077397	6.0	0.086026	5.7	0.096571
5.5	-0.001288	5.8	0.046122	5.7	0.069802	5.8	0.077470	6.2	0.086579	5.8	0.097132
5.6	-0.001243	5.9	0.046636	5.8	0.069819	5.9	0.077600	6.4	0.087034	5.9	0.097676
5.7	-0.001194	6.0	0.047131	5.9	0.069792	6.2	0.078216	6.6	0.087402	6.0	0.098203
5.8	-0.001144	6.2	0.048064	6.0	0.069731	6.4	0.078720	6.8	0.087694	6.2	0.099202
5.9	-0.001091	6.4	0.048915	6.2	0.069548	6.6	0.079243	7.0	0.087925	6.4	0.100123
6.0	-0.001039	6.6	0.049685	6.4	0.069334	6.8	0.079769	7.4	0.088240	6.8	0.101716
6.2	-0.000934	6.8	0.050371	6.6	0.069124	7.0	0.080297	7.6	0.088346	7.0	0.102383
6.4	-0.000833	7.0	0.050979	6.8	0.068939	7.4	0.081227	7.8	0.088430	7.4	0.103430
6.6	-0.000738	7.4	0.052015	7.0	0.068783	7.6	0.081640	7.95	0.088481	7.6	0.103742
6.8	-0.000651	7.6	0.052444	7.4	0.068532	7.95	0.082292	8.0	0.088497	7.8	0.103893
7.0	-0.000572	7.8	0.052820	7.6	0.068440	8.0	0.082379	8.05	0.088511	7.95	0.103928
7.4	-0.000439	7.95	0.053072	7.8	0.068366	8.2	0.082707	8.2	0.088552	8.0	0.103929
7.6	-0.000384	8.0	0.053151	7.95	0.068320	8.35	0.082936	8.35	0.088588	8.05	0.103927
7.8	-0.000336	8.05	0.053227	8.0	0.068306	8.4	0.083009	8.4	0.088599	8.2	0.103904
7.95	-0.000304	8.35	0.053634	8.05	0.068293	8.45	0.083081	8.45	0.088610	8.35	0.103867
8.0	-0.000294	8.4	0.053694	8.2	0.068258	8.5	0.083151	8.5	0.088621	8.4	0.103854
8.05	-0.000284	8.45	0.053752	8.35	0.068229	8.6	0.083286	8.6	0.088642	8.45	0.103840
8.2	-0.000257	8.5	0.053808	8.4	0.068220	8.8	0.083540	8.8	0.088681	8.5	0.103825
8.35	-0.000232	8.6	0.053916	8.45	0.068211	9.01	0.083782	9.01	0.088722	8.8	0.103746
8.4	-0.000225	8.8	0.054110	8.5	0.068203	9.2	0.083982	9.2	0.088758	9.01	0.103703
8.45	-0.000217	9.01	0.054288	8.6	0.068188	9.4	0.084171	9.4	0.088797	9.2	0.103676
8.5	-0.000210	9.2	0.054429	8.8	0.068162	9.6	0.084343	9.6	0.088836	9.4	0.103688
8.6	-0.000197	9.4	0.054555	9.01	0.068139	9.8	0.084498	9.8	0.088877	9.6	0.103683
8.8	-0.000172	9.6	0.054670	9.2	0.068122	10.0	0.084637	10.0	0.088918	9.8	0.103690
9.01	-0.000150	9.8	0.054771	9.4	0.068108	10.2	0.084761	10.2	0.088959	10.0	0.103709
9.2	-0.000133	10.0	0.054860	9.6	0.068096	10.4	0.084871	10.4	0.089001	10.2	0.103738
9.4	-0.000116	10.2	0.054939	9.8	0.068085	10.6	0.084969	10.6	0.089042	10.4	0.103778
9.6	-0.000102	10.4	0.055008	10.0	0.068075	10.8	0.085055	11.2	0.089159	10.6	0.103827
9.8	-0.000090	10.8	0.055123	10.2	0.068066	11.2	0.085195	11.4	0.089196	10.8	0.103883
10.0	-0.000080	11.2	0.055213	10.6	0.068050	11.4	0.085252	11.6	0.089230	11.4	0.104086
10.2	-0.000071	11.4	0.055250	10.8	0.068043	11.8	0.085340	11.8	0.089263	11.6	0.104162
10.4	-0.000063	11.6	0.055283	11.2	0.068030	12.0	0.085370	12.0	0.089294	11.8	0.104237
10.6	-0.000056	11.8	0.055313	11.6	0.068018	12.2	0.085388	12.2	0.089324	12.0	0.104305
10.8	-0.000051	12.0	0.055339	11.8	0.068013	12.4	0.085403	12.4	0.089352	12.2	0.104359
11.2	-0.000041	12.2	0.055360	12.0	0.068008	12.6	0.085417	12.6	0.089377	12.4	0.104427
11.4	-0.000037	12.4	0.055379	12.2	0.068005	12.8	0.085431	12.8	0.089400	12.6	0.104501
11.6	-0.000033	12.6	0.055395	12.4	0.068001	12.9	0.085437	12.9	0.089410	12.8	0.104574
11.8	-0.000030	12.8	0.055410	12.6	0.067998	13.0	0.085442	13.1	0.089431	12.9	0.104610
12.0	-0.000027	12.9	0.055416	12.8	0.067995	13.1	0.085449	13.2	0.089440	13.0	0.104644
12.2	-0.000025	13.0	0.055423	12.9	0.067993	13.2	0.085454	13.3	0.089449	13.1	0.104680
12.4	-0.000022	13.2	0.055434	13.0	0.067992	13.3	0.085459	13.4	0.089458	13.2	0.104714
12.6	-0.000020	13.3	0.055440	13.1	0.067991	13.4	0.085464	13.8	0.089488	13.3	0.104747
12.8	-0.000018	13.4	0.055444	13.2	0.067989	13.8	0.085481	13.9	0.089495	13.4	0.104778
12.9	-0.000018	13.8	0.055463	13.3	0.067988	13.9	0.085485	14.0	0.089501	13.8	0.104902
13.0	-0.000017	13.9	0.055467	13.4	0.067987	14.0	0.085489	14.1	0.089508	13.9	0.104931
13.1	-0.000016	14.0	0.055471	13.8	0.067983	14.1	0.085492	14.2	0.089514	14.3	0.105038
13.2	-0.000015	14.1	0.055474	13.9	0.067982	14.2	0.085495	14.3	0.089519	14.4	0.105063
13.3	-0.000015	14.2	0.055477	14.0	0.067981	14.3	0.085499	14.4	0.089525	17.0	0.105465
13.4	-0.000014	14.3	0.055481	14.1	0.067980	14.4	0.085502	17.0	0.089600	19.0	0.105566
13.5	-0.000013	14.4	0.055484	14.2	0.067979	17.0	0.085556	19.0	0.089620	20.0	0.105591
13.6	-0.000013	19.0	0.055540	14.3	0.067978	19.0	0.085569	20.0	0.089625	21.0	0.105608
13.7	-0.000012	21.0	0.055544	14.4	0.067977	20.0	0.085573	21.0	0.089629	23.0	0.105624
13.8	-0.000012	23.0	0.055548	17.0	0.067963	21.0	0.085576	23.0	0.089633	24.0	0.105628
13.9	-0.000011	24.0	0.055549	19.0	0.067959	23.0	0.085580	26.0	0.089636	26.0	0.105632
14.0	-0.000011	27.0	0.055550	20.0	0.067958	24.0	0.085581	27.0	0.089636	27.0	0.105632
14.1	-0.000011	28.0	0.055550	21.0	0.067957	26.0	0.085583	28.0	0.089637	28.0	0.105634
14.2	-0.000010	29.0	0.055551	23.0	0.067956	27.0	0.085583	29.0	0.089637	29.0	0.105635
14.3	-0.000010	31.0	0.055551	24.0	0.067956	28.0	0.085583	30.0	0.089637	30.0	0.105635
14.4	-0.000010	32.0	0.055551	27.0	0.067955	29.0	0.085584	31.0	0.089637	31.0	0.105636
17.0	-0.000004	33.0	0.055551	28.0	0.067955	30.0	0.085584	32.0	0.089638	32.0	0.105636
19.0	-0.000003	34.0	0.055551	29.0	0.067955	31.0	0.085584	33.0	0.089638	33.0	0.105636
20.0	-0.000002	36.0	0.055551	30.0	0.067955	32.0	0.085584	34.0	0.089638	34.0	0.105637
21.0	-0.000002	37.0	0.055551	31.0	0.067955	33.0	0.085584	36.0	0.089638	36.0	0.105637
23.0	-0.000001	38.0	0.055551	32.0	0.067955	34.0	0.085585	37.0	0.089638	37.0	0.105638
24.0	-0.000001	39.0	0.055551	33.0	0.067955	37.0	0.085585	38.0	0.089638	38.0	0.105638
26.0	-0.000001	40.0	0.055551	34.0	0.067955	38.0	0.085585	39.0	0.089638	39.0	0.105638
27.0	-0.000001	42.0	0.055551	36.0	0.067955	39.0	0.085585	40.0	0.089638	40.0	0.105638
28.0	-0.000001	44.0	0.055551	37.0	0.067955	40.0	0.085585	42.0	0.089638	42.0	0.105639
29.0	-0.000001	46.0	0.055551	38.0	0.067955	42.0	0.085585	44.0	0.089638	44.0	0.105639
30.0	-0.000001	48.0	0.055551	39.0	0.067955	44.0	0.085585	46.0	0.089638	46.0	0.105639
31.0	-0.000001	50.0	0.055551	40.0	0.067955	46.0	0.085585	48.0	0.089638	48.0	0.105639
32.0	-0.000001	52.0	0.055551	42.0	0.067955	48.0	0.085585	50.0	0.089638	50.0	0.105639
33.0	-0.000001	54.0	0.055551	44.0	0.067955	50.0	0.085585	52.0	0.089638	52.0	0.105639
34.0	-0.000001	56.0	0.055551	46.0	0.067955	52.0	0.085585	54.0	0.089638	54.0	0.105639
36.0	-0.000001	58.0	0.055551	48.0	0.067955	54.0	0.085585	56.0	0.089638	56.0	0.105639
37.0	-0.000001	60.0	0.055551	50.0	0.067955	56.0	0.085585	58.0	0.089638	58.0	0.105639
38.0	-0.000001	100.0	0.055551	52.0	0.067955	58.0	0.085585	100.0	0.089638	100.0	0.105639
39.0	-0.000001	150.0	0.055551	54.0	0.067955	60.0	0.085585	150.0	0.089638	150.0	0.105639
40.0	0.000000	200.0	0.055551	56.0	0.067955	100.0	0.085585	200.0	0.089638	200.0	0.105639
42.0	0.000000			58.0	0.067955	150.0	0.085585				
44.0	0.000000			60.0	0.067955	200.0	0.085585				
46.0	0.000000			100.0	0.067955						
48.0	0.000000			150.0	0.067955						
50.0	0.000000			200.0	0.067955						
52.0	0.000000										
54.0	0.000000										
56.0	0.000000										
58.0	0.000000										
60.0	0.000000										
100.0	0.000000										
150.0	0.000000										
200.0	0.000000										
