# Supplementary material for: Benchmark Study of the Electronic States of the LiRb Molecule: Ab Initio Calculations with the Fock Space Coupled Cluster Approach
Source: Molecules. 2023 Nov 17;28(22):7645. doi: 10.3390/molecules28227645 (PMC10675596; doi:10.3390/molecules28227645)
Supplement: Supplementary file 1 [file molecules-28-07645-s001.zip › supplementary_material.pdf]

Supplementary material for

Benchmark study of the electronic states of the LiRb molecule: Ab initio calculations with the Fock space coupled cluster approach

Grzegorz Skrzyński [*], Monika Musial [†]
Institute of Chemistry
University of Silesia in Katowice
Szkolna 9, 40-006 Katowice, Poland

November 16, 2023

[*]Corresponding author: e-mail address: grzegorz.skrzynski@us.edu.pl
[†]Corresponding author: e-mail address: monika.musial@us.edu.pl

The following files were attachted as the Supplementary material:

Table S1: Total IH-FS-CCSD (2,0)/unANO-RCC+ energy values (a.u.) for $^1\Sigma^+$ states of LiRb. (File: lirb_unanorccplus_sigma_plus_singlet)

Table S2: Total IH-FS-CCSD (2,0)/unANO-RCC+ energy values (a.u.) for $^3\Sigma^+$ states of LiRb. (File: lirb_unanorccplus_sigma_plus_triplet)

Table S3: Total IH-FS-CCSD (2,0)/unANO-RCC+ energy values (a.u.) for $^1\Pi$ and $^1\Delta$ states of LiRb. (File: lirb_unanorccplus_pi_delta_singlet)

Table S4: Total IH-FS-CCSD (2,0)/unANO-RCC+ energy values (a.u.) for $^3\Pi$ and $^3\Delta$ states of LiRb. (File: lirb_unanorccplus_pi_delta_triplet)

Table S5: Total IH-FS-CCSD (2,0) DK3/Sapporo-DKH3-QZP-2012-diffuse energy values (a.u.) for $^1\Sigma^+$ states of LiRb. (File: lirb_sapporo_sigma_plus_singlet)

Table S6: Total IH-FS-CCSD (2,0) DK3/Sapporo-DKH3-QZP-2012-diffuse energy values (a.u.) for $^3\Sigma^+$ states of LiRb. (File: lirb_sapporo_sigma_plus_triplet)

Table S7: Total IH-FS-CCSD (2,0) DK3/Sapporo-DKH3-QZP-2012-diffuse energy values (a.u.) for $^1\Pi$ and $^1\Delta$ states of LiRb. (File: lirb_sapporo_pi_delta_singlet)

Table S8: Total IH-FS-CCSD (2,0) DK3/Sapporo-DKH3-QZP-2012-diffuse energy values (a.u.) for $^3\Pi$ and $^3\Delta$ states of LiRb. (File: lirb_sapporo_pi_delta_triplet)

Table S9: Total IH-FS-CCSD (2,0)/unANO-RCC+ energy values (a.u.) (E + 2946.914167 a.u.) for $^1\Sigma^+$ states of LiRb. (File: lirb_unanorccplus_sigma_plus_singlet_asymptotic)

Table S10: Total IH-FS-CCSD (2,0)/unANO-RCC+ energy values (a.u.) (E + 2946.914167 a.u.) for $^3\Sigma^+$ states of LiRb. (File: lirb_unanorccplus_sigma_plus_triplet_asymptotic)

Table S11: Total IH-FS-CCSD (2,0)/unANO-RCC+ energy values (a.u.) (E + 2946.914167 a.u.) for $^1\Pi$ and $^1\Delta$ states of LiRb. (File: lirb_unanorccplus_pi_delta_singlet_asymptotic)

Table S12: Total IH-FS-CCSD (2,0)/unANO-RCC+ energy values (a.u.) (E + 2946.914167 a.u.) for $^3\Pi$ and $^3\Delta$ states of LiRb. (File: lirb_unanorccplus_pi_delta_triplet)

Table S13: Total IH-FS-CCSD (2,0) DK3/Sapporo-DKH3-QZP-2012-diffuse energy values (a.u.) (E + 2986.954313 a.u.) for $^1\Sigma^+$ states of LiRb. (File: lirb_sapporo_sigma_plus_singlet_asymptotic)

Table S14: Total IH-FS-CCSD (2,0) DK3/Sapporo-DKH3-QZP-2012-diffuse energy values (a.u.) (E + 2986.954313 a.u.) for $^3\Sigma^+$ states of LiRb. (File: lirb_sapporo_sigma_plus_triplet_asymptotic)

Table S15: Total IH-FS-CCSD (2,0) DK3/Sapporo-DKH3-QZP-2012-diffuse energy values (a.u.) (E + 2986.954313 a.u.) for $^1\Pi$ and $^1\Delta$ states of LiRb. (File: lirb_sapporo_pi_delta_singlet_asymptotic)

Table S16: Total IH-FS-CCSD (2,0) DK3/Sapporo-DKH3-QZP-2012-diffuse energy values (a.u.) (E + 2986.954313 a.u.) for $^3\Pi$ and $^3\Delta$ states of LiRb. (File: lirb_sapporo_pi_delta_triplet_asymptotic)

Figure S1: Potential energy curves of LiRb calculated using the IH-FS-CCSD(2,0)/unANO-RCC+ method for the six lowest dissociation limits. Energy related to the dissociation limit of the ground state. (File: figS1)

Figure S2: Potential energy curves of LiRb calculated using the IH-FS-CCSD(2,0) DK3/Sapporo-DKH3-QZP-2012-diffuse method for the six lowest dissociation limits. Energy related to the dissociation limit of the ground state. (File: figS2)
