# Supplementary figures and images for: Benchmark Study of the Electronic States of the LiRb Molecule: Ab Initio Calculations with the Fock Space Coupled Cluster Approach
Source: Molecules. 2023 Nov 17;28(22):7645. doi: 10.3390/molecules28227645 (PMC10675596; doi:10.3390/molecules28227645)

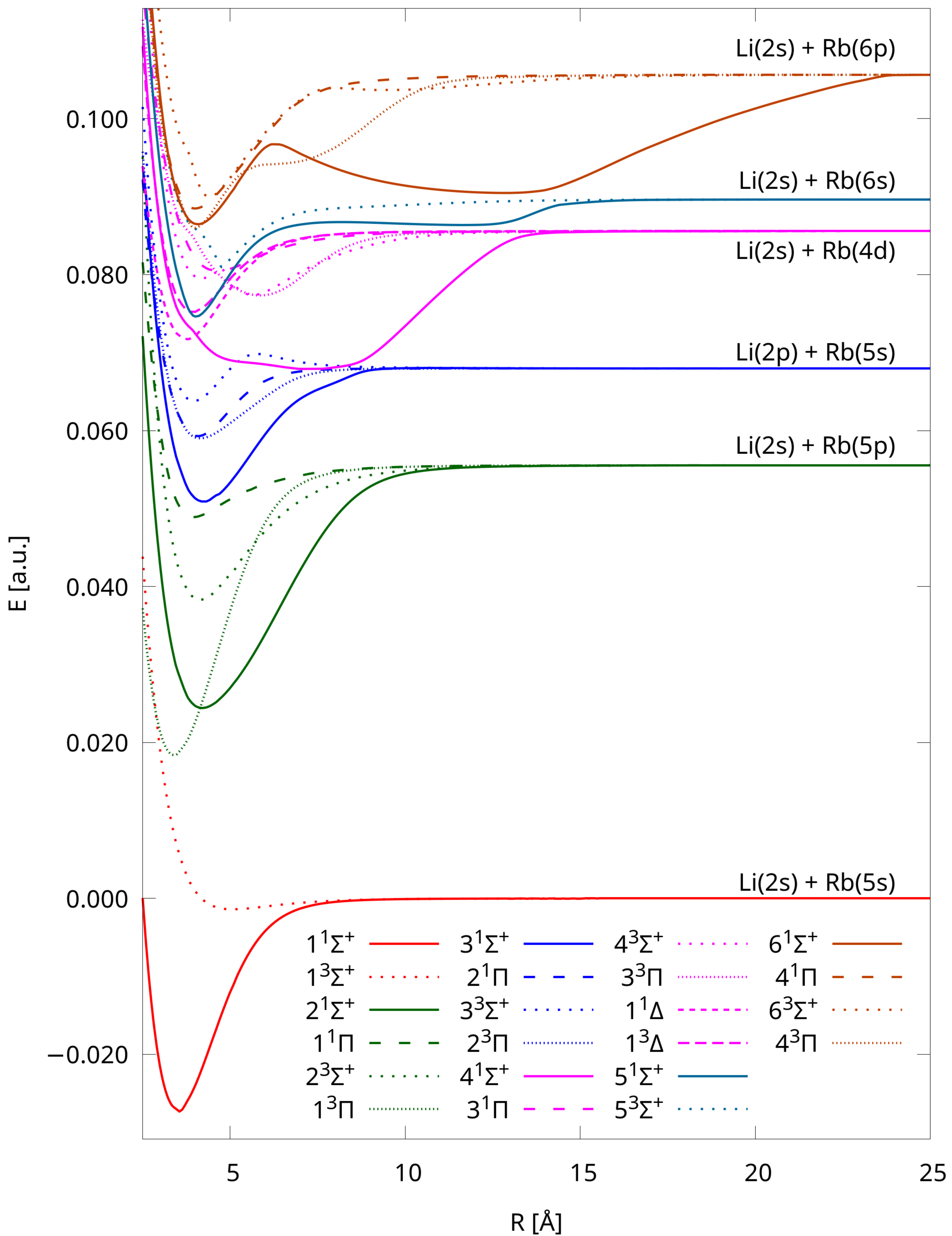

Supplement: Supplementary file 1 [file molecules-28-07645-s001.zip › figS1.pdf]

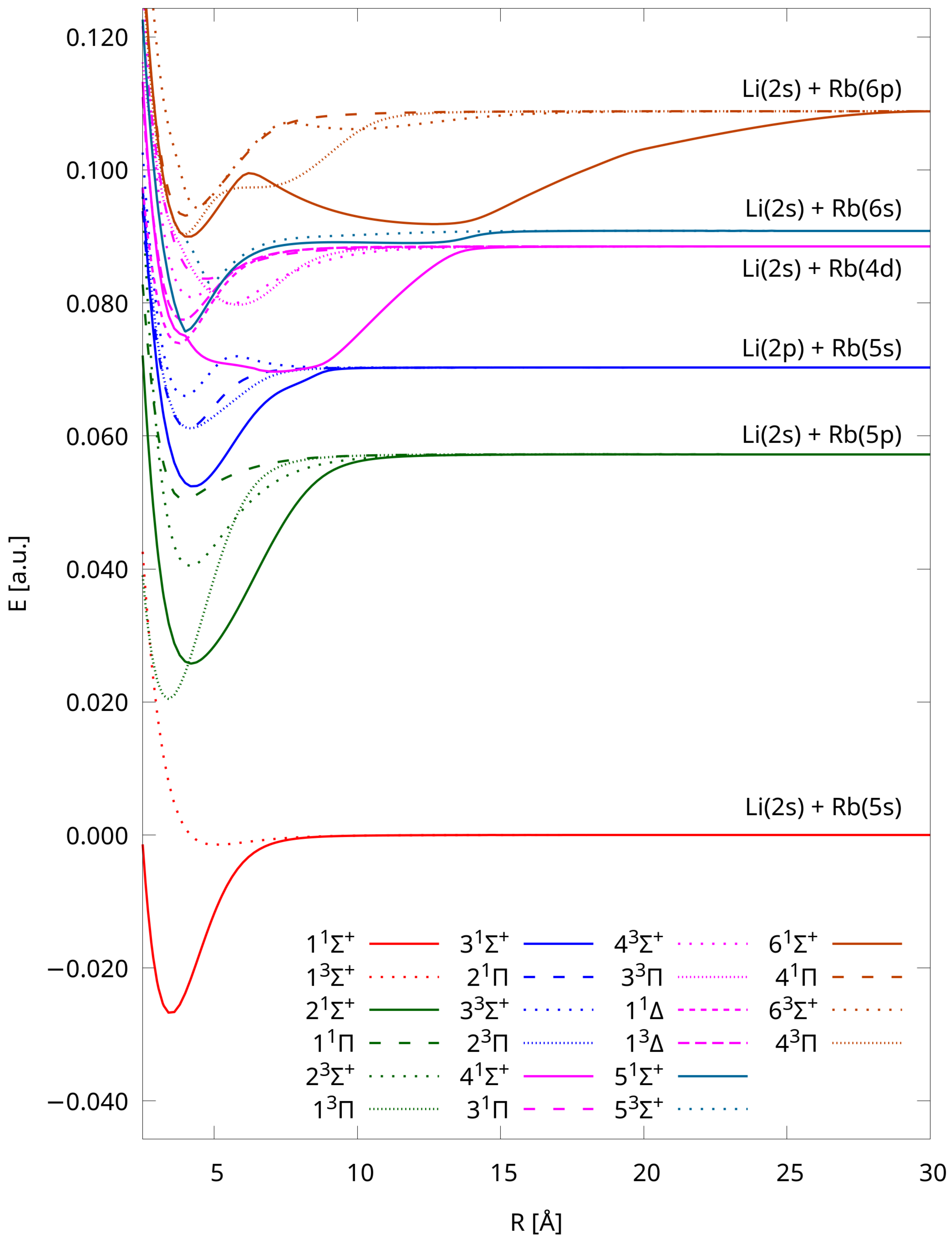

Supplement: Supplementary file 1 [file molecules-28-07645-s001.zip › figS2.pdf]
